# Mechanochemical Dehydrogenative Phenochalcogenazination: A Pronounced Water Effect

**DOI:** 10.1002/cssc.70801

**Published:** 2026-06-17

**Authors:** Alina Paffen, Calogero Quaranta, Kishor Gavhane, Hardik V. Mori, Frederic W. Patureau, Carsten Bolm

**Affiliations:** ^1^ Institute of Organic Chemistry RWTH Aachen University Aachen Germany; ^2^ PI Industries Ltd. Udaipur India

**Keywords:** cross dehydrogenative coupling, dehydrogenative phenochalcogenazination, mechanochemistry, persulfate oxidation, water‐accelerated reaction

## Abstract

A mechanochemical phenochalcogenazination (X = O, S, Se) of phenols and related compounds in the ball mill is reported. The reaction proceeds without a catalyst, and the triarylamine‐based products are obtained in up to 99% yield in 60 minutes reaction time. The use of water‐doped silica as an additive proved critical for the robustness and the reproducibility of the process.

## Graphical abstract

We report herein the first organic solvent‐free mechanochemical dehydrogenative phenochalcogenazination reaction, with a persulfate salt as a terminal oxidant and wetted silica as a crucial additive.

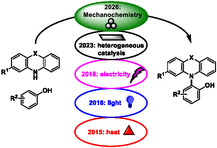

## 1 Introduction

**SCHEME 1:**
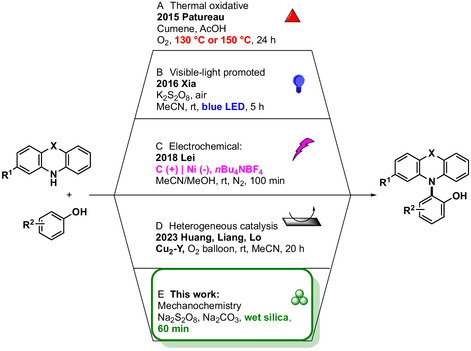
Selected utilized techniques for the dehydrogenative phenochalcogenazination reaction.

Cross dehydrogenative coupling (CDC) reactions are particularly attractive to synthetic chemists due to the avoidance of pre‐activation steps on both coupling partners, as well as their usually pronounced atom‐economical character [1, 2, 3, 4, 5, 6, 7, 8, 9]. Solvent‐free mechanochemical CDCs, however, remain underdeveloped [10]. One associated challenge is the design of efficient oxidizing strategies within the ball mill, which are necessary to make CDCs thermodynamically feasible. In this context, we envisioned that the dehydrogenative phenochalcogenazination reaction, a highly specific and functional group‐tolerant CDC reaction discovered by some of us in 2015 (Scheme 1A) [11], might be transposable to solvent‐free mechanochemical conditions [12, 13, 14, 15, 16, 17, 18, 19, 20, 21, 22, 23, 24, 25, 26, 27, 28, 29, 30, 31, 32, 33, 34, 35]. The resulting method would then feature both the step and atom economy (AE) characteristics of CDCs on the one hand, and the solvent‐free conditions of ball milling on the other, thus considerably enhancing its sustainable nature [36, 37, 38, 39]. While photochemical (Scheme 1B) [40, 41, 42], electrochemical (Scheme 1C) [43, 44, 45, 46, 47], as well as homogeneous [48, 49, 50, 51, 52, 53, 54, 55, 56, 57, 58] and heterogeneous catalytic (Scheme 1D) [59] versions of the dehydrogenative phenochalcogenazination reaction have been extensively investigated, this study demonstrates the first mechanochemical method for that reaction (Scheme 1E). It proceeds fast (60 min) under mild conditions and is catalyst‐free.

## 2 Results and Discussion

### 2.1 Reaction Optimization

We commenced our study with an initial condition screening, using 0.5 mmol of 10*H*‐phenothiazine **1a** (PTZ) and 3.0 equiv. of 4‐*tert*‐butylphenol (**2a**) as model substrates. Na_2_CO_3_ was selected as the base (3.0 equiv.), Na_2_S_2_O_8_ as oxidant (1.5 equiv.), and silica as grinding auxiliary. The reaction setup involved 10 mL tungsten carbide (WC) jars in a mixer mill, using one 10 mm WC ball at 30 Hz for 30 min. As hoped for, the transformation proceeded, but to our dismay, we encountered reproducibility issues under these conditions, leading to variable yields of the targeted C—N dehydrogenative coupling product **3aa**. Eventually, after some investigations, we suspected a correlation between the level of atmospheric humidity and the coupling efficiency. To verify this hypothesis, a defined amount of water (10 wt%) was added to pre‐dried silica, and the resulting wetted silica was applied as an additive in the coupling process (for details, see the Supporting Information). Of note, this protocol led to a ratio of solvent volume and sample weight *η* of <0.03 μL mg^−1^ and could therefore be considered as LAG (liquid‐assisted grinding) conditions [24, 60, 61]. As a result of this water addition, both better yield and higher reproducibility were achieved (Table 1, entry 1, **3aa**: 89%). Increasing the water content of silica further to 20 wt% yielded 90% of the product (Table 1, entry 2), which is within the range of the results obtained with 10 wt% (Table 1, entry 1). Notably, a higher water content of 40 wt% wetted silica decreased the reaction yield to 76%, which could be attributed to a more pronounced change in the rheology of the reaction system (Table 1, entry 2). Based on these results, we chose to proceed with 10 wt% wetted silica, as the optimal water range appears to lie between 10 wt% and 20 wt%. This also provides a safety margin, accounting for potential moisture uptake by silica upon storage if not properly sealed. In testing the reaction conditions, it was found that the absence of silica resulted in a higher yield (83%) compared to using dry silica (53%, Table 1, entry 2). Reproducing these control reactions gave similar results, although here the yields can fluctuate by around ten percentage points, which is in line with our earlier observation that “water‐free” conditions can be associated with reproducibility issues. In any case, the trend that dry silica performs worse than no silica remained. Doubling the amount of H_2_O‐doped silica to 15.6 equiv. resulted in the same yield (89%) as with 7.8 equiv.

**TABLE 1:** Optimization of the reaction conditions.^a^

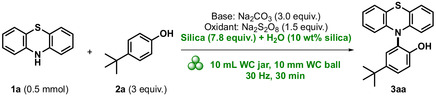		
Entry	Variation from optimized conditions	Yield of 3aa, %
1	None	89 (average over 4 reactions: 85/88/89/92)
2	Silica + H_2_O (20 wt%)/silica + H_2_O (40 wt%)/no silica/dry silica (no water added)/15.6 equiv. of silica + H_2_O (10 wt%)	90/76/83 [75]/53 [64] /89
3	No oxidant/NaIO_4_/PIDA/Na_2_S_2_O_8_ (1.0 equiv.)	nr (95% recovery of **1a**)^b^/29/75/40
4	No base/NaHCO_3_/Na_2_HPO_4_/Na_2_CO_3_ (1.0 equiv.)	3/61/57/61
5	25 Hz/**60 min**/ZrO_2_‐Y/SS/five balls (Ø = 7 mm)	89/**94**/87/92/56
6	Addition of talcum (14 mg)/addition of talcum (50 mg)	90/92
7	K_2_CO_3_ and K_2_S_2_O_8_	47
8	**2a** (1.0 equiv.)	60

a In a 10 mL tungsten carbide (WC) jar**, 1a** (100 mg, 0.5 mmol, 1.0 equiv.), **2a** (225 mg, 1.5 mmol, 3.0 equiv.), Na_2_CO_3_ (159 mg, 1.5 mmol, 3.0 equiv.), Na_2_S_2_O_8_ (179 mg, 0.75 mmol, 1.5 equiv.), silica (3.90 mmol, 7.8 equiv.), and 10 wt% of H_2_O (257 mg) were loaded. One 10 mm WC ball was added, and the reaction mixture was milled in a MM400 mixer mill (Retsch) for 30 minutes at 30 Hz. Yields shown in square brackets refer to reproduced reactions. All yields refer to the product amount after isolation by column chromatography.

b A 60‐minute reaction time instead of 30 minutes. nr = not reacted.

Next, we verified that the use of an external oxidant was strictly necessary for the reaction to occur (Table 1, entry 3). Indeed, no product formation was observed when the reaction proceeded just under air, allowing 95% recovery of **1a**. As previously [48, 53], NaIO_4_ and PIDA were competent oxidants in the reaction, albeit with decreased yields of 29% and 75% of **3aa**, respectively. A lower oxidant loading of 1.0 equiv. instead of 1.5 equiv. resulted in a substantially decreased product yield of 40% (Table 1, entry 3). In the absence of a base, almost no reaction occurred (3% of **3aa**, Table 1, entry 4). Changing the base from Na_2_CO_3_ to NaHCO_3_ or Na_2_HPO_4_, or decreasing the amount of Na_2_CO_3_ to only 1.0 equiv. led to inferior product yields (Table 1, entry 4). Next, the milling parameters were analyzed (Table 1, entry 5). A decreased frequency of 25 Hz resulted in the same yield as for 30 Hz (89% of **3aa**). After 60 min of milling, the yield increased to 94%. Changing the WC equipment to jars and balls (10 mm) of ZrO_2_‐Y or stainless steel (SS) led to similar yields for **3aa** (87% and 92%, respectively), which also proved that possible steel‐ or tungsten leaching was not relevant for the reactivity. Using five 7 mm WC balls instead of one 10 mm WC ball decreased the yield to 56%, probably due to a lower impact of each ball. Adding talcum as a solid lubricant [62, 63] increased the yield slightly to 90% with 14 mg and to 92% with 50 mg of talcum (Table 1, entry 6). Since the effect of talcum was barely noticeable, its addition was not considered further in the subsequent studies. A change of the counterion from sodium to potassium (with K_2_CO_3_ and K_2_S_2_O_8_ as reagents) almost cut the yield in half to 47% (Table 1, entry 7). Finally, decreasing the loading of **2a** from 3.0 equiv. to 1.0 equiv. resulted in only 60% yield of **3aa** (Table 1, entry 8).

The fine‐tuning of the final reaction parameters was performed using both SS and WC jars, for 30 and 60 minutes with 4‐*tert*‐butylphenol (**2a**), *p*‐cresol (4‐methylphenol) (**2b**), and 4‐phenylphenol (**2c**) as test substrates (Table 2). While SS showed promising results at 30 min reaction time, a WC/60‐min reaction time combination was found overall superior. Hence, the latter was selected for the investigation of the substrate scope.

**TABLE 2:** Evaluation of reaction time versus ball milling material.^a^

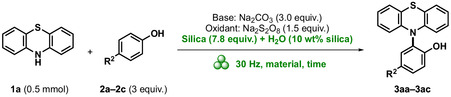					
Entry	R^2^	Yield, % @ 30 min		Yield, % @ 60 min	
		SS	WC	SS	WC
1	*t*Bu (**3aa**)	95	89	84	94
2	Me (**3ab**)	56	52	93	88
3	Ph (**3ac**)	42	54	50	66

a In a 10 mL tungsten carbide (WC) or stainless steel (SS) jar**, 1a** (100 mg, 0.5 mmol, 1.0 equiv.), **2a**–**c** (1.5 mmol, 3.0 equiv.), Na_2_CO_3_ (159 mg, 1.5 mmol, 3.0 equiv.), Na_2_S_2_O_8_ (179 mg, 0.75 mmol, 1.5 equiv.), silica (3.90 mmol, 7.8 equiv.), and 10 wt% of H_2_O (257 mg) were loaded. One 10 mm WC or SS ball was added, and the reaction mixture was milled in a MM400 mixer mill (Retsch) for 30 or 60 min at 30 Hz. The yields refer to the product amount after isolation by column chromatography.

### 2.2 Substrate Scope

**SCHEME 2:**
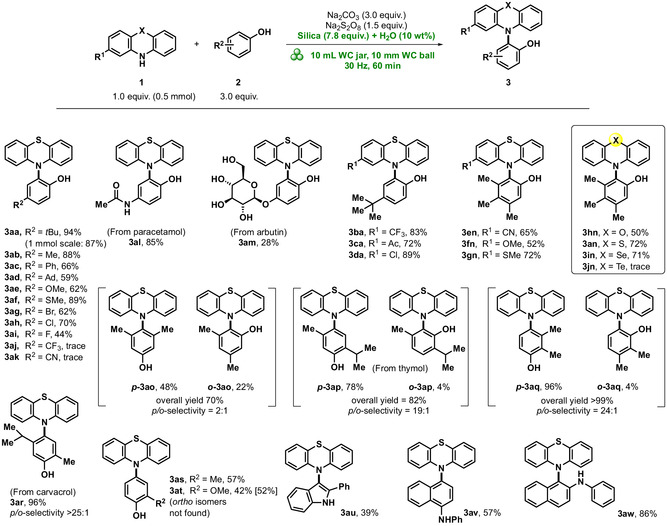
Substrate scope. Yields in squared brackets refer to the reaction under standard conditions with 2 h of milling time instead of 1 h.

Next, the substrate scope of the mechanochemical method was evaluated (Scheme 2). Phenols bearing alkyl (**3aa**, **3ab**, **3ad**) or aryl (**3ac**) substituents, as well as methoxy‐ (**3ae**) and thiomethyl‐groups (**3af**) in the 4‐position, reacted well. Furthermore, 4‐halogenated phenols worked under the mechanochemical conditions without changing the reaction parameters, resulting in yields from 44% for 4‐fluoro phenol (to give **3ai**) to 70% for 4‐chloro phenol (providing **3ah**). Unfortunately, electron‐withdrawing trifluoromethyl and nitrile groups in the *para* position of the phenol afforded only traces of the corresponding products **3aj** and **3ak**. Gratifyingly, paracetamol afforded **3al** in 85% yield. Moreover, unprotected sugar‐containing phenol arbutin led to an encouraging yield of 28% for coupling product **3am**.

In addition, several 2‐substituted phenothiazines were found to be well tolerated, bearing trifluoromethyl, acetyl, chloro, cyano, methoxy, and thiomethyl groups (52%–89%, **3ba–da** and **3en–gn**). Moreover, it was found that phenoxazine **1h** (X = O) and phenoselenazine **1i** (X = Se), respectively the lighter and heavier chalcogen congeners of **1a**, were also accommodated to give **3hn** (50%) and **3in** (71%). For phenotellurazine **1j** (X = Te), bearing the heaviest nonradioactive chalcogen, only traces of the corresponding product **3jn** were observed.

Importantly, the dehydrogenative mechanochemical phenothiazination method revealed a high *para*‐selectivity in cases wherein both *ortho* and *para* positions were available on the phenolic substrate. For example, when 3,5‐dimethyl phenol (**2o**) was applied, products ***p*‐3ao** and ***o*‐3ao** were isolated in an overall yield of 70% with a *p/o*‐selectivity of 2:1. Thymol (2‐isopropyl‐5‐methylphenol, **2p**), a pleasant olfactory phenol that can be extracted from thyme, resulted in an overall yield of 82% of ***p*‐3ap** and ***o*‐3ap**, with an impressive *p/o*‐selectivity of 19:1. Furthermore, 2,3‐dimethylphenol (**2q**) afforded ***p*‐3aq** and ***o*‐3aq** in >99% yield, with an even greater *p/o*‐selectivity of 24:1. For carvacrol (2‐methyl‐5‐isopropylphenol, **2r**), a constitutional isomer of thymol, only the *para*‐phenothiazinated product **3ar** was found, in 96% yield. We thus estimate that the *para*‐selectivity for that substrate must exceed >25:1. The observed *p/o*‐selectivities are consistent with previous methods operating in solution [57, 64]. These might originate from subtle steric effects, to which bulky phenochalcogenazines are likely susceptible. When other simple 2‐substituted phenols were applied, such as *ortho*‐cresol (**2s**), or guaiacol (2‐methoxyphenol, **2t**), only the *para*‐product could be isolated with yields of 57% (**3as**) and 42% (**3at**). Doubling the reaction time afforded **3at** in a modestly increased 52% yield. The lower yields for these last two products may be due to yet unidentified side reactions. Notably, anilines and indoles could also be coupled with phenothiazine **1a**. 2‐Phenylindole (**2u**), for example, resulted in the corresponding C3—N dehydrogenative coupling product **3au** in encouraging 39% yield. Both *N*‐phenylnaphthalen‐1‐amine (**2v**) and *N*‐phenylnaphthalen‐2‐amine (**2w**) reacted as well to provide **3av** in 57% and **3aw** in 86% yield.

### 2.3 Mechanism

**SCHEME 3:**
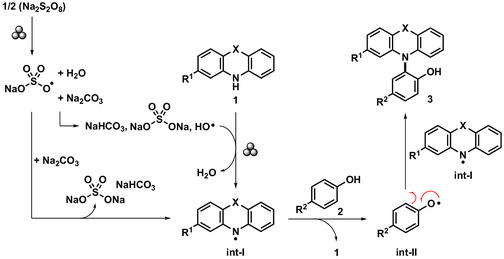
Proposed mechanism.

The mechanism of the reaction is expected to involve a classical radical pathway [65]. First, the persulfate salt undergoes homolytic dissociation (Scheme 3), probably being supported by the mechanical force induced by the milling [66]. The resulting sulfate radical can decay under basic aqueous conditions to give a reactive hydroxyl radical [67, 68]. This may explain why the herein featured mechanochemical method is so dependent on the presence of both a base and some water. Next, the hydroxyl radical initiates the reaction with phenochalcogenazine **1** by a HAT step, thereby providing neutral, persistent N‐radical intermediate **int‐I**. Its reaction with phenol **2** furnishes phenol radical species **int‐II**. Alternatively, **int‐II** can also be formed by the reaction of **2** with another hydroxyl radical. Product **3** is then formed by radical/radical recombination from **Int‐I** and **Int‐II**. Alternatively, the persulfate radical may directly initiate the HAT step to furnish **int‐I**, particularly in water‐free conditions (Table 1, entry 2). Furthermore, indole **2u** and anilines **2v and** **2w** may react via a similar mechanism as phenols.

### 2.4 Quantitative Sustainability Assessment

The sustainability of the mechanochemical phenochalcogenazination in the ball mill was evaluated using AE, Environmental Impact Factor (E factor), and Process Mass Intensity (PMI); for details, see the SI. As a comparison in solution, the photochemical reaction conditions reported by Xia in 2016 were chosen, because these represent a similarly simple set‐up while also utilizing a persulfate salt as terminal oxidant [40]. The AE parameter for the present mechanochemical method was found to be similar to that of Xia's method because of the similar oxidants, which only differ in terms of sodium versus potassium salts. In contrast, both E factor and PMI were found more than one order of magnitude lower for the mechanochemical method due to the absence of a solvent, which thus represents a considerable advancement in terms of sustainability [69, 70, 71].

## 3 Conclusion

In conclusion, we developed an organic solvent‐free mechanochemical dehydrogenative phenochalcogenazination reaction, with a persulfate salt as the terminal oxidant. The presence of both a carbonate base and water proved essential for high and reproducible yields, possibly due to the intermediacy of the hydroxyl radical. The scope of the reaction is broad and, to a large degree, consistent with non‐mechanochemical methods. These findings may furnish a blueprint for the implementation of other sustainable CDC reactions in solvent‐free ball milling setups.

## Funding

This work was supported by the Deutsche Forschungsgemeinschaft (Grant PA 2395/5‐1) and PI Industries.

## Conflicts of Interest

The authors declare no conflicts of interest.

## Supporting information

The authors have cited additional references within the Supporting Information [72, 73]. The Supporting Information includes notably detailed experimental procedures, analytical data and NMR spectra.

## Data Availability

The data that support this finding of this study are available in the supplementary material of this article.
