# Supplementary material for: Mechanochemical Dehydrogenative Phenochalcogenazination: A Pronounced Water Effect
Source: ChemSusChem. 2026 Jun 17;19(12):e70801. doi: 10.1002/cssc.70801 (PMC13274814; doi:10.1002/cssc.70801)

Supporting information

Mechanochemical Dehydrogenative Phenochalcogenazination: A Pronounced Water Effect

Alina Paffen[+1] | Calogero Quaranta[+1] | Kishor Gavhane[1,2] | Hardik V. Mori[1,2] | Frederic W. Patureau[1] | Carsten Bolm[1]

[1]Institute of Organic Chemistry, RWTH Aachen University, Landoltweg 1, 52074 Aachen, Germany | [2] PI Industries Ltd., Udaisagar Road, Udaipur, 313001 Rajasthan, India

[+] These authors contributed equally to this work.

frederic.patureau@rwth-aachen.de, carsten.bolm@oc.rwth-aachen.de

Contents

1. General information (p. S2)

2. Experimental procedures (p. S2)

3. Quantitative Sustainability Assessment (p. S3)

4. Product characterization (p. S4)

5. Literature (p. S19)

6. NMR spectra (p. S21)

1. General information

All chemicals were purchased from commercial suppliers (Sigma-Aldrich, TCI, BLDpharm, Thermo Fisher Scientific, Alfa Aesar, abcr, fluorochem, chemPUR, Grüssing) and used without purification unless stated otherwise. Phenoselenazine **1i** (X = Se) and phenotellurazine **1j** (X = Te) were synthetized according to literature procedures.[S1,S2] *n*-Hexane was distilled prior to use.

Mechanochemical reactions were carried out in a MM400 Mixer Mill from RETSCH. Unless stated otherwise, a 10 mL jar and a 10 mm ball were used.

Analytical thin-layer chromatography (TLC) was performed on silica F254 aluminum plates from VWR international. The substances were visualized by UV light λ = 254 nm. Silica 60 M (0.04–0.063 mm) that was used for silica gel column chromatography and as grinding auxiliary was purchased from Macherey-Nagel. The solvent ratios for column chromatographic purifications are given in volume shares.

NMR analysis was performed at room temperature on a Bruker Avance Neo 400, Bruker Avance Neo 600, Agilent VNMRS 400 or an Agilent VNMRS 600 spectrometer. The evaluation of the obtained NMR spectra was performed using MestReNova software by MESTRELAB RESEARCH. Chemical shifts (δ) are given in ppm (parts per million) and are referenced to the respective residual solvent peak (DMSO-d_6: ^{1}H: δ = 2.50 ppm, ^{13}C: δ = 39.52 ppm; $CDCl_3$: ^{1}H: δ = 7.26 ppm, ^{13}C: δ = 77.16 ppm). Coupling constants (J) are given in Hertz (Hz).

High-resolution mass spectra (HRMS) were recorded on a Thermo Scientific LTQ Orbitrap XL (ESI), or Finnigan MAT95 (EI, 70 eV).

IR spectra were measured on a PerkinElmer 100 FT-IR spectrometer with an UATR Diamond/KRS-5 crystal with attenuated total reflectance (ATR).

2. Experimental procedures

Preparation of silica + 10 wt% of H_2O

Silica 60 M (0.04–0.063 nm) was dried in a Schlenk tube in an oil bath at 150 °C for 3 h at high vacuum. The dried silica gel was then stored under argon until use.

Silica gel (4000 mg) was mixed with 400 µL of H_2O. The bottle was sealed and shaken until no chunks were visible and the silica showed its original texture.

Preparation of silica + 20 wt% of H_2O

Silica 60 M (0.04–0.063 nm) was dried in a Schlenk tube in an oil bath at 150 °C at high vacuum overnight. The dried silica gel was then stored under argon until use.

Silica gel (2000 mg) was mixed with 400 µL of H_2O. The bottle was sealed and shaken until no chunks were visible and the silica showed its original texture.

Preparation of silica + 40 wt% of H_2O

Silica 60 M (0.04–0.063 nm) was dried in a Schlenk tube in an oil bath at 150 °C at high vacuum overnight. The dried silica gel was then stored under argon until use.

Silica gel (2000 mg) was mixed with 800 µL of H_2O. The bottle was sealed and shaken until no chunks were visible and the silica showed its original texture.

Cleaning of ball mill jars

During the optimization, it became clear that proper cleaning of the jars is necessary to obtain reproducible yields.

The crude reaction mixture can be of different morphologies. Hard and sticky mixtures need to be scraped out thoroughly. Remaining residues can be removed by soaking in ethyl acetate and additional scraping with a spatula. The solvent is then removed *in vacuo,* and the crude can be subjected to column chromatography.

General procedure GP1

Phenothiazine **1** (0.5 mmol, 1.0 equiv.), phenol **2** (1.5 mmol, 3.0 equiv.), Na_2CO_3 (159 mg, 1.5 mmol, 3.0 equiv.), $Na_2S_2O_8$ (179 mg, 0.75 mmol, 1.5 equiv.), and silica + 10 wt% of H_2O (257 mg, 3.9 mmol SiO_2, 7.8 equiv.) were loaded together with one 10 mm tungsten carbide (WC) ball into a 10 mL WC jar. The reaction was milled in a MM400 mixer mill for 60 minutes at 30 Hz. The crude reaction mixture was scraped out of the jar and remaining residues were dissolved in ethyl acetate. The solvent was removed *in vacuo,* and the crude reaction mixture was purified by column chromatography to yield the respective product.

General procedure GP 2: Scale up

10*H*-Phenothiazine **1a** (199 mg, 1.0 mmol, 1.0 equiv.), 4-*tert*-butylphenol **2aa** (450 mg, 3.0 mmol, 3.0 equiv.), Na_2CO_3 (318 mg, 3.0 mmol, 3.0 equiv.), $Na_2S_2O_8$ (357 mg, 1.5 mmol, 1.5 equiv), and silica + 10 wt% H_2O (514 mg, 7.8 mmol, 7.8 equiv.) were weighed into a 10 mL tungsten carbide (WC) jar and one 10 mm WC ball was added. The reaction was placed in a MM400 mixer mill for 60 minutes at 30 Hz. The crude reaction mixture was scraped out of the jar and remaining residues were dissolved in ethyl acetate. The solvent was removed *in vacuo* and the crude reaction mixture was purified *via* column chromatography using *n*-hexane:DCM in a 6:4 ratio to yield the respective product **3aa** as colorless foam (303 mg, 87%).

3. Quantitative Sustainability Assessment

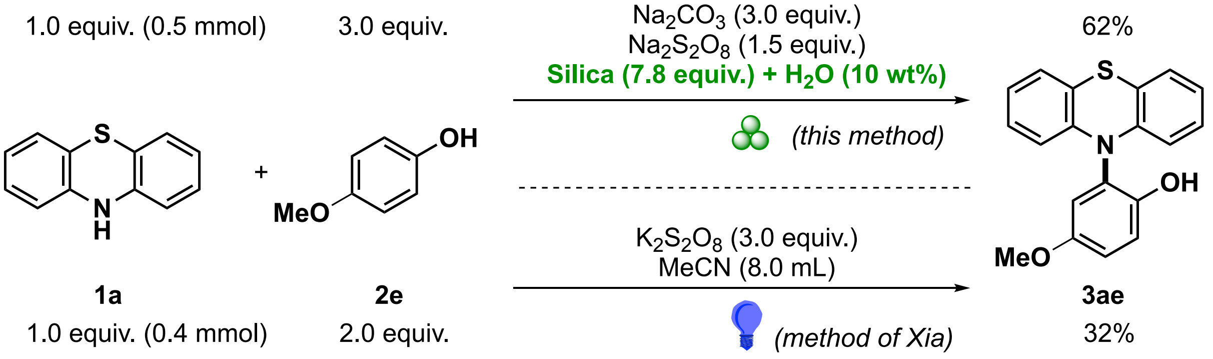

Entry	Sustainability Value	This Method	Xia 2016[S3]
1	AE [%]	57	54
2	E factor [kg/kg]	7.6	163.5
3	PMI [kg/kg]	8.8	164.5

$$\text{Atom Economy (AE)} = \frac{MW(Product)\cdot 100}{\sum MW(Substrates + Oxidant)}\ [\%]$$ [S4-S6]

$$\text{Environmental Impact Factor (E factor)} = \frac{\sum m(Input\ Materials\ excl.\ Water) - m(Product)}{m(Product)} \left[\frac{\text{kg}}{\text{kg}}\right]$$ [S4-S6]

$$\text{Process Mass Intensity (PMI)} = \frac{\sum m(Input\ Materials\ incl.\ Water)}{m(Product)} \left[\frac{\text{kg}}{\text{kg}}\right]$$ [S4-S6]

4. Product characterization

4-(*tert*-Butyl)-2-(10*H*-phenothiazin-10-yl)phenol (3aa)

Compound **3aa** was synthesized according to GP1 from 10*H*-phenothiazine **1a** (100 mg, 0.5 mmol, 1.0 equiv.) and 4-*tert*-butyl phenol **2a** (225 mg, 1.5 mmol, 3.0 equiv.). The product was obtained as pale-beige foam (163 mg, 0.47 mmol, 94%) after column chromatography using *n*-hexane:DCM in a 6:4 ratio.

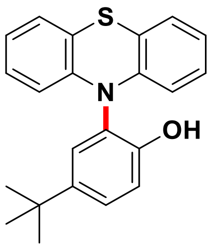

^{1}H-NMR (600 MHz, DMSO-d_6): δ = 9.73 (s, 1H), 7.40 (dd, J = 8.5, 2.5 Hz, 1H), 7.16 (d, J = 2.5 Hz, 1H), 7.07 (d, J = 8.6 Hz, 1H), 6.99 (dd, J = 7.6, 1.6 Hz, 2H), 6.89 (ddd, J = 8.5, 7.3, 1.6 Hz, 2H), 6.79 (td, J = 7.4, 1.2 Hz, 2H), 6.05 (dd, J = 8.3, 1.2 Hz, 2H), 1.25 (s, 9H) ppm.

^{13}C{^{1}H}-NMR (151 MHz, DMSO-d_6): δ = 153.1, 143.7, 143.0, 127.4, 127.3, 126.9, 126.2, 125.7, 122.1, 118.4, 116.7, 115.4, 33.9, 31.3 ppm.

The NMR data are in accordance with those reported in the literature.[S7]

4-Methyl-2-(10*H*-phenothiazin-10-yl)phenol (3ab)

Compound **3ab** was synthesized according to GP1 from 10*H*-phenothiazine **1a** (100 mg, 0.5 mmol, 1.0 equiv.) and *p*-cresol **2b** (163 mg, 1.5 mmol, 3.0 equiv.). The product was obtained as colorless solid (135 mg, 0.44 mmol, 88%) after column chromatography using *n*-hexane:DCM in a 6:4 ratio.

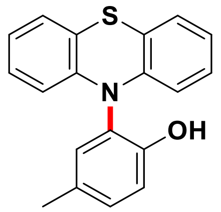

^{1}H-NMR (600 MHz, DMSO-*d*$_6$): δ = 9.68 (s, 1H), 7.17 (dd, *J* = 8.3, 2.2 Hz, 1H), 7.04 (d, *J* = 2.2 Hz, 1H), 7.02 (d, *J* = 8.2 Hz, 1H), 6.98 (dd, *J* = 7.6, 1.5 Hz, 2H), 6.89 (ddd, *J* = 8.5, 7.3, 1.6 Hz, 2H), 6.78 (td, *J* = 7.4, 1.2 Hz, 2H), 6.06 (dd, *J* = 8.2, 1.2 Hz, 2H), 2.25 (s, 3H) ppm.

^{13}C{^{1}H}-NMR (151 MHz, DMSO-*d*$_6$): δ = 153.2, 142.8, 131.3, 130.6, 130.0, 127.2, 126.2, 126.1, 122.1, 118.3, 117.0, 115.4, 19.9 ppm.

The NMR data are in accordance with those reported in the literature.[S8]

3-(10*H*-Phenothiazin-10-yl)-[1,1‘-biphenyl]-4-ol (3ac)

Compound **3ac** was synthesized according to GP1 from 10*H*-phenothiazine **1a** (100 mg, 0.5 mmol, 1.0 equiv.) and 4-phenyl phenol **2c** (256 mg, 1.5 mmol, 3.0 equiv.). The product was obtained as colorless solid (122 mg, 0.33 mmol, 66%) after column chromatography using *n*-hexane:DCM in a 6:4 ratio.

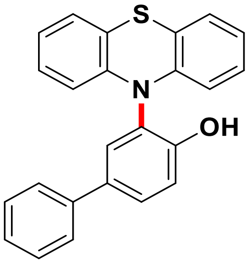

^{1}H-NMR (400 MHz, DMSO-*d*$_6$): δ = 10.13 (s, 1H), 7.73 (dd, *J* = 8.6, 2.4 Hz, 1H), 7.65 (d, *J* = 8.0 Hz, 2H), 7.55 (d, *J* = 2.4 Hz, 1H), 7.40 (t, *J* = 7.7 Hz, 2H), 7.29 (t, *J* = 7.4 Hz, 1H), 7.23 (d, *J* = 8.5 Hz, 1H), 7.00 (d, *J* = 7.6 Hz, 2H), 6.91 (t, *J* = 7.8 Hz, 2H), 6.80 (t, *J* = 7.4 Hz, 2H), 6.14 (d, *J* = 8.3 Hz, 2H) ppm.

^{13}C{^{1}H}-NMR (151 MHz, DMSO-*d*$_6$): δ = 155.2, 142.8, 138.9, 133.1, 129.2, 128.9, 128.3, 127.3, 126.88, 126.85, 126.2, 126.0, 122.2, 118.4, 117.9, 115.4 ppm.

The NMR data are in accordance with those reported in the literature.[S7]

4-(Adamantan-1-yl)-2-(10*H*-phenothiazin-10-yl)phenol (3ad)

Compound **3ad** was synthesized according to GP1 from 10*H*-phenothiazine **1a** (100 mg, 0.5 mmol, 1.0 equiv.) and 4-(adamantan-1-yl)phenol **2d** (343 mg, 1.5 mmol, 3.0 equiv.). The product was obtained as pale-blue oil (126 mg, 0.30 mmol, 59%) after column chromatography using *n*-hexane:DCM in a 6:4 ratio.

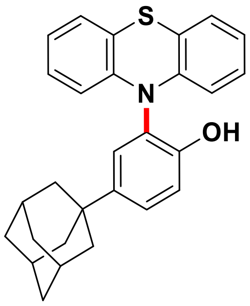

^{1}H-NMR (600 MHz, DMSO-*d$_6$*): δ = 9.72 (s, 1H), 7.35 (dd, *J* = 8.5, 2.5 Hz, 1H), 7.13 (d, *J* = 2.5 Hz, 1H), 7.07 (d, *J* = 8.6 Hz, 1H), 6.99 (dd, *J* = 7.6, 1.6 Hz, 2H), 6.89 (ddd, *J* = 8.5, 7.3, 1.6 Hz, 2H), 6.78 (td, *J* = 7.4, 1.3 Hz, 2H), 6.04 (dd, *J* = 8.3, 1.2 Hz, 2H), 2.01 (sept, *J* = 3.1 Hz, 3H), 1.83 (d, *J* = 3.1 Hz, 6H), 1.70 (t, *J* = 3.2 Hz, 6H) ppm.

^{13}C{^{1}H}-NMR (151 MHz, DMSO-*d$_6$*): δ = 153.1, 144.1, 143.0, 127.3, 127.1, 126.3, 126.2, 125.9, 122.1, 118.4, 116.8, 115.4, 42.8, 36.1, 35.2, 28.3 ppm.

HRMS (ESI, m/z): calculated for $C_{28}H_{27}NOS^+$ (M^+): 425.1808, found: 425.1795.

IR (neat, cm^{-1}): 3788, 3062, 2900, 2847, 2654, 2576, 2324, 2107, 1988, 1931, 1769, 1728, 1586, 1508, 1456, 1368, 1305, 1239, 1123, 1022, 998, 925, 823, 742.

4-Methoxy-2-(10*H*-phenothiazin-10-yl)phenol (3ae)

Compound **3ae** was synthesized according to GP1 from 10*H*-phenothiazine **1a** (100 mg, 0.5 mmol, 1.0 equiv.) and 4-methoxyphenol **2e** (187 mg, 1.5 mmol, 3.0 equiv.). The product was obtained as colorless solid (100 mg, 0.31 mmol, 62%) after column chromatography using *n*-hexane:DCM in a 6:4 ratio.

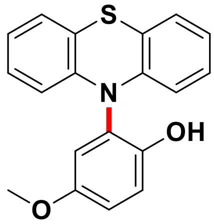

^{1}H-NMR (600 MHz, DMSO-*d$_6$*): δ = 9.44 (s, 1H), 7.06 (d, *J* = 8.9 Hz, 1H), 7.00 - 6.96 (m, 3H), 6.90 (ddd, *J* = 8.5, 7.3, 1.6 Hz, 2H), 6.81 - 6.77 (m, 3H), 6.08 (dd, *J* = 8.3, 1.2 Hz, 2H), 3.70 (s, 3H) ppm.

^{13}C{^{1}H}-NMR (151 MHz, DMSO-*d$_6$*): δ = 153.5, 149.3, 142.7, 127.3, 126.4, 126.2, 122.2, 118.2, 117.8, 116.2, 115.4, 115.4, 55.5 ppm.

The NMR data are in accordance with those reported in the literature.[S7]

4-Methylthio-2-(10*H*-phenothiazin-10-yl)phenol (3af)

Compound **3af** was synthesized according to GP1 from 10*H*-phenothiazine **1a** (100 mg, 0.5 mmol, 1.0 equiv.) and 4-methylthiophenol **2f** (210 mg, 1.5 mmol, 3.0 equiv.). The product was obtained as colorless solid (151 mg, 0.45 mmol, 89%) after column chromatography using *n*-hexane:DCM in a 6:4 ratio.

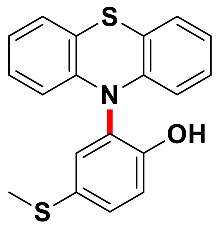

^{1}H-NMR (600 MHz, DMSO-d_6): δ = 9.99 (s, 1H), 7.33 (dd, J = 8.6, 2.5 Hz, 1H), 7.15 (d, J = 2.4 Hz, 1H), 7.11 (d, J = 8.5 Hz, 1H), 6.99 (dd, J = 7.6, 1.6 Hz, 2H), 6.91 (ddd, J = 8.5, 7.3, 1.6 Hz, 2H), 6.80 (td, J = 7.4, 1.2 Hz, 2H), 6.07 (dd, J = 8.3, 1.2 Hz, 2H), 2.43 (s, 3H) ppm.

^{13}C{^{1}H}-NMR (151 MHz, DMSO-d_6): δ = 153.7, 142.6, 129.9, 129.6, 128.8, 127.3, 127.1, 126.2, 122.3, 118.4, 118.0, 115.3, 16.3 ppm.

The NMR data are in accordance with those reported in the literature.[S1]

4-Bromo-2-(10*H*-phenothiazin-10-yl)phenol (3ag)

Compound **3ag** was synthesized according to GP1 from 10*H*-phenothiazine **1a** (100 mg, 0.5 mmol, 1.0 equiv.) and 4-bromophenol **2g** (260 mg, 1.5 mmol, 3.0 equiv.). The product was obtained as pale-blue solid (115 mg, 0.31 mmol, 62%) after column chromatography using *n*-hexane:DCM in a 6:4 ratio.

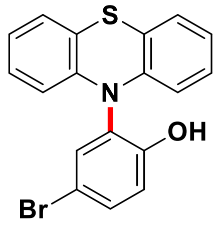

^{1}H-NMR (600 MHz, DMSO-d_6): δ = 10.32 (s, 1H), 7.56 (dd, J = 8.8, 2.6 Hz, 1H), 7.45 (d, J = 2.6 Hz, 1H), 7.11 (d, J = 8.8 Hz, 1H), 7.00 (dd, J = 7.6, 1.5 Hz, 2H), 6.92 (ddd, J = 8.6, 7.3, 1.6 Hz, 2H), 6.82 (td, J = 7.4, 1.2 Hz, 2H), 6.06 (dd, J = 8.3, 1.2 Hz, 2H) ppm.

^{13}C{^{1}H}-NMR (151 MHz, DMSO-d_6): δ = 155.4, 142.3, 133.9, 133.1, 128.1, 127.3, 126.3, 122.5, 119.3, 118.5, 115.3, 110.6 ppm.

The NMR data are in accordance with those reported in the literature.[S9]

4-Chloro-2-(10*H*-phenothiazin-10-yl)phenol (3ah)

Compound **3ah** was synthesized according to GP1 from 10*H*-phenothiazine **1a** (100 mg, 0.5 mmol, 1.0 equiv.) and 4-chlorophenol **2h** (192 mg, 1.5 mmol, 3.0 equiv.). The product was obtained as pale-green solid (114 mg, 0.35 mmol, 70%) after column chromatography using *n*-hexane:DCM in a 6:4 ratio.

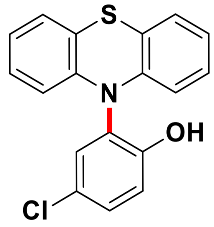

^{1}H-NMR (600 MHz, DMSO-d_6): δ = 10.30 (s, 1H), 7.44 (dd, J = 8.8, 2.7 Hz, 1H), 7.35 (d, J = 2.7 Hz, 1H), 7.16 (d, J = 8.8 Hz, 1H), 7.00 (dd, J = 7.6, 1.6 Hz, 2H), 6.91 (ddd, J = 8.5, 7.3, 1.6 Hz, 2H), 6.81 (td, J = 7.5, 1.2 Hz, 2H), 6.06 (dd, J = 8.3, 1.2 Hz, 2H) ppm.

^{13}C{^{1}H}-NMR (151 MHz, DMSO-d_6): δ = 154.9, 142.3, 131.1, 130.2, 127.6, 127.3, 126.3, 123.5, 122.4, 118.6, 118.4, 115.2 ppm.

The NMR data are in accordance with those reported in the literature.[S9]

4-Fluoro-2-(10*H*-phenothiazin-10-yl)phenol (3ai)

Compound **3ai** was synthesized according to GP1 from 10*H*-phenothiazine **1a** (100 mg, 0.5 mmol, 1.0 equiv.) and 4-fluorophenol **2i** (169 mg, 1.5 mmol, 3.0 equiv.). The product was obtained as colorless solid (68 mg, 0.22 mmol, 44%) after column chromatography using *n*-hexane:DCM in a 6:4 ratio.

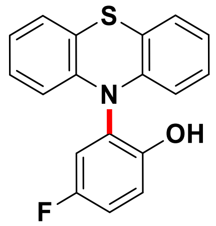

^{1}H-NMR (600 MHz, DMSO-d_6): δ = 9.96 (s, 1H), 7.26 (td, J = 8.6, 3.1 Hz, 1H), 7.18 (dd, J = 8.8, 3.0 Hz, 1H), 7.13 (dd, J = 9.1, 5.4 Hz, 1H), 7.00 (dd, J = 7.6, 1.5, 2H), 6.91 (td, J = 8.1, 1.3 Hz, 2H), 6.81 (td, J = 7.4, 1.2 Hz, 2H), 6.07 (dd, J = 8.2, 1.3 Hz, 2H) ppm.

^{13}C{^{1}H}-NMR (151 MHz, DMSO-d_6): δ = 155.9 (d, J = 236.9 Hz), 152.2 (d, J = 1.9 Hz), 142.4, 127.3, 126.7 (d, J = 10.1 Hz), 126.3, 122.4, 118.4, 117.9 (d, J = 8.6 Hz), 117.9 (d, J = 22.2 Hz), 116.9 (d, J = 22.2 Hz), 115.2 ppm.

^{19}F-NMR (564 MHz, DMSO-d_6): δ = –122.85 to –122.93 (m) ppm.

The NMR data are in accordance with those reported in the literature.[S10]

N-[4-Hydroxy-3-(10*H*-phenothiazin-10-yl)phenyl]acetamide (3al)

Compound **3al** was synthesized according to GP1 from 10*H*-phenothiazine **1a** (100 mg, 0.5 mmol, 1.0 equiv.) and paracetamol **2l** (227 mg, 1.5 mmol, 3.0 equiv.). The product was obtained as pale-yellow solid (148 mg, 0.42 mmol, 85%) after column chromatography using *n*-hexane:ethyl acetate in a 2:1 ratio.

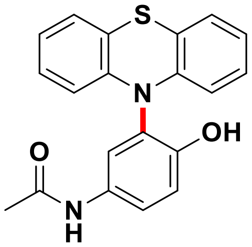

^{1}H-NMR (400 MHz, DMSO-d_6): δ = 9.87 (s, 1H), 9.72 (s, 1H), 7.55 (d, J = 2.6 Hz, 1H), 7.48 (dd, J = 8.8, 2.6 Hz, 1H), 7.06 (d, J = 8.8 Hz, 1H), 6.99 (dd, J = 7.6, 1.6 Hz, 2H), 6.90 (td,

J = 7.8, 1.7 Hz, 2H), 6.80 (td, J = 7.5, 1.2 Hz, 2H), 6.09 (dd, J = 8.2, 1.3 Hz, 2H), 1.99 (s, 3H) ppm.

$^{13}C\{^1H\}$-NMR (101 MHz, DMSO-d_6): δ = 167.8, 151.2, 142.7, 133.0, 127.2, 126.2, 125.9, 122.2, 121.7, 121.1, 118.3, 117.2, 115.4, 23.8 ppm.

The NMR data are in accordance with those reported in the literature.[S9]

(2*S*,3*R*,4*S*,5*S*,6*R*)-2-[4-Hydroxy-3-(10*H*-phenothiazin-10-yl)phenoxy]-6-(hydroxymethyl)-tetrahydro-2*H*-pyran-3,4,5-triol (3am)

Compound **3am** was synthesized according to GP1 from 10*H*-phenothiazine **1a** (100 mg, 0.5 mmol, 1.0 equiv.) and arbutin **2m** (408 mg, 1.5 mmol, 3.0 equiv.). The product was obtained as grey-blue solid (66 mg, 0.14 mmol, 28%) after column chromatography using DCM:methanol in a 7:1 ratio.

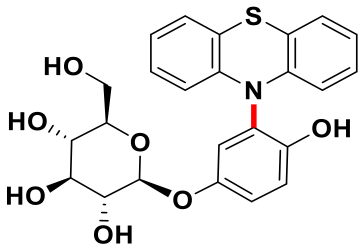

^1H-NMR (600 MHz, DMSO-d_6): δ = 9.57 (s, 1H), 7.10 (dd, J = 9.0, 2.9 Hz, 1H), 7.05 (d, J = 8.9 Hz, 1H), 6.99 (dd, J = 7.5, 1.6 Hz, 2H), 6.90 (td, J = 7.4, 1.6 Hz, 3H), 6.80 (td, J = 7.5, 1.2 Hz, 2H), 6.11 (d, J = 8.3 Hz, 2H), 5.26 (br s, 1H), 4.98 (br s, 2H), 4.78 (d, J = 7.7 Hz, 1H), 4.52 (br s, 1H), 3.64 (dd, J = 11.8, 2.1 Hz, 1H), 3.45 (dd, J = 11.8, 5.6 Hz, 1H), 3.30 - 3.11 (m, 4H) ppm.

$^{13}C\{^1H\}$-NMR (151 MHz, DMSO-d_6): δ = 151.4, 150.3, 142.7, 127.3, 126.4, 126.2, 122.2, 119.1, 118.5, 118.3, 117.5, 115.4, 101.4, 76.9, 76.4, 73.4, 69.7, 60.7 ppm.

The NMR data are in accordance with those reported in the literature.[S10]

4-(*tert*-Butyl)-2-[2-(trifluoromethyl)-10*H*-phenothiazin-10-yl]phenol (3ba)

Compound **3ba** was synthesized according to GP1 from 2-(trifluoromethyl)-10*H*-phenothiazine **1b** (134 mg, 0.5 mmol, 1.0 equiv.) and 4-*tert*-butyl phenol **2a** (225 mg, 1.5 mmol, 3.0 equiv.). The product was obtained as colorless foam (172 mg, 0.41 mmol, 83%) after column chromatography using *n*-hexane:DCM in a 6:4 ratio.

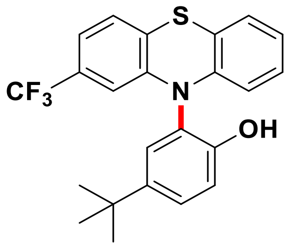

^1H-NMR (600 MHz, DMSO-d_6): δ = 9.89 (s, 1H), 7.43 (dd, J = 8.5, 2.5 Hz, 1H), 7.24 - 7.19 (m, 2H), 7.13 - 7.08 (m, 2H), 7.04 (dd, J = 7.5, 1.4 Hz, 1H), 6.94 (ddd, J = 8.6, 7.3, 1.6 Hz, 1H),

6.84 (td, J = 7.5, 1.1 Hz, 1H), 6.19 (d, J = 1.9 Hz, 1H), 6.04 (dd, J = 8.3, 1.2 Hz, 1H), 1.26 (s, 9H) ppm.

^{13}C{^{1}H}-NMR (151 MHz, DMSO-d_6): δ = 152.7, 144.0, 143.6, 142.2, 127.8 (q, J = 31.7 Hz), 127.8, 127.4, 127.3, 127.0, 126.4, 124.9, 124.2, 123.9 (q, J = 271.8 Hz), 122.9, 118.5 (q, J = 3.4 Hz), 117.4, 116.9, 115.7, 110.7 (q, J = 3.9 Hz), 33.9, 31.2 ppm.

^{19}F-NMR (564 MHz, DMSO-d_6): δ = –61.82 (s) ppm.

The NMR data are in accordance with those reported in the literature.[S7]

1-{10-[5-(*tert*-Butyl)-2-hydroxyphenyl]-10*H*-phenothiazin-2-yl}ethan-1-one (3ca)

Compound **3ca** was synthesized according to GP1 from 2-acetyl-10*H*-phenothiazine **1c** (121 mg, 0.5 mmol, 1.0 equiv.) and 4-*tert*-butyl phenol **2a** (225 mg, 1.5 mmol, 3.0 equiv.). The product was obtained as yellow solid (140 mg, 0.36 mmol, 72%) after column chromatography using toluene:DCM in a 3:1 ratio.

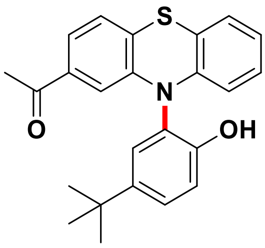

^{1}H-NMR (600 MHz, DMSO-d_6): δ = 9.79 (s, 1H), 7.44 – 7.40 (m, 2H), 7.18 (d, J = 2.3 Hz, 1H), 7.13 (d, J = 7.9 Hz, 1H), 7.09 (d, J = 8.4 Hz, 1H), 7.00 (dd, J = 7.5, 1.5 Hz, 1H), 6.91 (ddd, J = 8.6, 7.2, 1.6 Hz, 1H), 6.81 (td, J = 7.4, 1.3 Hz, 1H), 6.59 (d, J = 1.8 Hz, 1H), 6.02 (dd, J = 7.9, 1.9 Hz, 1H), 2.34 (s, 3H), 1.26 (s, 9H) ppm.

^{13}C{^{1}H}-NMR (151 MHz, DMSO-d_6): δ = 196.7, 152.9, 143.8, 143.0, 142.5, 135.8, 127.7, 127.4, 127.1, 126.3, 126.2, 125.6, 125.3, 123.1, 122.5, 117.4, 116.9, 115.6, 113.3, 33.9, 31.3, 26.3 ppm.

The NMR data are in accordance with those reported in the literature.[S10]

4-(*tert*-Butyl)-2-(2-chloro-10*H*-phenothiazin-10-yl)phenol (3da)

Compound **3da** was synthesized according to GP1 from 2-chloro-10*H*-phenothiazine **1d** (117 mg, 0.5 mmol, 1.0 equiv.) and 4-*tert*-butyl phenol **2a** (225 mg, 1.5 mmol, 3.0 equiv.). The product was obtained as colorless oil (170 mg, 0.45 mmol, 89%) after column chromatography using *n*-hexane:DCM in a 6:4 ratio.

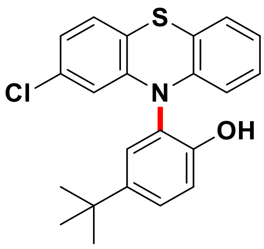

^{1}H-NMR (600 MHz, DMSO-d_6): δ = 9.87 (s, 1H), 7.43 (dd, J = 7.6, 2.6 Hz, 1H), 7.18 (d, J = 2.5 Hz, 1H), 7.09 (d, J = 8.6 Hz, 1H), 7.04 - 7.00 (m, 2H), 6.92 (t, J = 8.3 Hz, 1H), 6.87 - 6.81 (m, 2H), 6.05 (dd, J = 8.3, 1.2 Hz, 1H), 5.98 (d, J = 2.2 Hz, 1H), 1.26 (s, 9H) ppm.

^{13}C{^{1}H}-NMR (151 MHz, DMSO-d_6): δ = 152.8, 144.4, 144.0, 142.3, 131.7, 127.6, 127.5, 127.3, 127.2, 126.4, 125.1, 122.8, 121.7, 118.1, 117.7, 116.9, 115.7, 114.7, 33.9, 31.3 ppm.

The NMR data are in accordance with those reported in the literature.[S7]

10-(6-Hydroxy-2,3,4-trimethylphenyl)-10*H*-phenothiazine-2-carbonitrile (3en)

Compound **3en** was synthesized according to GP1 from 10*H*-phenothiazine-2-carbonitrile **1e** (112 mg, 0.5 mmol, 1.0 equiv.) and 3,4,5-trimethyl phenol **2n** (205 mg, 1.5 mmol, 3.0 equiv.). The product was obtained as bright yellow foam (117 mg, 0.33 mmol, 65%) after column chromatography using *n*-hexane:ethyl acetate in a 4:1 ratio, followed by column chromatography using toluene:DCM in a 10:1 ratio.

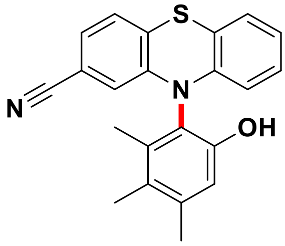

^{1}H-NMR (600 MHz, DMSO-d_6): δ = 9.59 (s, 1H), 7.18 (dd, J = 7.9, 1.6 Hz, 1H), 7.15 (d, J = 8.0 Hz, 1H), 6.98 (dd, J = 7.6, 1.5 Hz, 1H), 6.89 (ddd, J = 8.5, 7.3, 1.6 Hz, 1H), 6.83 - 6.79 (m, 2H), 6.04 (d, J = 1.6 Hz, 1H), 5.95 (dd, J = 8.3, 1.2 Hz, 1H), 2.27 (s, 3H), 2.11 (s, 3H), 2.10 (s, 3H) ppm.

^{13}C{^{1}H}-NMR (151 MHz, DMSO-d_6): δ = 152.3, 142.7, 140.9, 138.1, 135.6, 128.0, 127.2, 126.4, 126.1, 125.7, 123.1, 122.1, 118.8, 117.1, 116.3, 115.8, 115.5, 109.5, 20.6, 15.3, 14.1 ppm.

The NMR data are in accordance with those reported in the literature.[S7]

2-(2-Methoxy-10*H*-phenothiazin-10-yl)-3,4,5-trimethylphenol (3fn)

Compound **3fn** was synthesized according to GP1 from 2-methoxy-10*H*-phenothiazine **1f** (115 mg, 0.5 mmol, 1.0 equiv.) and 3,4,5-trimethyl phenol **2n** (204 mg, 1.5 mmol, 3.0 equiv.). The product was obtained as pale-pink foam (98 mg, 0.27 mmol, 54%) after column chromatography using pure toluene.

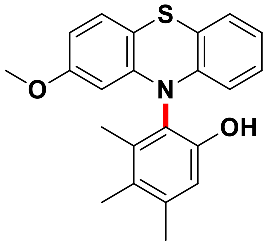

^{1}H-NMR (600 MHz, DMSO-d_6): δ = 9.39 (s, 1H), 6.94 (dd, J = 7.6, 1.5 Hz, 1H), 6.87 (d, J = 8.4 Hz, 1H), 6.84 (ddd, J = 8.5, 7.3, 1.6 Hz, 1H), 6.76 (s, 1H), 6.75 (td, J = 7.4, 1.3 Hz, 1H),

6.41 (dd, J = 8.4, 2.6 Hz, 1H), 5.98 (dd, J = 8.3, 1.2 Hz, 1H), 5.54 (d, J = 2.6 Hz, 1H), 3.54 (s, 3H), 2.26 (s, 3H), 2.09 (s, 3H), 2.07 (s, 3H) ppm.

^{13}C{^{1}H}-NMR (151 MHz, DMSO-d_6): δ = 159.1, 152.5, 143.2, 141.7, 137.4, 135.8, 127.1, 126.7, 126.6, 126.0, 123.1, 122.1, 118.7, 115.5, 115.1, 109.3, 105.7, 103.1, 55.0, 20.6, 15.3, 14.2 ppm.

HRMS (ESI, m/z): calculated for $C_{22}H_{21}O_2NSNa^+$ ($[M+Na]^+$): 386.1185, found: 386.1193.

IR (neat, cm^{-1}): 3806, 3367, 3064, 2918, 2843, 2328, 2108, 1985, 1833, 1581, 1492, 1458, 1330, 1296, 1262, 1159, 1129, 1074, 1028, 957, 914, 839, 784, 739, 681.

3,4,5-Trimethyl-2-[2-(methylthio)-10*H*-phenothiazin-10-yl]phenol (3gn)

Compound **3gn** was synthesized according to GP1 from 2-methylthio-10*H*-phenothiazine **1g** (123 mg, 0.5 mmol, 1.0 equiv.) and 3,4,5-trimethyl phenol **2n** (204 mg, 1.5 mmol, 3.0 equiv.). The product was obtained as colorless foam (136 mg, 0.36 mmol, 72%) after column chromatography using pure toluene.

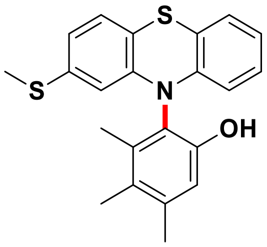

^{1}H-NMR (600 MHz, DMSO-d_6): δ = 9.46 (s, 1H), 6.96 (dd, J = 7.6, 1.6 Hz, 1H), 6.93 (d, J = 8.0 Hz, 1H), 6.86 (ddd, J = 8.5, 7.3, 1.6 Hz, 1H), 6.78 (s, 1H), 6.77 (td, J = 7.4, 1.4 Hz, 1H), 6.69 (dd, J = 8.0, 2.0 Hz, 1H), 5.97 (dd, J = 8.2, 1.3 Hz, 1H), 5.88 (d, J = 2.0 Hz, 1H), 2.26 (s, 6H), 2.10 (s, 3H), 2.09 (s, 3H) ppm.

^{13}C{^{1}H}-NMR (151 MHz, DMSO-d_6): δ = 152.7, 142.4, 141.9, 137.6, 136.8, 135.7, 127.3, 126.8, 126.6, 126.2, 122.9, 122.3, 119.5, 118.4, 115.5, 115.3, 115.2, 113.1, 20.6, 15.3, 15.0, 14.2 ppm.

HRMS (ESI, m/z): calculated for $C_{22}H_{21}ONS_2Na^+$ ($[M+Na]^+$): 402.0957, found: 402.0963.

IR (neat, cm^{-1}): 3414, 2917, 2325, 2103, 2002, 1886, 1581, 1461, 1388, 1295, 1237, 1160, 1112, 1074, 1039, 1002, 950, 903, 854, 796, 744, 672.

3,4,5-Trimethyl-2-(10*H*-phenoxazin-10-yl)phenol (3hn)

Compound **3hn** was synthesized according to GP1 from 10*H*-phenoxazine **1h** (92 mg, 0.5 mmol, 1.0 equiv.) and 3,4,5-trimethyl phenol **2n** (205 mg, 1.5 mmol, 3.0 equiv.). The product was obtained as colorless solid (80 mg, 0.25 mmol, 50%) after column chromatography using *n*-hexane:DCM in a 6:4 ratio, followed by column chromatography using toluene:DCM in a 2:1 ratio.

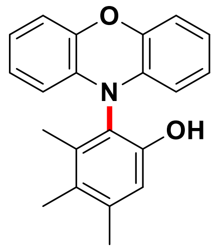

^{1}H-NMR (600 MHz, DMSO-*d$_6$*): δ = 9.30 (s, 1H), 6.74 (s, 1H), 6.68 - 6.62 (m, 2H), 6.61 - 6.56 (m, 4H), 5.73 - 5.68 (m, 2H), 2.24 (s, 3H), 2.09 (s, 3H), 2.03 (s, 3H) ppm.

^{13}C{^{1}H}-NMR (151 MHz, DMSO-*d$_6$*): δ = 152.3, 143.4, 137.5, 136.8, 132.9, 126.4, 123.7, 120.8, 119.9, 116.0, 114.9, 112.2, 20.6, 15.2, 14.3 ppm.

The NMR data are in accordance with those reported in the literature.[S7]

3,4,5-Trimethyl-2-(10*H*-phenothiazin-10-yl)phenol (3an)

Compound **3an** was synthesized from 10*H*-phenothiazine **1a** (99 mg, 0.5 mmol, 1.0 equiv.) and 3,4,5-trimethyl phenol **2n** (205 mg, 3.0 mmol, 3.0 equiv.). The product was obtained as colorless solid (120 mg, 0.36 mmol, 72%) after column chromatography using *n*-hexane:dichloromethane in a 6:4 ratio.

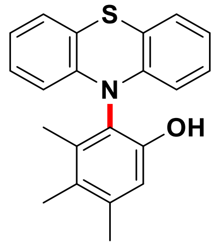

^{1}H-NMR (600 MHz, DMSO-*d$_6$*): δ = 9.39 (s, 1H), 6.94 (dd, *J* = 7.5, 1.6 Hz, 2H), 6.84 (ddd, *J* = 8.5, 7.4, 1.6 Hz, 2H), 6.76 (m, 1H), 6.75 (td, *J* = 7.5, 1.3 Hz, 2H), 5.97 (dd, *J* = 8.3, 1.3 Hz, 2H), 2.25 (s, 3H), 2.09 (s, 6H) ppm.

^{13}C{^{1}H}-NMR (151 MHz, DMSO-*d$_6$*): δ = 152.7, 142.1, 137.3, 135.9, 127.3, 126.7, 126.1, 123.1, 122.0, 118.2, 115.5, 115.0, 20.6, 15.3, 14.2 ppm.

The NMR data are in accordance with those reported in the literature.[S7]

3,4,5-Trimethyl-2-(10*H*-phenoselenazin-10-yl)phenol (3in)

Compound **3in** was synthesized from 10*H*-phenoselenazine **1i** (123 mg, 0.5 mmol, 1.0 equiv.) and 3,4,5-trimethyl phenol **2n** (204 mg, 3.0 mmol, 3.0 equiv.). The product was obtained as pale-yellow solid (136 mg, 0.36 mmol, 71%) after column chromatography using *n*-hexane:ethyl acetate in a 10:1 ratio, followed by column chromatography using 100% toluene.

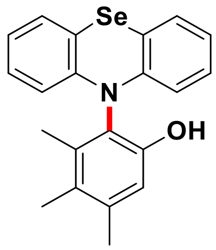

^{1}H-NMR (400 MHz, DMSO-*d*$_6$): δ = 9.41 (s, 1H), 7.15 (dd, *J* = 7.6, 1.6 Hz, 2H), 6.90 (ddd, *J* = 8.6, 7.2, 1.6 Hz, 2H), 6.82 - 6.74 (m, 3H), 6.15 (d, *J* = 8.4 Hz, 2H), 2.25 (s, 3H), 2.08 (s, 6H) ppm.

^{13}C{^{1}H}-NMR (101 MHz, DMSO-*d*$_6$): δ = 153.0, 142.4, 137.1, 135.6, 128.8, 127.5, 126.7, 124.8, 122.5, 116.6, 115.3, 113.9, 20.5, 15.3, 14.3 ppm.

^{77}Se-NMR (76 MHz, DMSO-*d*$_6$): δ = 239.17 (t, *J* = 9.3 Hz) ppm.

The NMR data are in accordance with those reported in the literature.[S2]

3,5-Dimethyl-2-(10*H*-phenothiazin-10-yl)phenol (*o*-3ao) and 3,5-dimethyl-4-(10*H*-phenothiazin-10-yl)phenol (*p*-3ao)

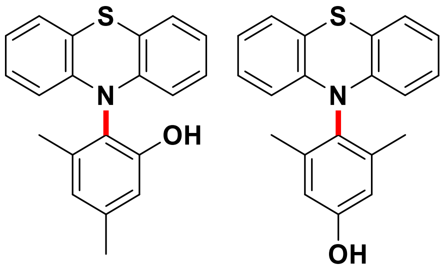

***o*-3ao** ***p*-3ao**

Compounds ***o*-3ao** and ***p*-3ao** were synthesized according to GP1 from 10*H*-phenothiazine **1a** (100 mg, 0.5 mmol, 1.0 equiv.) and 3,5-dimethylphenol **2o** (183 mg, 1.5 mmol, 3.0 equiv.). The products were obtained as colorless to beige solids (colorless, 35 mg, 0.11 mmol, 22% *ortho*-product; beige, 77 mg, 0.24 mmol, 48% *para*-product; 70% total yield, corresponding to a *p/o* selectivity of 2:1) after column chromatography using toluene:DCM in a 3:1 ratio.

ortho-Product ***o*-3ao**:

^{1}H-NMR (600 MHz DMSO-*d*$_6$): δ = 9.65 (s, 1H), 6.94 (dd, *J* = 7.6, 1.6 Hz, 2H), 6.86 (ddd, *J* = 8.4, 7.3, 1.6 Hz, 2H), 6.78 - 6.69 (m, 4H), 6.00 (dd, *J* = 8.3, 1.3 Hz, 2H), 2.28 (s, 3H), 2.07 (s, 3H) ppm.

^{13}C{^{1}H}-NMR (151 MHz, DMSO-*d*$_6$): δ = 155.3, 141.8, 138.9, 137.6, 127.3, 126.1, 122.8, 122.7, 122.1, 118.1, 115.0, 114.7, 21.0, 17.0 ppm.

NMR-data is in accordance with the literature.[S7]

para-Product ***p*-3ao**:

^{1}H-NMR (600 MHz DMSO-*d*$_6$): δ = 9.60 (s, 1H), 6.92 (dd, *J* = 7.6, 1.6 Hz, 2H), 6.84 (ddd, *J* = 8.5, 7.4, 1.6 Hz, 2H), 6.74 (td, *J* = 7.4, 1.2 Hz, 2H), 6.71 (s, 2H), 5.85 (dd, *J* = 8.3, 1.2 Hz, 2H), 2.02 (s, 6H) ppm.

^{13}C{^{1}H}-NMR (151 MHz, DMSO-d_6): δ = 157.0, 141.2, 138.5, 128.0, 127.6, 126.3, 122.2, 117.3, 116.0, 113.8, 17.6 ppm.

The NMR data are in accordance with those reported in the literature.[S7]

6-Isopropyl-3-methyl-2-(10*H*-phenothiazin-10-yl)phenol (*o*-3ap) and 2-isopropyl-5-methyl-4-(10*H*-phenothiazin-10-yl)phenol (*p*-3ap)

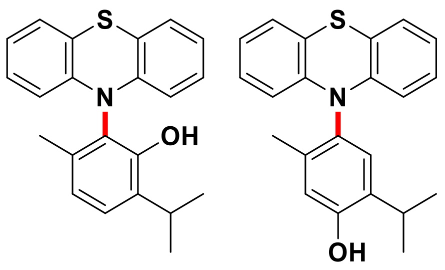

***o*-3ap** ***p*-3ap**

Compounds ***o*-3ap** and ***p*-3ap** were synthesized according to GP1 from 10*H*-phenothiazine **1a** (100 mg, 0.5 mmol, 1.0 equiv.) and thymol **2p** (225 mg, 1.5 mmol, 3.0 equiv.). The products were obtained as colorless solid and colorless oil (colorless solid, 7 mg, 0.02 mmol, 4% *ortho*-product; colorless oil, 135 mg, 0.39 mmol, 78% *para*-product; 82% total yield, corresponding to a *p/o* selectivity of 19:1) after column chromatography using hexane:DCM in a 6:4 ratio.

ortho-Product ***o*-3ap**:

^{1}H-NMR (600 MHz DMSO-d_6): δ = 8.97 (s, 1H), 7.19 (d, J = 7.8 Hz, 1H), 6.91 (dd, J = 7.6, 1.6 Hz, 2H), 6.87 (d, J = 7.9 Hz, 1H), 6.84 (ddd, J = 8.4, 7.3, 1.4 Hz, 2H), 6.74 (td, J = 7.4, 1.1 Hz, 2H), 5.90 (dd, J = 8.3, 1.2 Hz, 2H), 3.28 (overlapping in part with residual water peak, *sept*, J = 6.6 Hz, 1H), 2.08 (s, 3H), 1.21 (d, J = 6.8 Hz, 6H) ppm.

^{13}C{^{1}H}-NMR (151 MHz, DMSO-d_6): δ = 152.7, 141.3, 134.2, 134.0, 127.3, 126.0, 125.9, 125.8, 122.1, 121.9, 118.5, 114.8, 26.3, 22.7, 17.0 ppm.

HRMS (ESI, m/z): calculated for $C_{22}H_{21}NOS^+$ (M^+): 347.1338, found: 347.1342.

IR (neat, cm^{-1}): 3484, 3227, 3064, 2959, 2925, 2865, 2118, 1889, 1724, 1592, 1460, 1381, 1307, 1241, 1200, 1144, 1040, 921, 853, 813, 744, 661.

para-Product ***p*-3ap**:

^{1}H-NMR (600 MHz DMSO-d_6): δ = 9.64 (s, 1H), 6.98 (s, 1H), 6.96 (dd, J = 7.5, 1.6 Hz, 2H), 6.89 (s, 1H), 6.87 (td, J = 8.4, 1.5 Hz, 2H), 6.77 (td, J = 7.5, 1.2 Hz, 2H), 5.98 (dd, J = 8.3, 1.3 Hz, 2H), 3.20 (*sept*, J = 6.9 Hz, 1H), 1.96 (s, 3H), 1.16 (d, J = 6.9 Hz, 6H) ppm.

^{13}C{^{1}H}-NMR (151 MHz, DMSO-d_6): δ = 154.4, 142.9, 135.1, 134.7, 129.2, 127.9, 127.4, 126.4, 122.2, 117.9, 117.9, 114.7, 26.3, 22.4, 17.0 ppm.

The NMR data are in accordance with those reported in the literature.[S11]

2,3-Dimethyl-6-(10*H*-phenothiazin-10-yl)phenol (*o*-3aq) and 2,3-dimethyl-4-(10*H*-phenothiazin-10-yl)phenol (*p*-3aq)

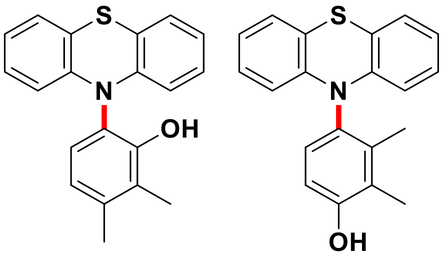

***o*-3aq** ***p*-3aq**

Compounds ***o*-3aq** and ***p*-3aq** were synthesized according to GP1 from 10*H*-phenothiazine **1a** (99 mg, 0.5 mmol, 1.0 equiv.) and 2,3-dimethylphenol **2q** (183 mg, 1.5 mmol, 3.0 equiv.). The products were obtained as pale-green solid (6 mg, 0.02 mmol, 4%, *ortho*-product) and as beige solid (153 mg, 0.48 mmol, 96%, *para*-product) after column chromatography using hexane:ethyl acetate in a 10:1 ratio.

ortho-Product ***o*-3aq**:

^{1}**H-NMR** (600 MHz DMSO-d_6): δ = 8.93 (s, 1H), 6.94 - 6.91 (m, 3H), 6.87 – 6.82 (m, 3H), 6.75 (td, *J* = 7.4, 1.3 Hz, 2H), 5.98 (dd, *J* = 8.3, 1.2 Hz, 2H), 2.29 (s, 3H), 2.18 (s, 3H) ppm.

^{13}**C{^{1}H}-NMR** (151 MHz, DMSO-d_6): δ = 153.1, 142.8, 138.4, 127.3, 127.2, 126.0, 125.1, 124.9, 122.6, 122.0, 118.3, 115.4, 20.1, 12.5 ppm.

HRMS (EI, m/z): calculated for $C_{20}H_{17}NOS^+$ (M^+): 319.1025, found: 319.1026.

IR (in $CHCl_3$, cm^{-1}): 3480, 3063, 2924, 2857, 2116, 2005, 1893, 1723, 1584, 1461, 1383, 1307, 1231, 1086, 1042, 1015, 901, 809, 748, 664.

para-Product ***p*-3aq**:

^{1}**H-NMR** (600 MHz DMSO-d_6): δ = 9.67 (s, 1H), 6.98 - 6.95 (m, 3H), 6.90 (d, *J* = 8.4 Hz, 1H), 6.86 (td, *J* = 7.8, 1.7 Hz, 2H), 6.77 (t, *J* = 7.5 Hz, 2H), 5.98 (d, *J* = 8.3 Hz, 2H), 2.14 (s, 3H), 1.99 (s, 3H) ppm.

^{13}**C{^{1}H}-NMR** (151 MHz, DMSO-d_6): δ = 155.1, 143.0, 137.2, 129.4, 128.2, 127.4, 126.3, 124.9, 122.2, 118.0, 115.0, 114.2, 14.4, 12.3 ppm.

The NMR data are in accordance with those reported in the literature.[S10]

5-Isopropyl-2-methyl-4-(10*H*-phenothiazin-10-yl)phenol (3ar)

Compound **3ar** was synthesized according to GP1 from 10*H*-phenothiazine **1a** (100 mg, 0.5 mmol, 1.0 equiv.) and carvacrol **2r** (230 μL, 1.5 mmol, 3.0 equiv.). The product was obtained as pale-yellow foam (167 mg, 0.48 mmol, 96%) after column chromatography using toluene:DCM in a 3:1 ratio.

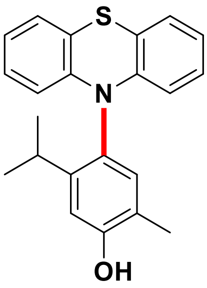

^{1}H-NMR (600 MHz, DMSO-*d*$_6$): δ = 9.62 (s, 1H), 6.99 (s, 1H), 6.96 (dd, *J* = 7.6, 1.6 Hz, 2H), 6.94 (s, 1H), 6.87 (ddd, *J* = 8.5, 7.3, 1.6 Hz, 2H), 6.77 (td, *J* = 7.4, 1.2 Hz, 2H), 6.07 (dd, *J* = 8.3, 1.2 Hz, 2H), 3.05 (*sept*, *J* = 6.9 Hz, 1H), 2.16 (s, 3H), 0.98 (d, *J* = 6.8 Hz, 6H) ppm.

^{13}C{^{1}H}-NMR (151 MHz, DMSO-*d*$_6$): δ = 155.8, 145.3, 143.5, 132.9, 127.6, 127.1, 126.3, 124.0, 122.2, 117.8, 115.3, 113.3, 27.3, 23.5, 15.6 ppm.

The NMR data are in accordance with those reported in the literature.[S10]

2-Methyl-4-(10*H*-phenothiazin-10-yl)phenol (3as)

Compound **3as** was synthesized according to GP1 from 10*H*-phenothiazine **1a** (99 mg, 0.5 mmol, 1.0 equiv.) and *o*-cresol **2s** (155 µL, 1.5 mmol, 3.0 equiv.). The product was obtained as colorless solid (87 mg, 0.29 mmol, 57%) after column chromatography using *n*-hexane:DCM in a 6:4 ratio.

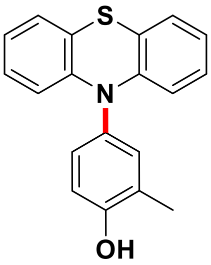

^{1}H-NMR (600 MHz, DMSO-*d*$_6$): δ = 9.77 (s, 1H), 7.09 (s, 1H), 7.03 (d, *J* = 8.5 Hz, 1H), 7.01 (d, *J* = 8.5 Hz, 1H), 7.00 (dd, *J* = 7.6, 1.6 Hz, 2H), 6.90 (ddd, *J* = 8.5, 7.3, 1.6 Hz, 2H), 6.80 (td, *J* = 7.4, 1.2 Hz, 2H), 6.18 (dd, *J* = 8.3, 1.2 Hz, 2H), 2.18 (s, 3H) ppm.

^{13}C{^{1}H}-NMR (151 MHz, DMSO-*d*$_6$): δ = 155.4, 144.2, 132.4, 130.6, 128.8, 127.2, 126.7, 126.4, 122.3, 118.5, 116.4, 115.6, 16.0 ppm.

HRMS (EI, m/z): calculated for $C_{19}H_{15}ONS^+$ (M^+): 305.0869, found: 305.0865.

IR (neat, cm^{-1}): 3545, 3059, 2922, 2855, 2327, 2186, 2112, 1991, 1934, 1892, 1710, 1590, 1568, 1497, 1453, 1298, 1236, 1169, 1105, 1040, 1000, 969, 924, 821, 741.

2-Methoxy-4-(10*H*-phenothiazin-10-yl)phenol (3at)

Compound **3at** was synthesized according to GP1 from 10*H*-phenothiazine **1a** (99 mg, 0.5 mmol, 1.0 equiv.) and guaiacol **2t** (2-methoxyphenol, 165 µL, 1.5 mmol, 3.0 equiv.). The product was obtained as pale-green solid (68 mg, 0.21 mmol, 42%) after column chromatography using *n*-hexane:ethyl acetate in a 5:1 ratio. Compound **3at** was also synthetized as described above, but with 2 h of milling time instead of 1 h. The product was

obtained as pale-green solid (83 mg, 0.26 mmol, 52%) after column chromatography using *n*-hexane:ethyl acetate in a 5:1 ratio.

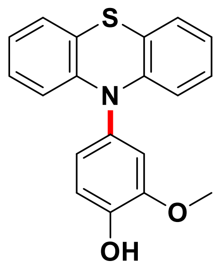

^{1}H-NMR (600 MHz, DMSO-d_6): δ = 9.45 (s, 1H), 7.04 - 6.99 (m, 3H), 6.93 – 6.88 (m, 3H), 6.81 (≈t, J = 7.4 Hz, 3H), 6.22 (d, J = 8.3 Hz, 2H), 3.77 (s, 3H) ppm.

^{13}C{^{1}H}-NMR (151 MHz, DMSO-d_6): δ = 149.4, 146.7, 144.1, 131.0, 127.3, 126.4, 123.0, 122.3, 118.4, 116.7, 115.6, 114.0, 55.8 ppm.

The NMR data are in accordance with those reported in the literature.[S10]

10-(2-Phenyl-1*H*-indol-3-yl)-10*H*-phenothiazine (3au)

Compound **3au** was synthesized according to GP1 from 10*H*-phenothiazine **1a** (100 mg, 0.5 mmol, 1.0 equiv.) and 2-phenylindole **2u** (290 mg, 1.5 mmol, 3.0 equiv.). The product was obtained as beige solid (77 mg, 0.20 mmol, 39%) after column chromatography using *n*-hexane:ethyl acetate in a 10:1 ratio.

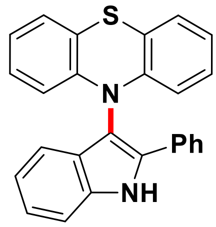

^{1}H-NMR (600 MHz, DMSO-d_6): δ = 11.93 (s, 1H), 7.93 (dd, J = 8.5, 1.3 Hz, 2H), 7.58 (d, J = 8.2 Hz, 1H), 7.43 (t, J = 7.9 Hz, 2H), 7.31 (tt, J = 7.4, 1.3 Hz, 1H), 7.27 - 7.22 (m, 2H), 7.11 – 7.04 (m, 3H), 6.87 - 6.81 (m, 4H), 6.29 (dd, J = 7.8, 1.7 Hz, 2H) ppm.

^{13}C{^{1}H}-NMR (151 MHz DMSO-d_6): δ = 143.5, 135.1, 133.0, 130.5, 128.9, 128.2, 127.5, 126.7, 126.2, 125.8, 122.8, 122.8, 120.3, 119.6, 118.0, 115.7, 112.7, 112.5 ppm.

The NMR data are in accordance with those reported in the literature.[S10]

4-(10*H*-Phenothiazin-10-yl)-*N*-phenylnaphthalen-1-amine (3av)

Compound **3av** was synthesized according to GP1 from 10*H*-phenothiazine **1a** (99 mg, 0.5 mmol, 1.0 equiv.) and *N*-phenylnaphthalen-1-amine **2v** (328 mg, 1.5 mmol, 3.0 equiv.). The product was obtained as pale-green foam (119 mg, 0.28 mmol, 57%) after column chromatography using *n*-hexane:dichloromethane in a 6:1 ratio.

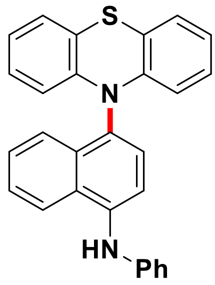

^{1}H-NMR (600 MHz, $CDCl_3$): δ = 8.14 (d, J = 8.6 Hz, 1H), 8.12 (d, J = 8.5 Hz, 1H), 7.55 (t, J = 7.0 Hz, 1H), 7.52 - 7.47 (m, 3H), 7.38 (t, J = 7.9 Hz, 2H), 7.21 (d, J = 7.4 Hz, 2H), 7.06 (t, J = 7.4 Hz, 1H), 7.04 (dd, J = 7.4, 1.8 Hz, 2H), 6.79 (t, J = 7.3 Hz, 2H), 6.75 (td, J = 7.7, 1.7 Hz, 2H), 6.20 (br s, NH), 6.15 (dd, J = 8.1, 1.4 Hz, 2H) ppm.

^{13}C{^{1}H}-NMR (151 MHz, $CDCl_3$): δ = 144.2, 143.4, 140.1, 132.5, 130.8, 129.8, 129.7, 128.2, 127.6, 127.0, 126.6, 126.5, 124.5, 122.4, 122.1, 121.9, 119.7, 119.2, 115.9, 113.3 ppm.

The NMR data are in accordance with those reported in the literature.[S12]

1-(10*H*-Phenothiazin-10-yl)-*N*-phenylnaphthalen-2-amine (3aw)

Compound **3aw** was synthesized according to GP1 from 10*H*-phenothiazine **1a** (99 mg, 0.5 mmol, 1.0 equiv.) and *N*-phenylnaphthalen-2-amine **2w** (329 mg, 1.5 mmol, 3.0 equiv.). The product was obtained as colorless foam (180 mg, 0.43 mmol, 86%) after column chromatography using *n*-hexane:dichloromethane in a 6:4 ratio.

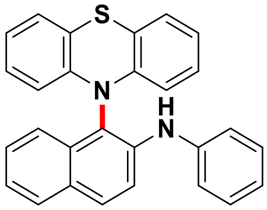

^{1}H-NMR (600 MHz, DMSO-d_6): δ = 8.03 (s, 1H), 7.93 (d, J = 9.1 Hz, 1H), 7.89 (dd, J = 8.1, 1.3 Hz, 2H), 7.61 (d, J = 9.1 Hz, 1H), 7.44 (ddd, J = 8.3, 6.8, 1.3 Hz, 1H), 7.34 - 7.26 (m, 3H), 7.21 (d, J = 7.4 Hz, 2H), 7.02 - 6.95 (m, 3H), 6.78 - 6.71 (m, 4H), 6.04 - 5.98 (m, 2H) ppm.

^{13}C{^{1}H}-NMR (151 MHz DMSO-d_6): δ = 142.0, 141.6, 141.4, 131.3, 129.6, 129.4, 129.1, 128.6, 127.8, 127.4, 126.2, 123.3, 122.4, 122.3, 121.1, 120.9, 119.0, 118.9, 117.7, 115.2 ppm.

The NMR data are in accordance with those reported in the literature.[S13]

5. Literature

[S1] C. Cremer, M. Goswami, C. K. Rank, B. de Bruin, and F. W. Patureau, “Tellurium(II)/Tellurium(III)-Catalyzed Cross-Dehydrogenative C–N Bond Formation,” *Angewandte Chemie International Edition* 60 (2021), 6451–6456. C. Cremer, M. Goswami, C. K. Rank, B. de Bruin, and F. W. Patureau, “Tellur(II)/Tellur(III)-katalysierte dehydrierende C–N Bindungsbildung,” *Angewandte Chemie* 133 (2021), 6525–6530.

[S2] C. Cremer, M. A. Eltester, H. Bourakhouadar, I. L. Atodiresei and F. W. Patureau, “Dehydrogenative C–H Phenochalcogenazination,” *Organic Letters* 23 (2021), 3243–3247.

[S3]: Y. Zhao, B. Huang, C. Yang, and W. Xia, "Visible-Light-Promoted Direct Amination of Phenols via Oxidative Cross-Dehydrogenative Coupling Reaction," *Organic Letters* 18 (2016): 3326–3329.

[S4]: R. A. Sheldon, "Organic Synthesis-Past, Present and Future," *Chemistry & Industry* (1992): 903–906.

[S5]: B. M. Trost, "The Atom Economy-A Search for Synthetic Efficiency," *Science* 254 (1991): 1471–1477.

[S6]: C. Jimenez-Gonzales, C. S. Ponder, Q. B. Broxterman, and J. B. Manley, "Using the Right Green Yardstick: Why Process Mass Intensity Is Used in the Pharmaceutical Industry To Drive More Sustainable Processes," *Organic Process Research & Development* 15 (2011): 912–917.

[S7]: M.-L. Louillat-Habermeyer, R. Jin, and F. W. Patureau, "O_2-mediated dehydrogenative amination of phenols," *Angewandte Chemie International Edition* 54 (2015): 4102–4104. M.-L. Louillat-Habermeyer, R. Jin, and F. W. Patureau, "O_2-vermittelte dehydrierende Aminierung von Phenolen," *Angewandte Chemie* 127 (2015): 4175–4177.

[S8] L. Bering, L. D'Ottavio, G. Sirvinskaite, and A. P. Antonchick, "Nitrosonium ion catalysis: aerobic, metal-free cross-dehydrogenative carbon–heterobond formation," *Chemical Communications* 54 (2018): 13022–13025.

[S9] S. Tang, S. Wang, Y. Liu, H. Cong, and A. Lei, "Electrochemical Oxidative C-H Amination of Phenols: Access to Triarylamine Derivatives," *Angewandte Chemie International Edition* 57 (2018): 4737–4741. S. Tang, S. Wang, Y. Liu, H. Cong, and A. Lei, "Electrochemical Oxidative C-H Amination of Phenols: Access to Triarylamine Derivatives," *Angewandte Chemie* 130 (2018): 4827–4831.

[S10] F. Xiao, X. Wang, B. Ebel, I. M. Oppel, and F. W. Patureau, "O_2-Mediated Cu-Catalyzed Dehydrogenative Phenothiazination," *The Journal of Organic Chemistry* 90 (2025): 1180–1185.

[S11] A. Purtsas, M. Rosenkranz, E. Dmitrieva, O. Kataeva and H.-J. Knölker, "Iron-Catalyzed Oxidative C−O and C−N Coupling Reactions Using Air as Sole Oxidant," *Chemistry – A European Journal* 28 (2022), e202104292.

[S12] P. Y. Vemuri, C. Cremer, and F. W. Patureau, "Te(II)-Catalyzed Cross-Dehydrogenative Phenothiazination of Anilines," *Organic Letters* 24 (2022): 1626–1630.

[S13] S. Chen, Y.-N. Li, S.-H. Xiang, S. Li, and B. Tan, "Electrochemical phenothiazination of naphthylamines and its application in photocatalysis," *Chemical Communications* 57 (2021), 8512–8515.

6. NMR Spectra

4-(*tert*-Butyl)-2-(10*H*-phenothiazin-10-yl)phenol (3aa)

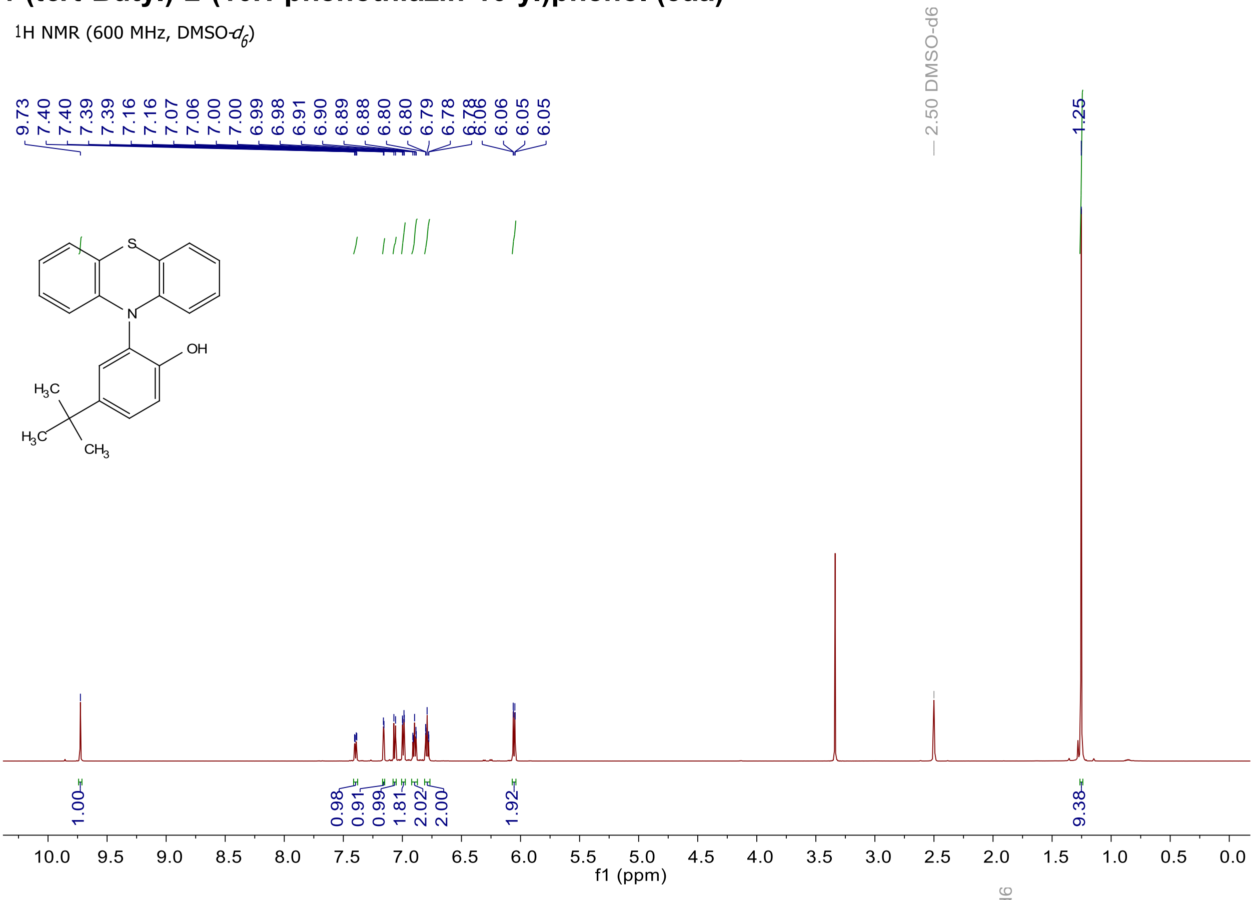

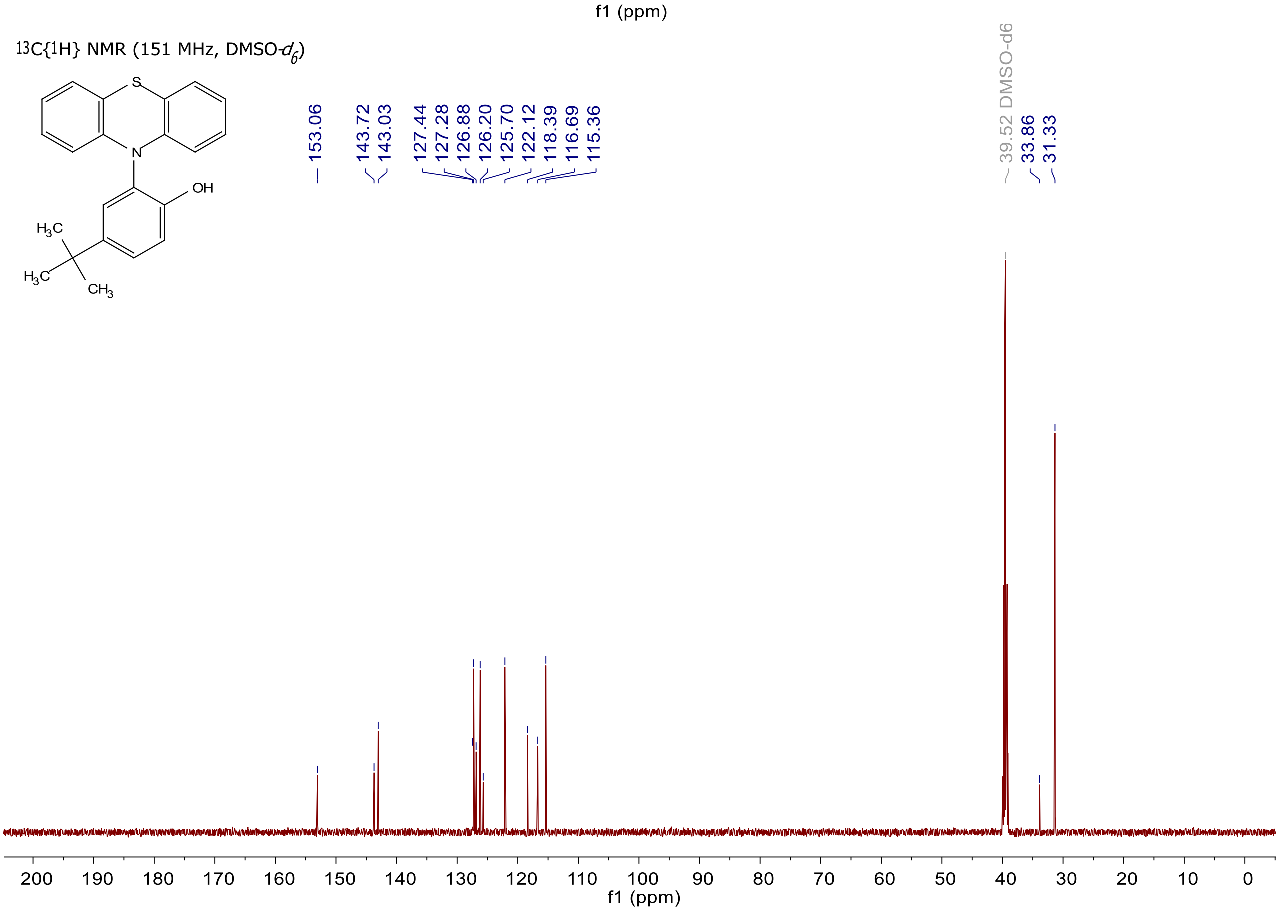

4-Methyl-2-(10*H*-phenothiazin-10-yl)phenol (3ab)

^{1}H NMR (600 MHz, DMSO-d_6)

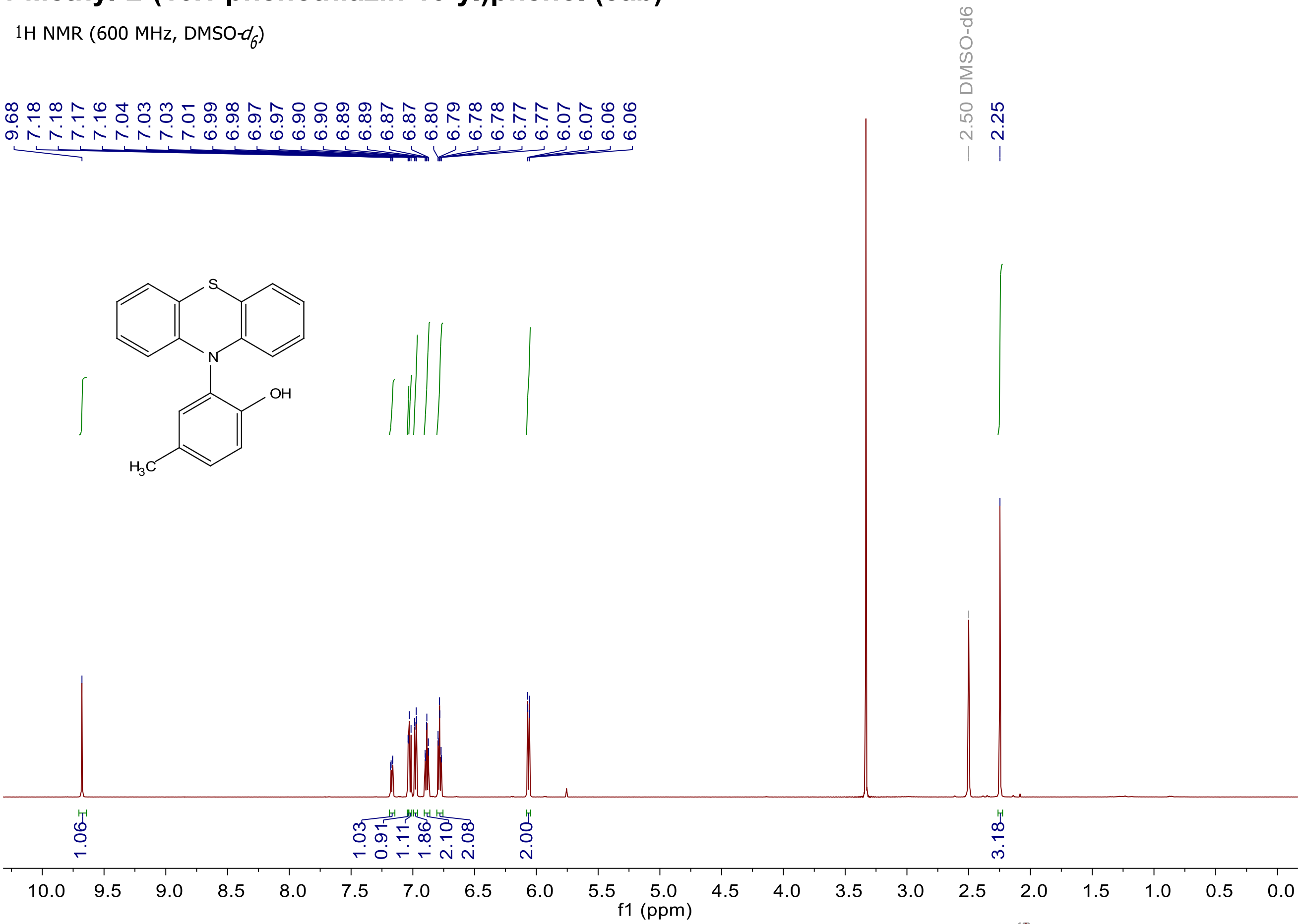

$^{13}C\{^{1}H\}$ NMR (151 MHz, DMSO-d_6)

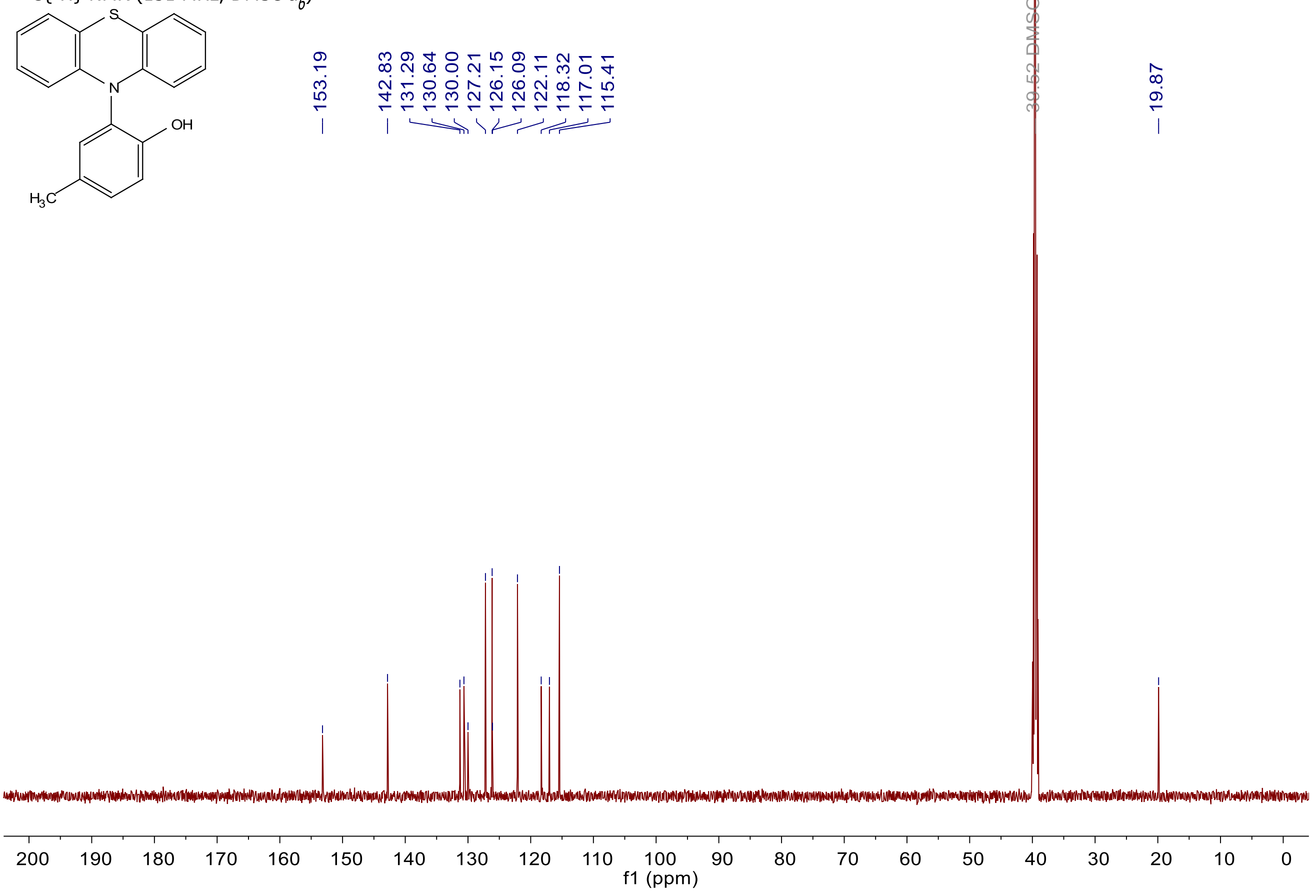

3-(10*H*-Phenothiazin-10-yl)-[1,1‘-biphenyl]-4-ol (3ac)

^{1}H NMR (400 MHz, DMSO-d_6)

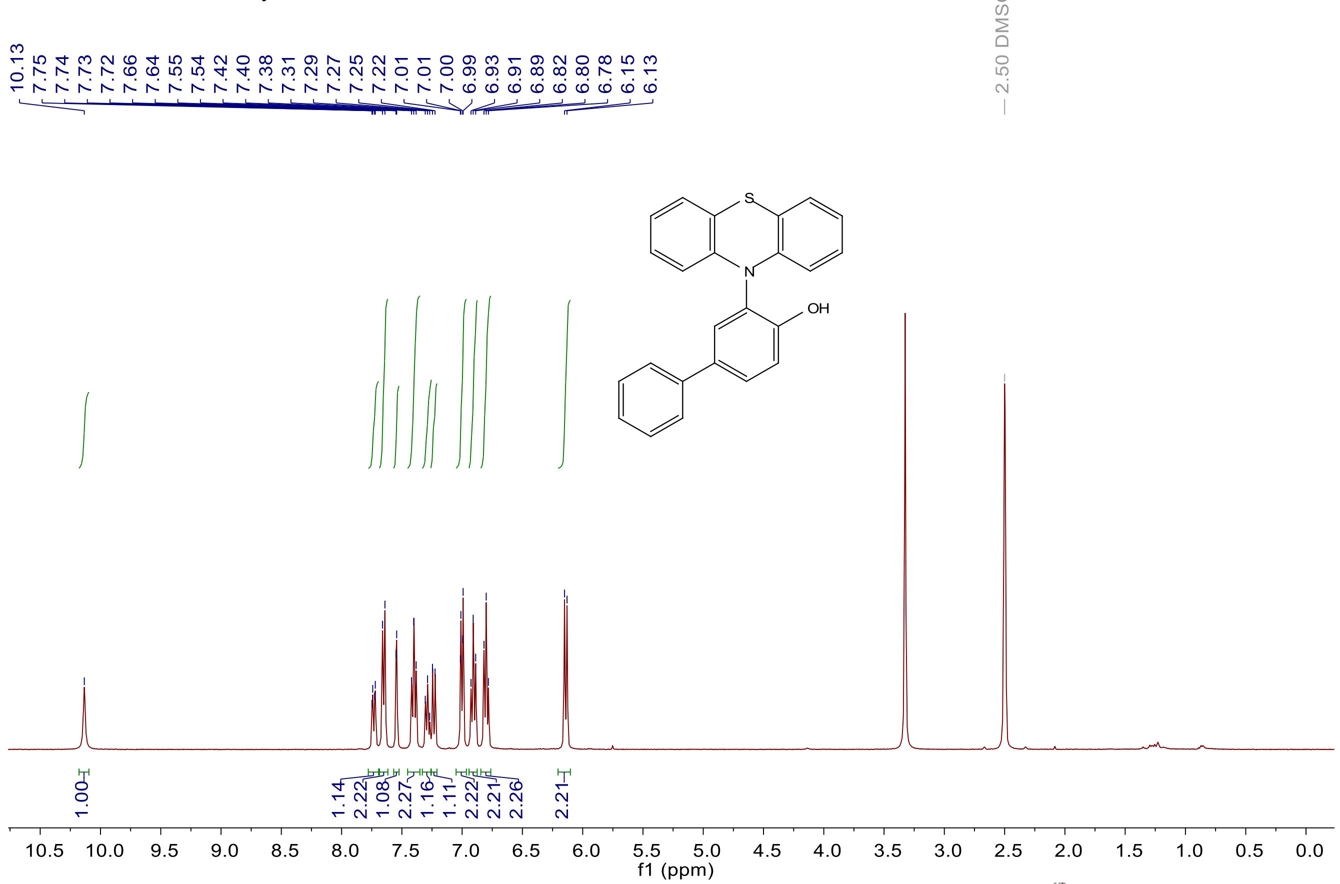

$^{13}C\{^{1}H\}$ NMR (151 MHz, DMSO-d_6)

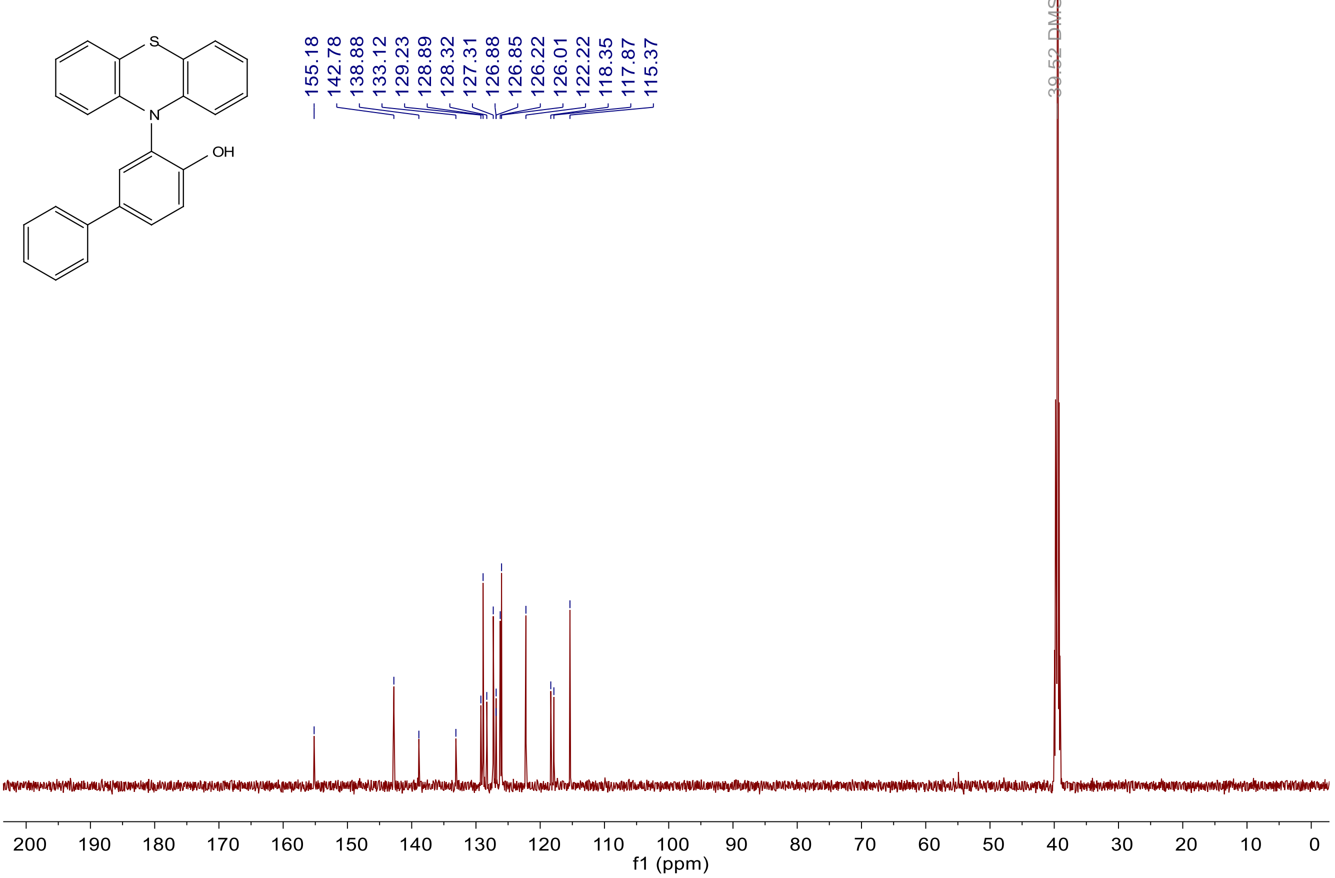

4-(Adamantan-1-yl)-2-(10*H*-phenothiazin-10-yl)phenol (3ad)

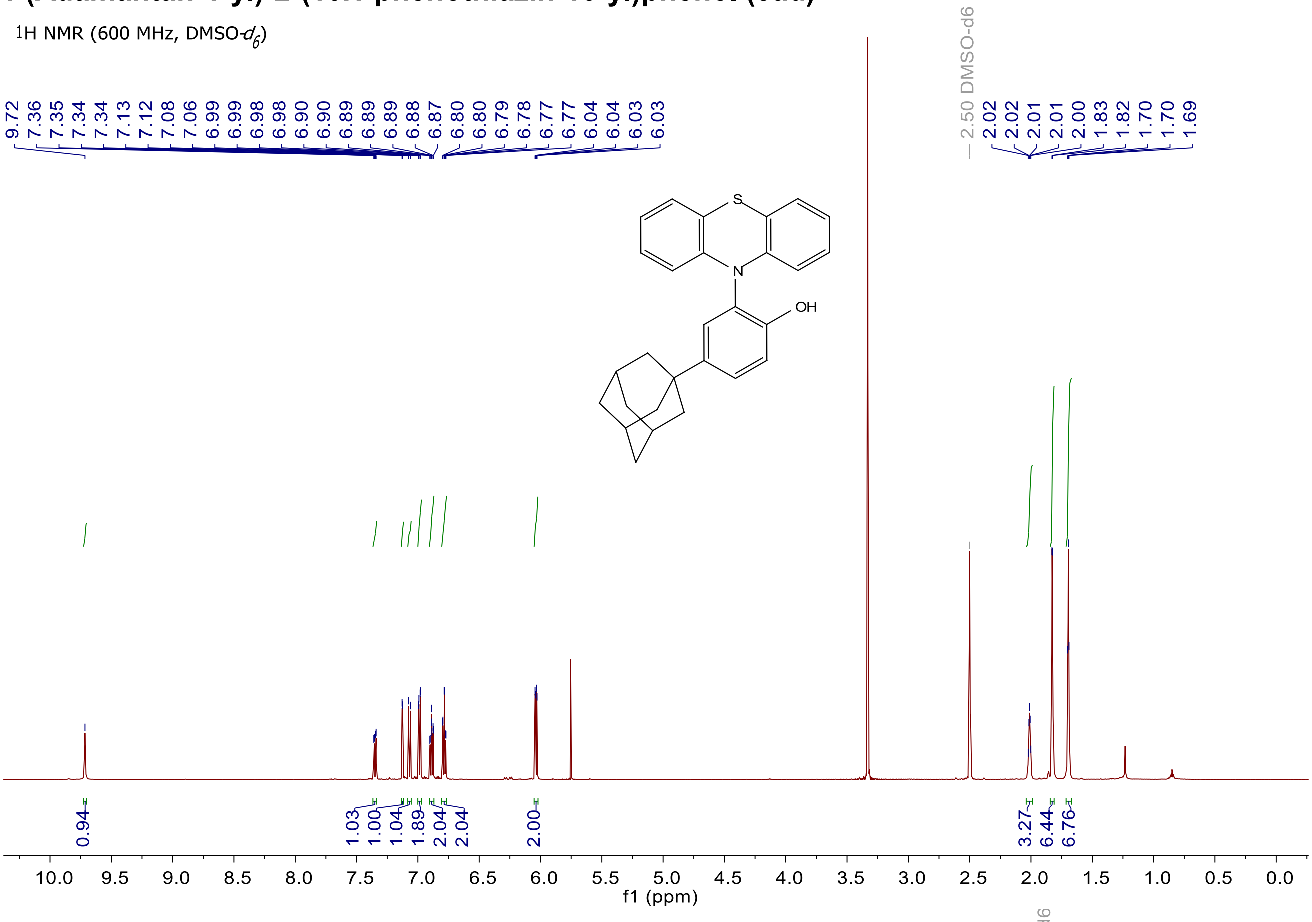

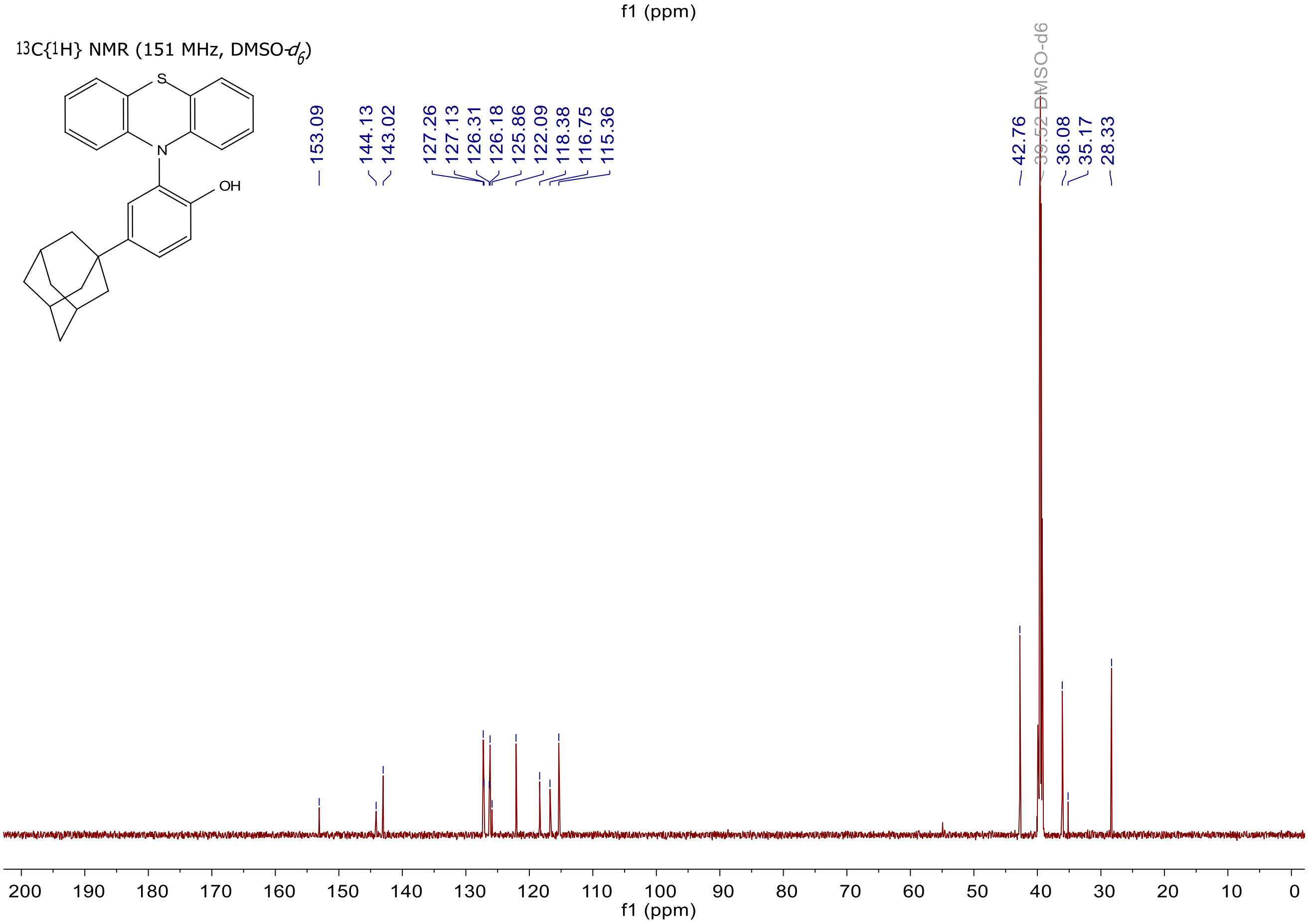

4-Methoxy-2-(10*H*-phenothiazin-10-yl)phenol (3ae)

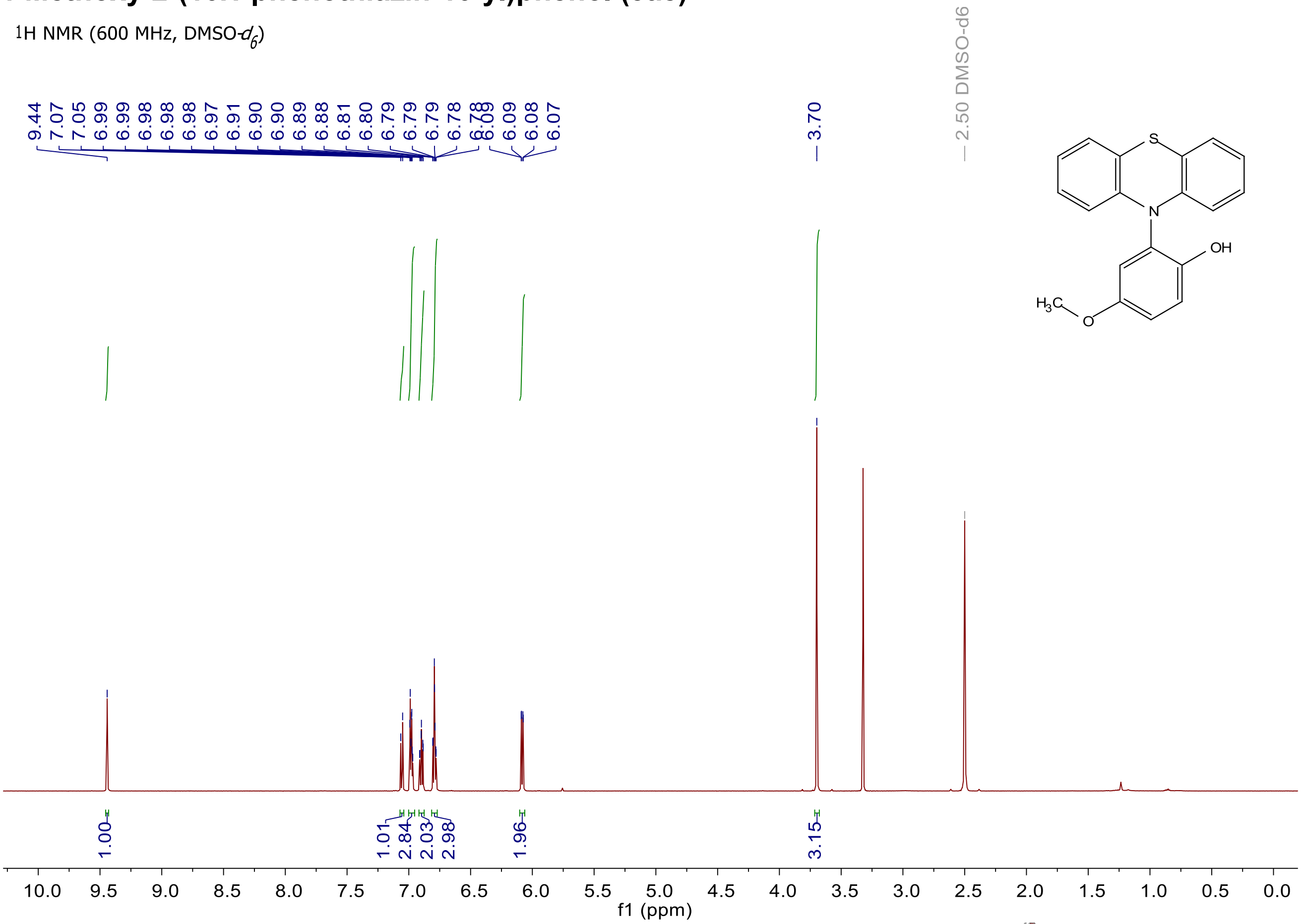

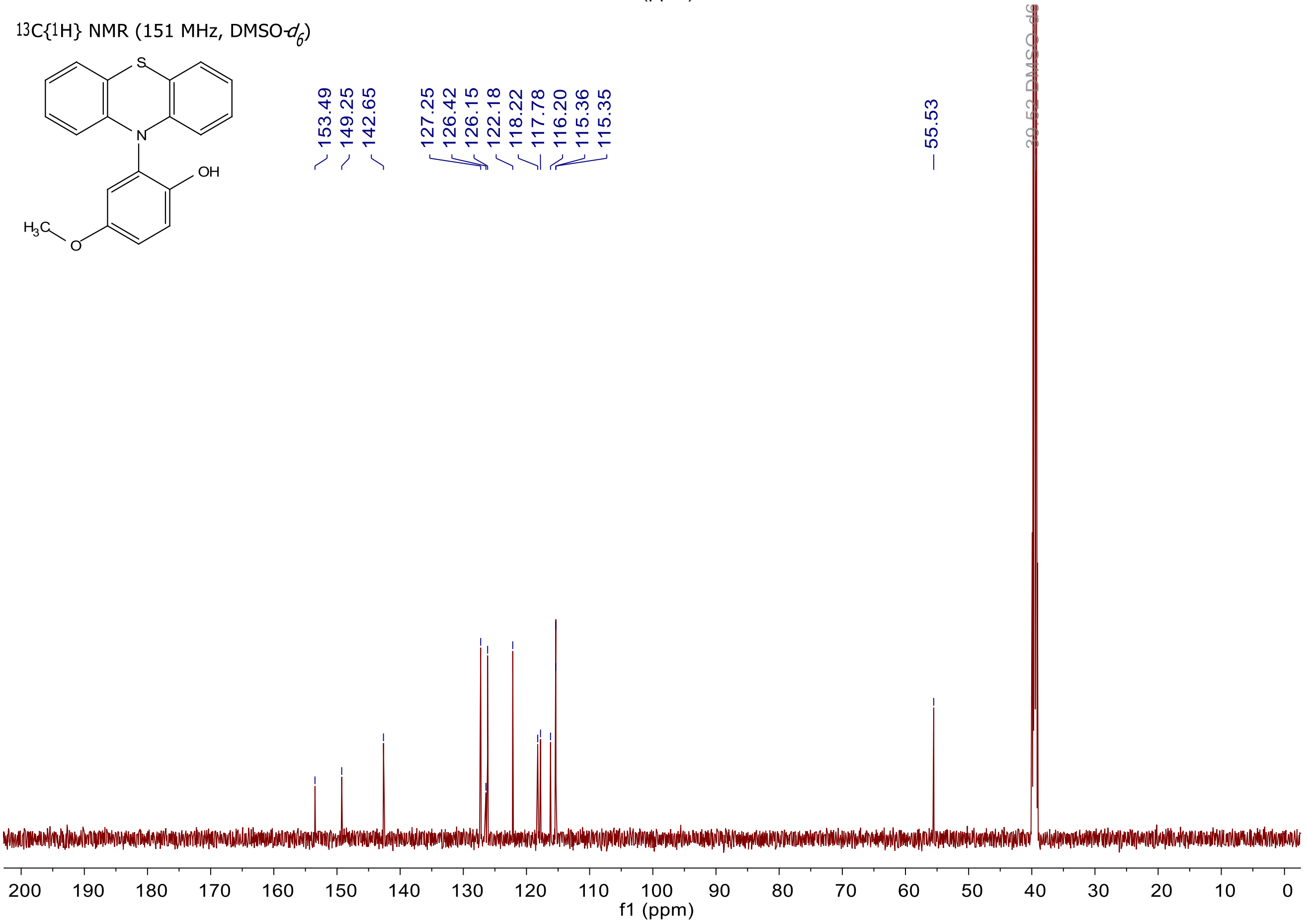

4-Methylthio-2-(10*H*-phenothiazin-10-yl)phenol (3af)

^{1}H NMR (600 MHz, DMSO-d_6)

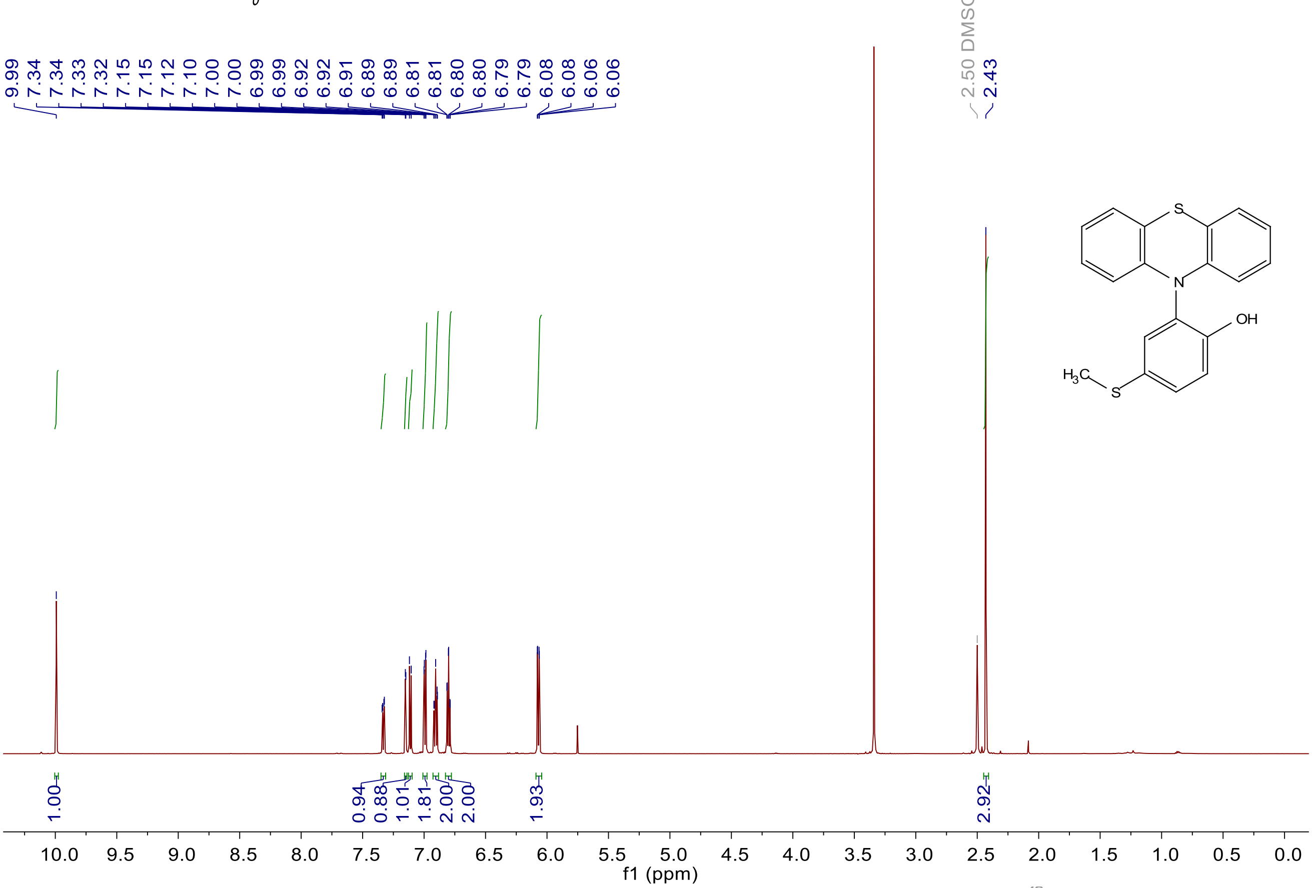

$^{13}C\{^{1}H\}$ NMR (151 MHz, DMSO-d_6)

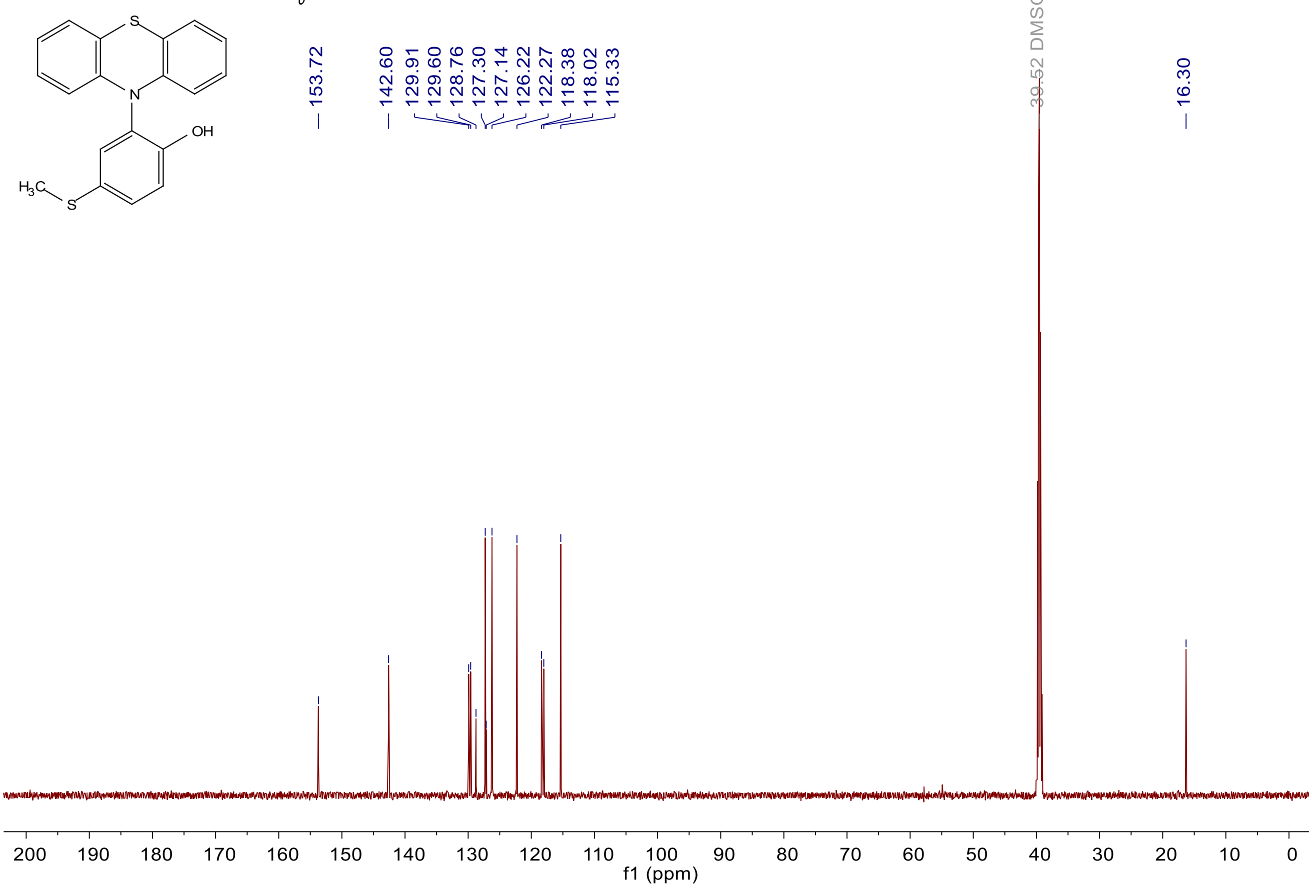

4-Bromo-2-(10*H*-phenothiazin-10-yl)phenol (3ag)

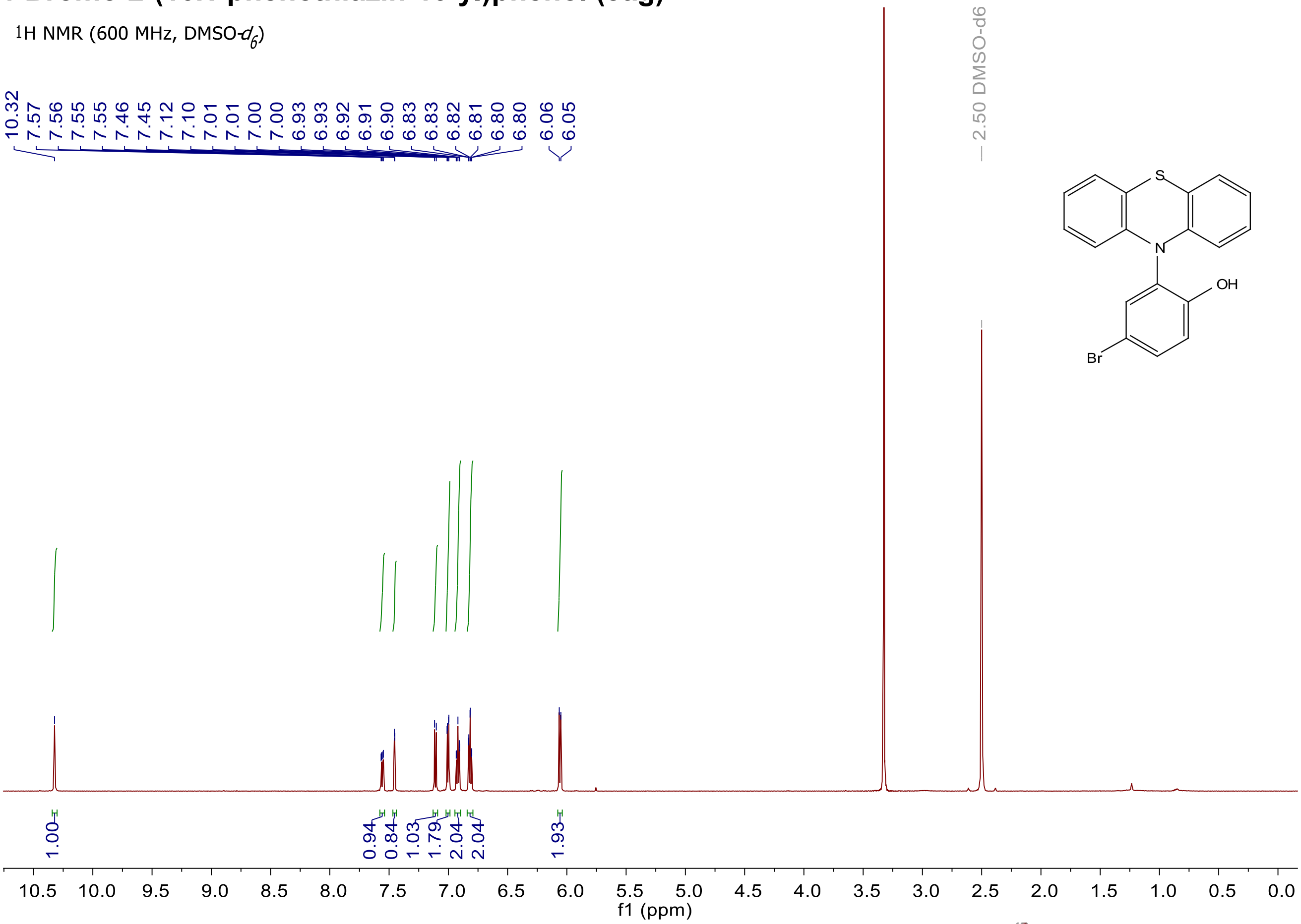

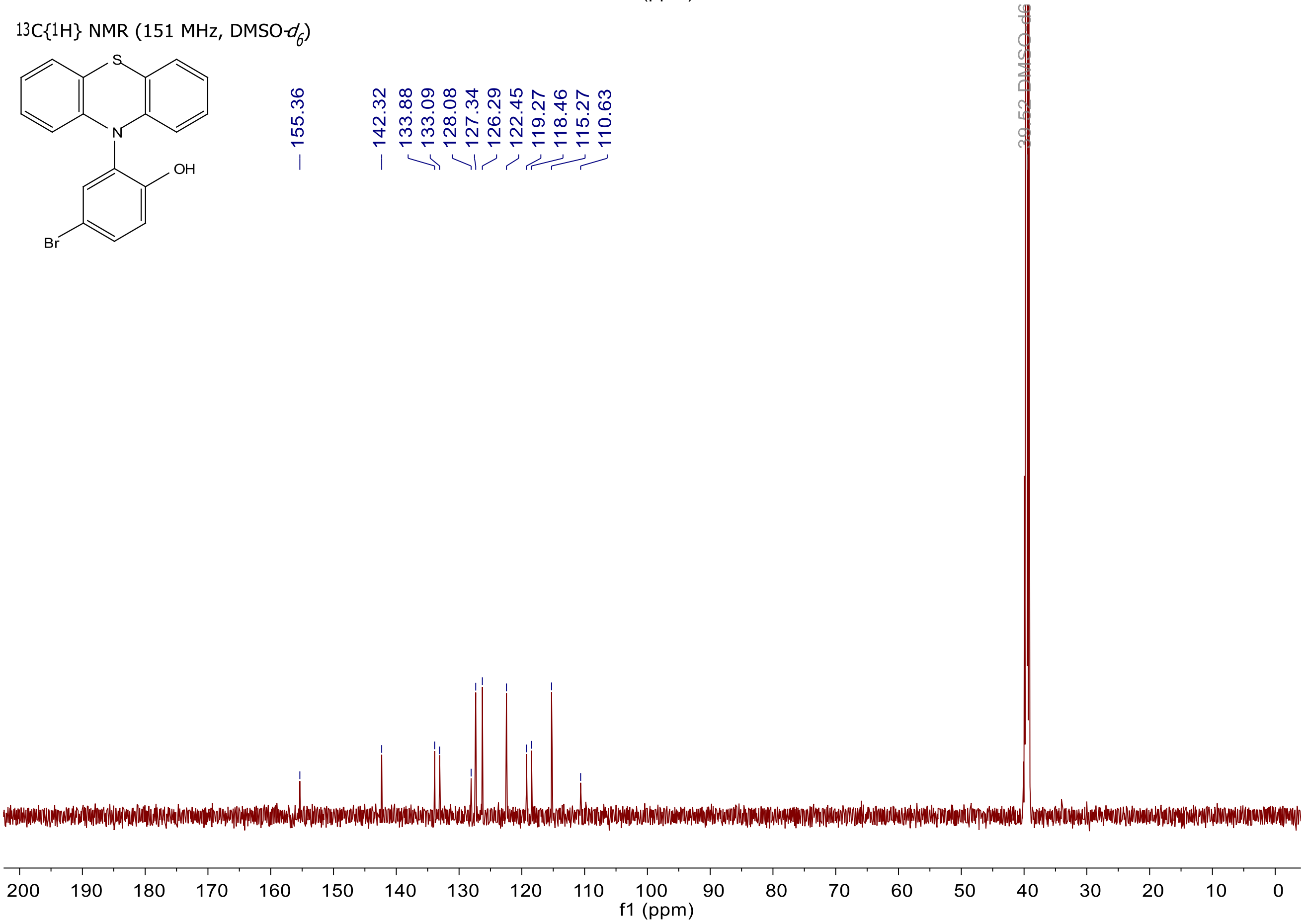

4-Chloro-2-(10*H*-phenothiazin-10-yl)phenol (3ah)

^{1}H NMR (600 MHz, DMSO-d_6)

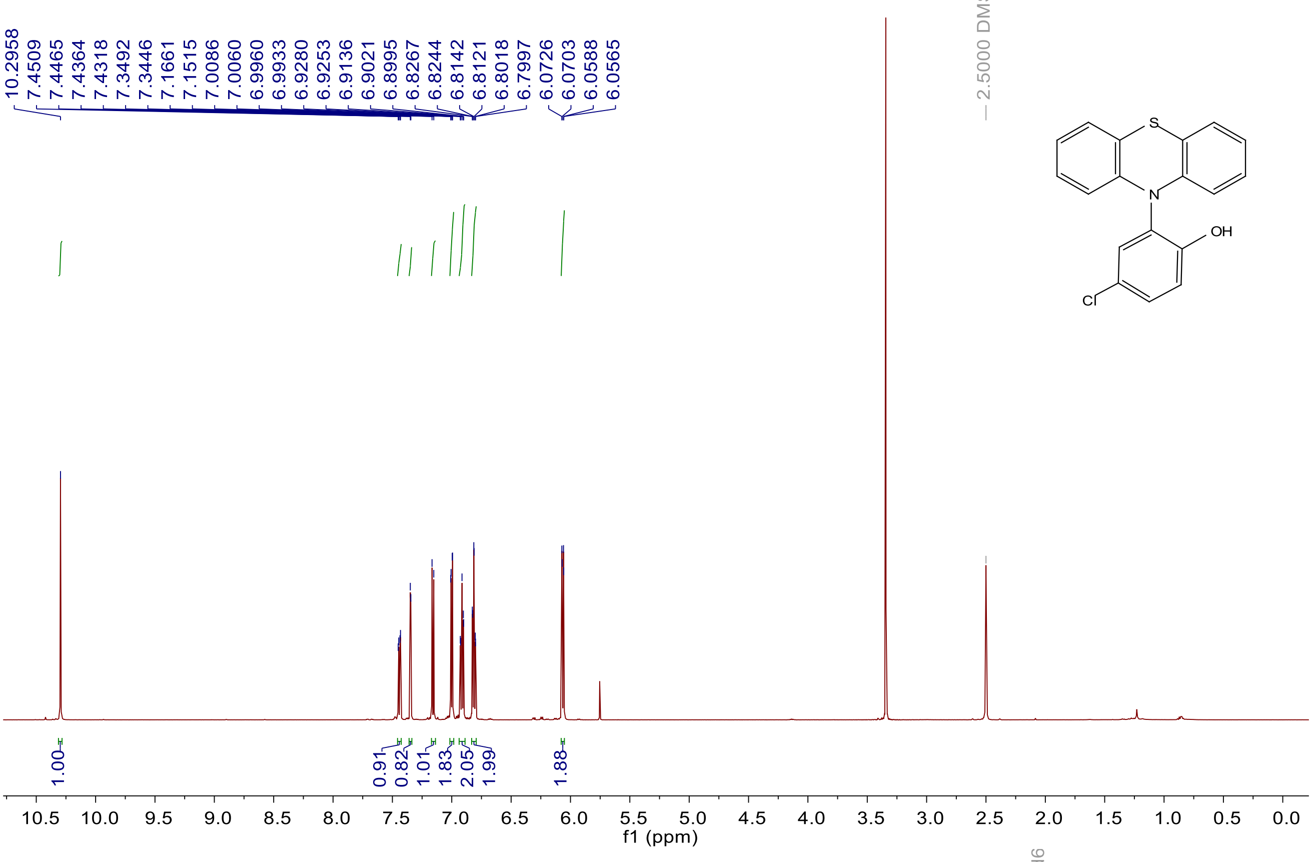

^{13}C{^{1}H} NMR (151 MHz, DMSO-d_6)

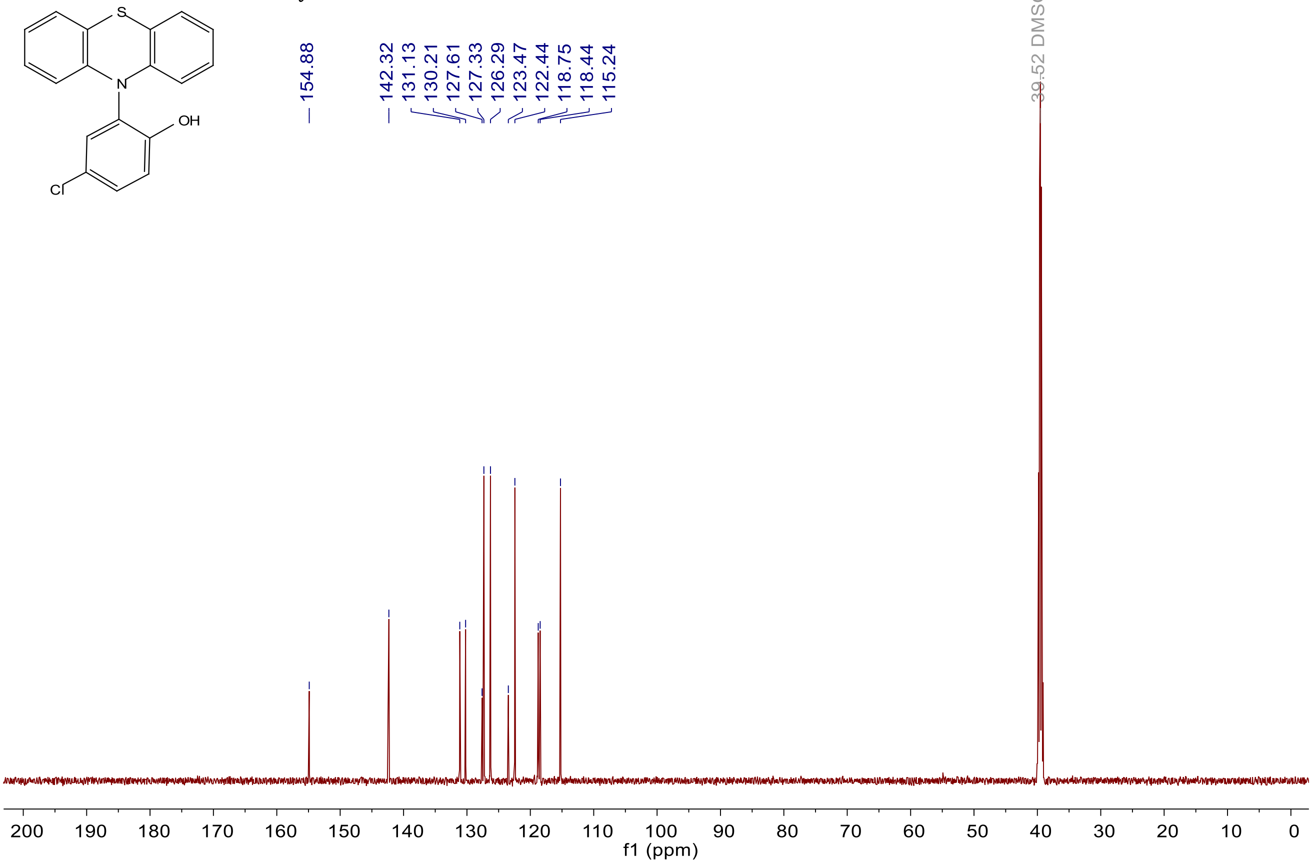

4-Fluoro-2-(10*H*-phenothiazin-10-yl)phenol (3ai)

^{1}H NMR (600 MHz, DMSO-d_6)

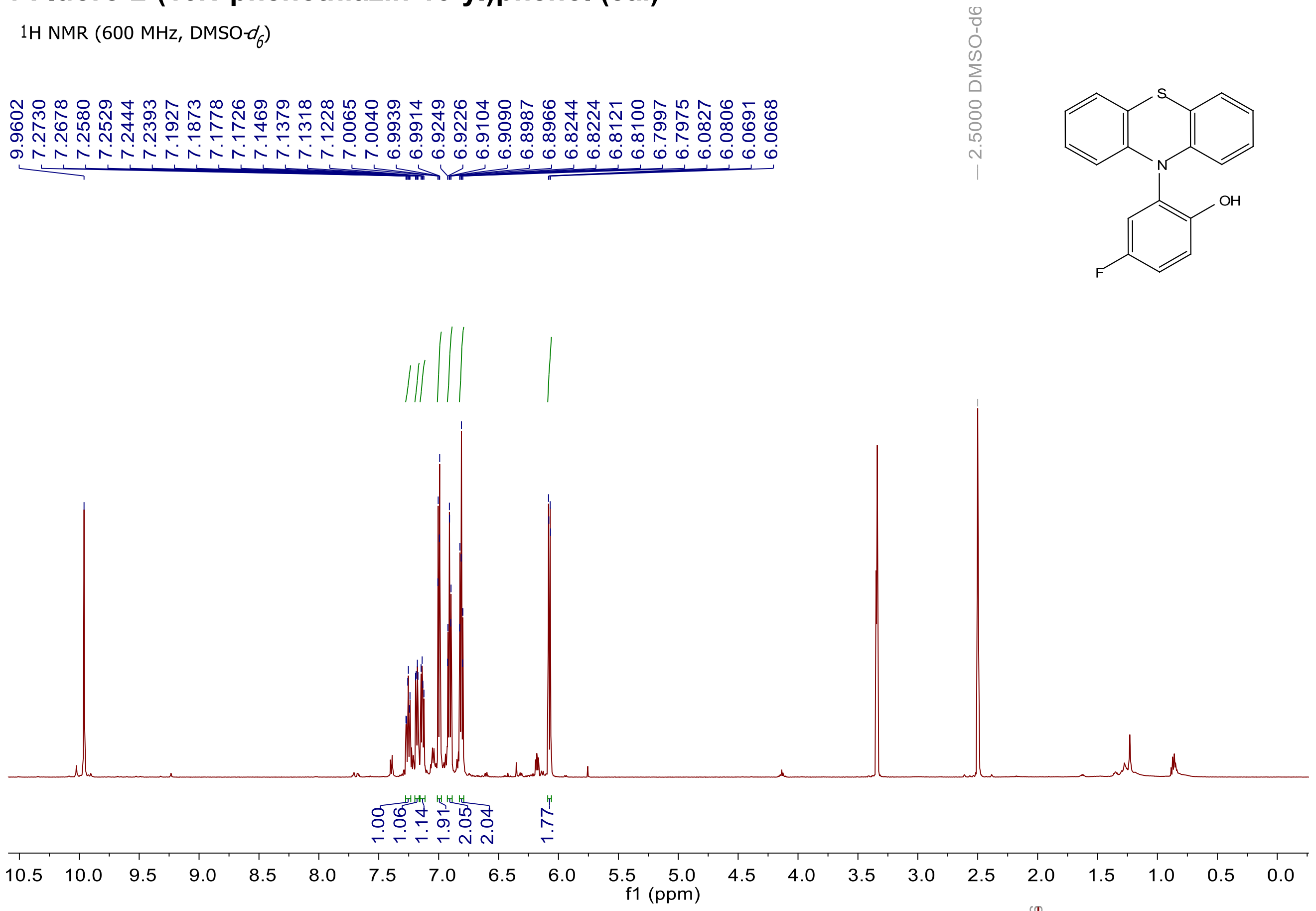

^{13}C{^{1}H} NMR (151 MHz, DMSO-d_6)

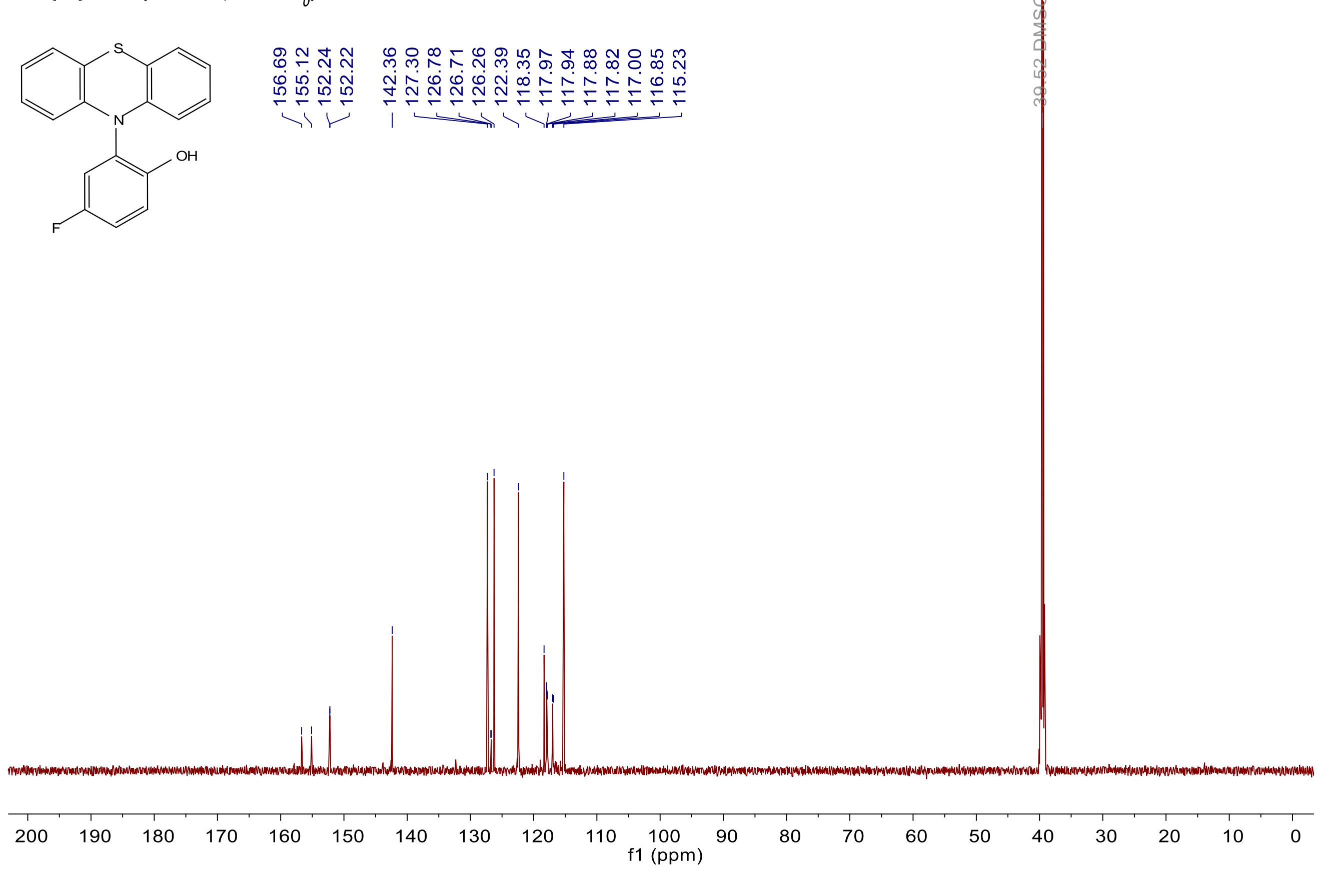

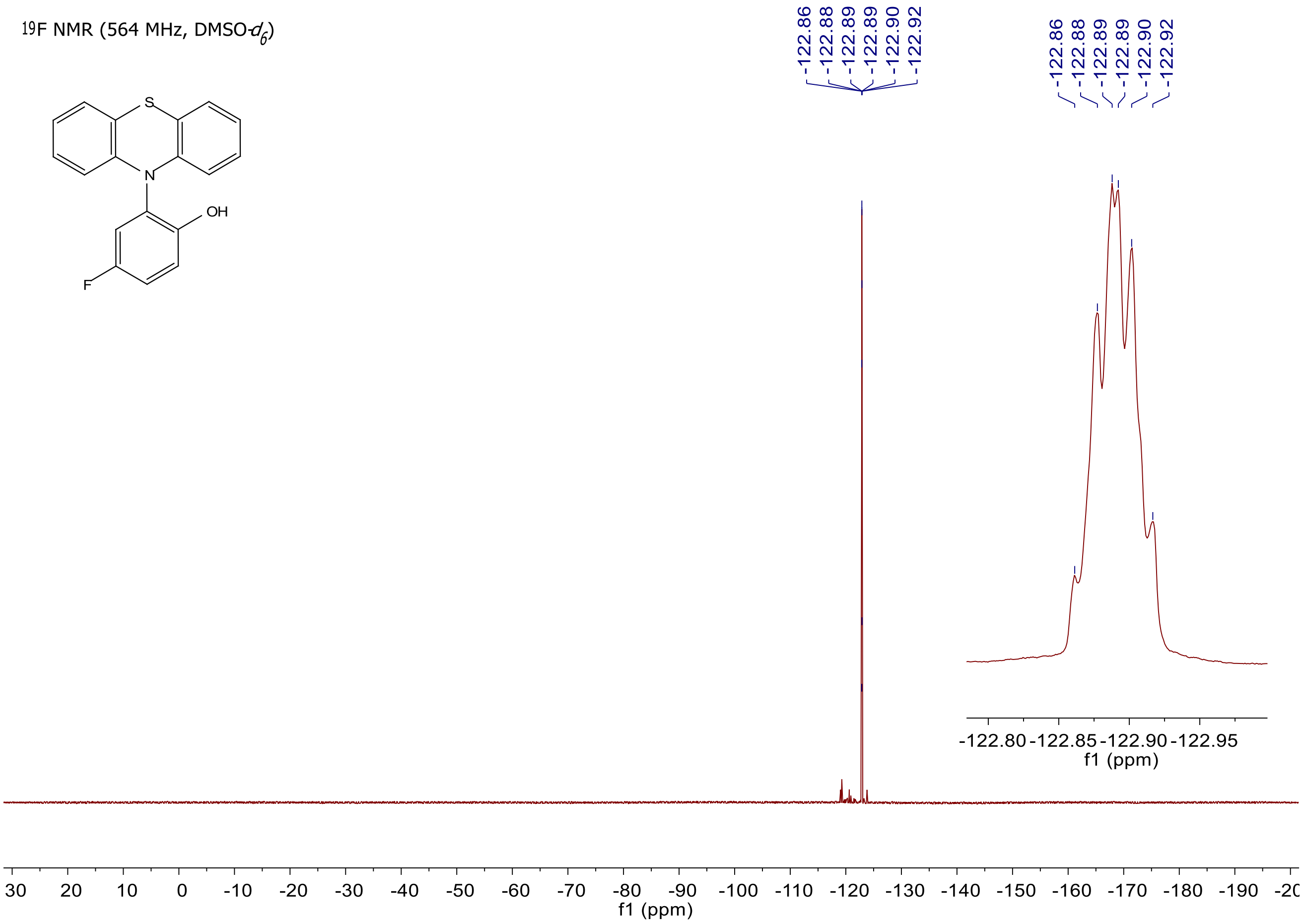

***N*-(4-Hydroxy-3-(10*H*-phenothiazin-10-yl)phenyl)acetamide (3al)**

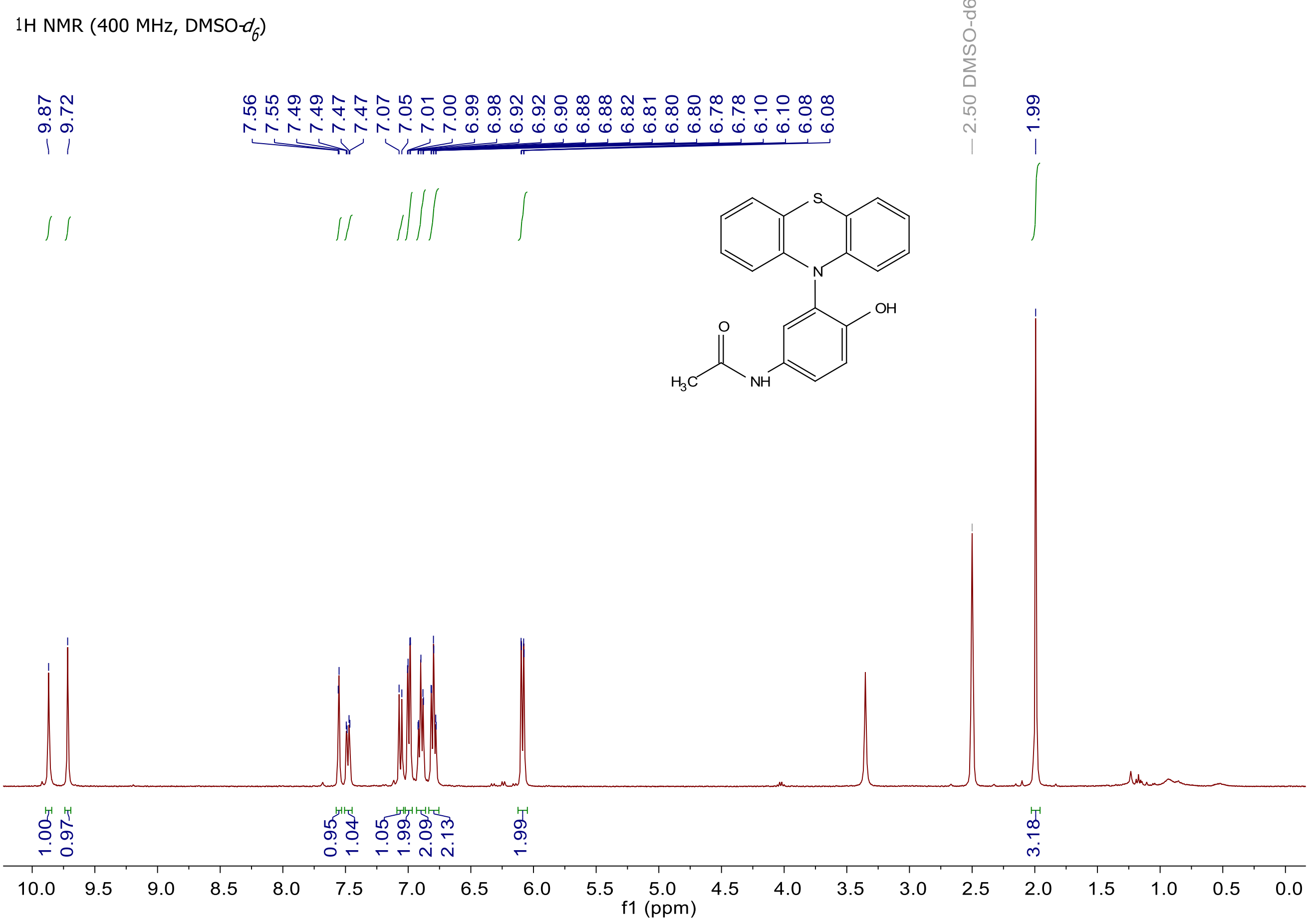

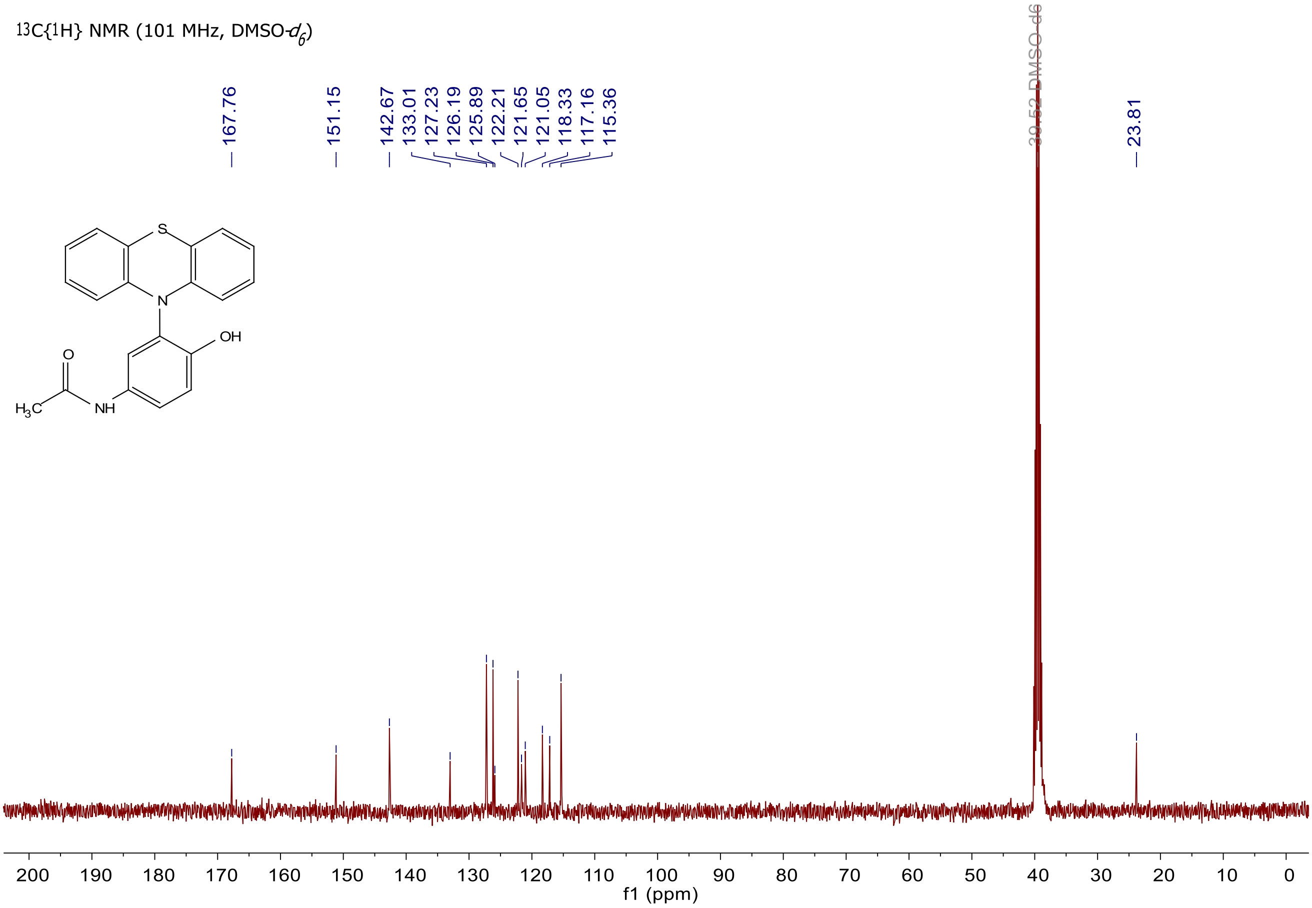

(2*S*,3*R*,4*S*,5*S*,6*R*)-2-[4-Hydroxy-3-(10*H*-phenothiazin-10-yl)phenoxy]-6-(hydroxymethyl)-tetrahydro-2*H*-pyran-3,4,5-triol (3am)

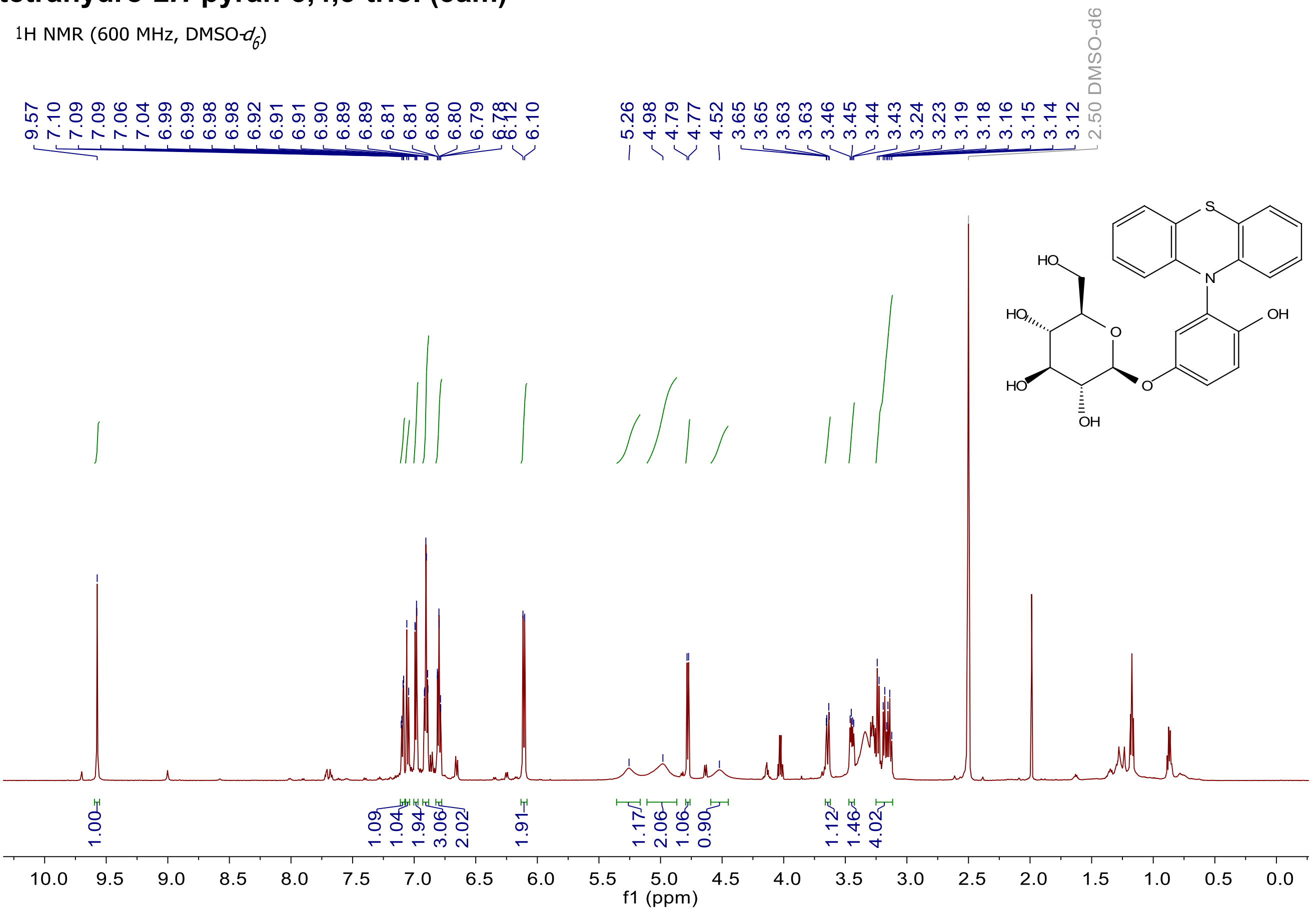

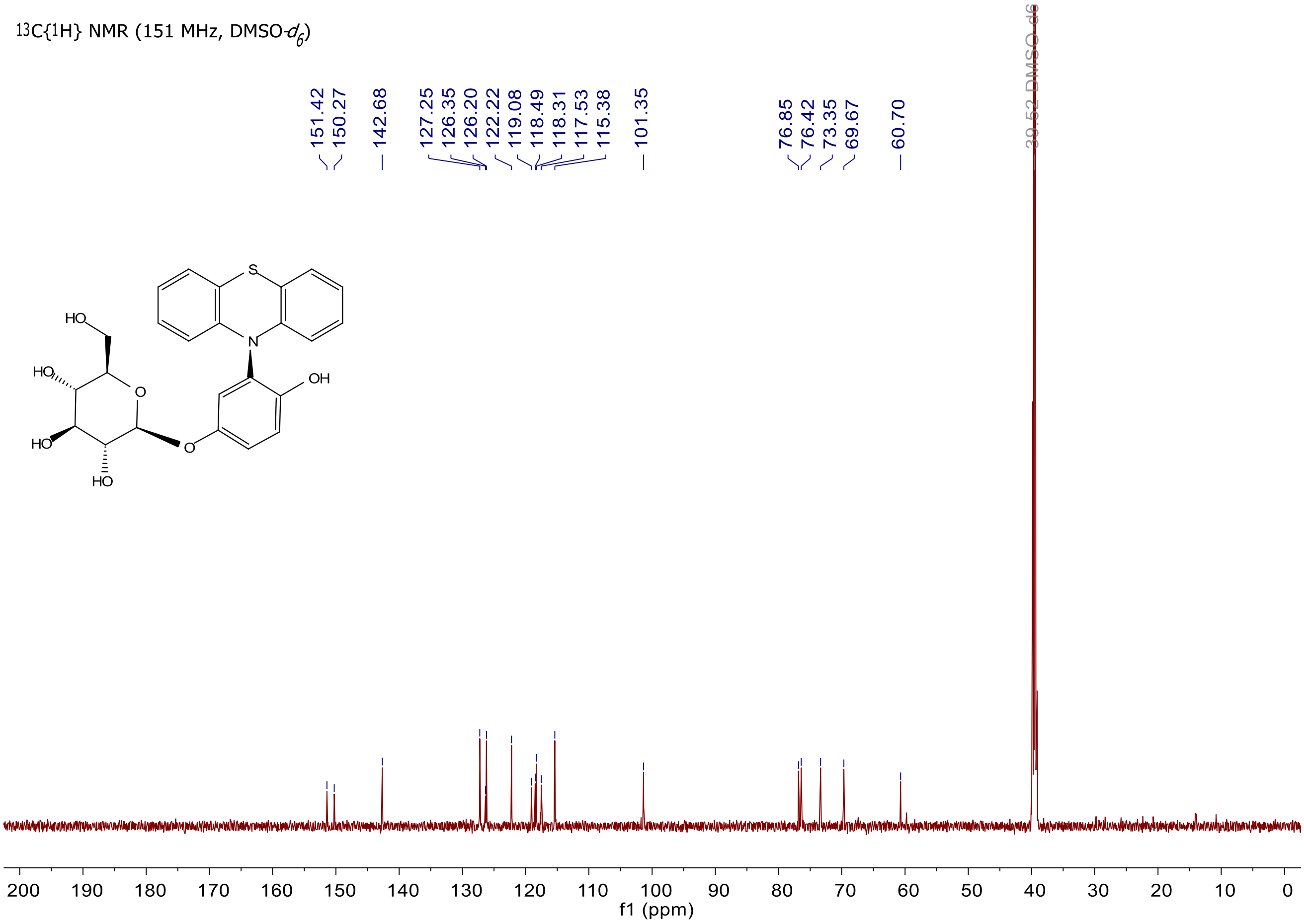

4-(*tert*-Butyl)-2-[2-(trifluoromethyl)-10*H*-phenothiazin-10-yl]phenol (3ba)

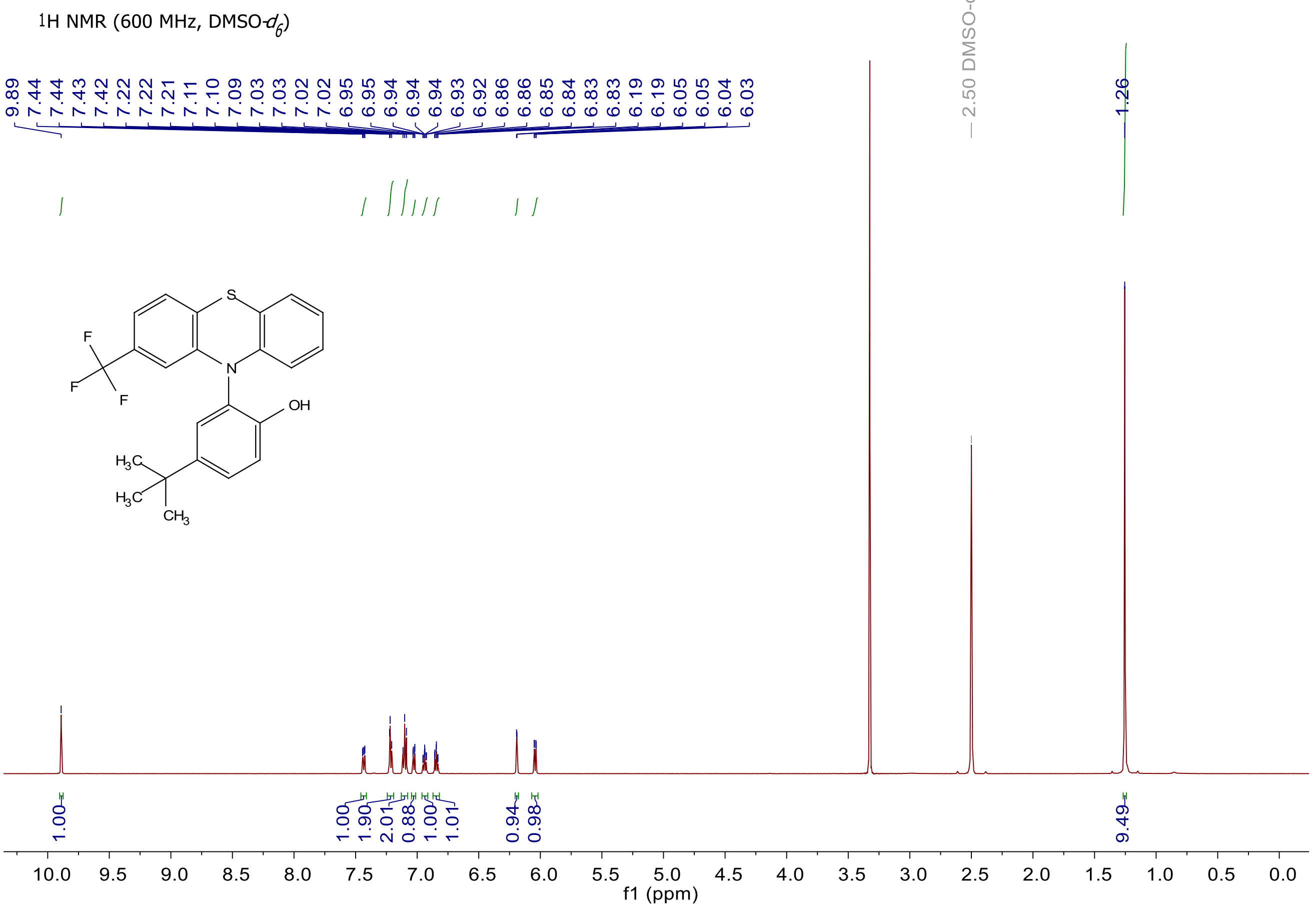

$^{13}C\{^1H\}$ NMR (151 MHz, DMSO-d_6)

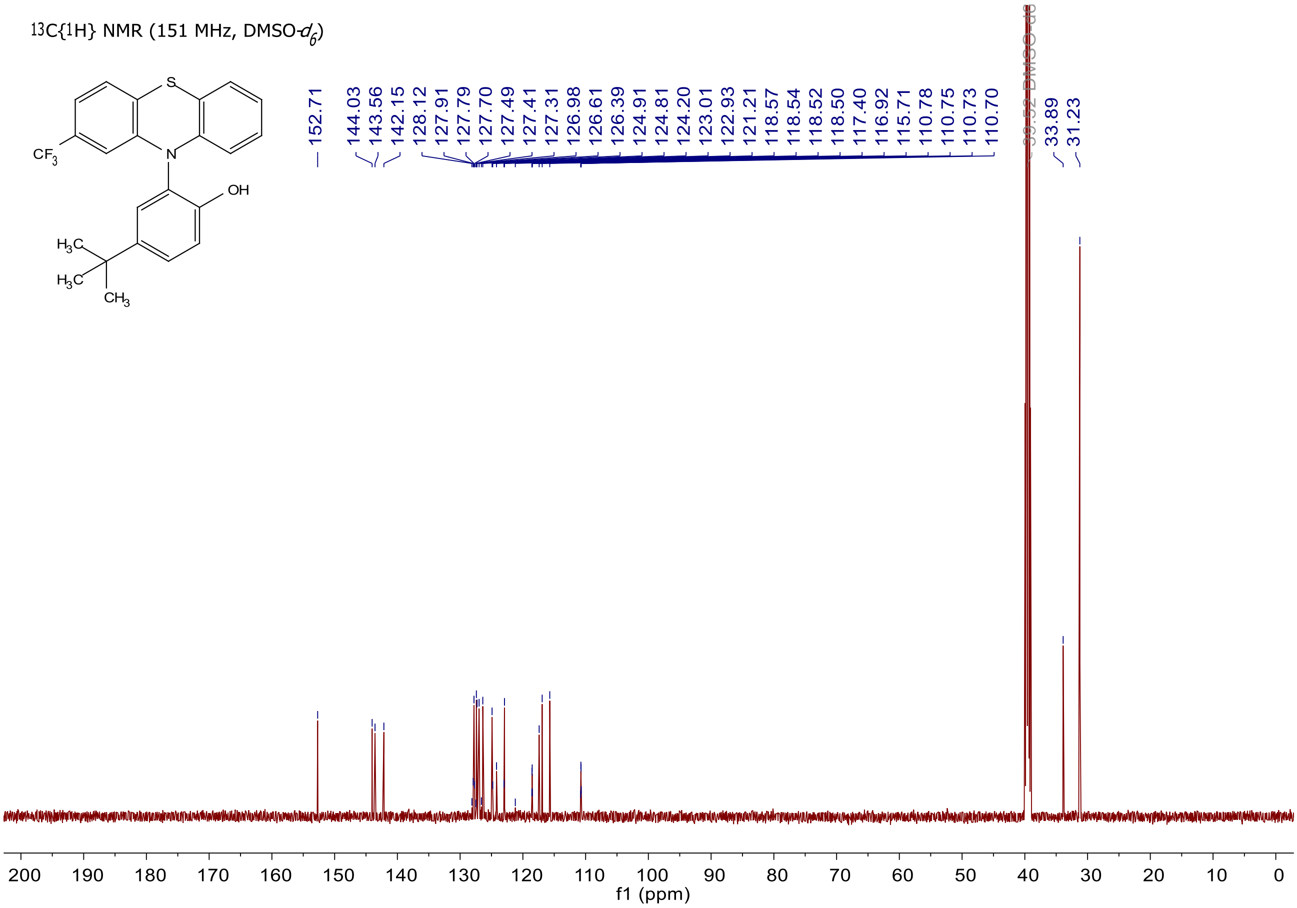

^{19}F NMR (564 MHz, DMSO-d_6)

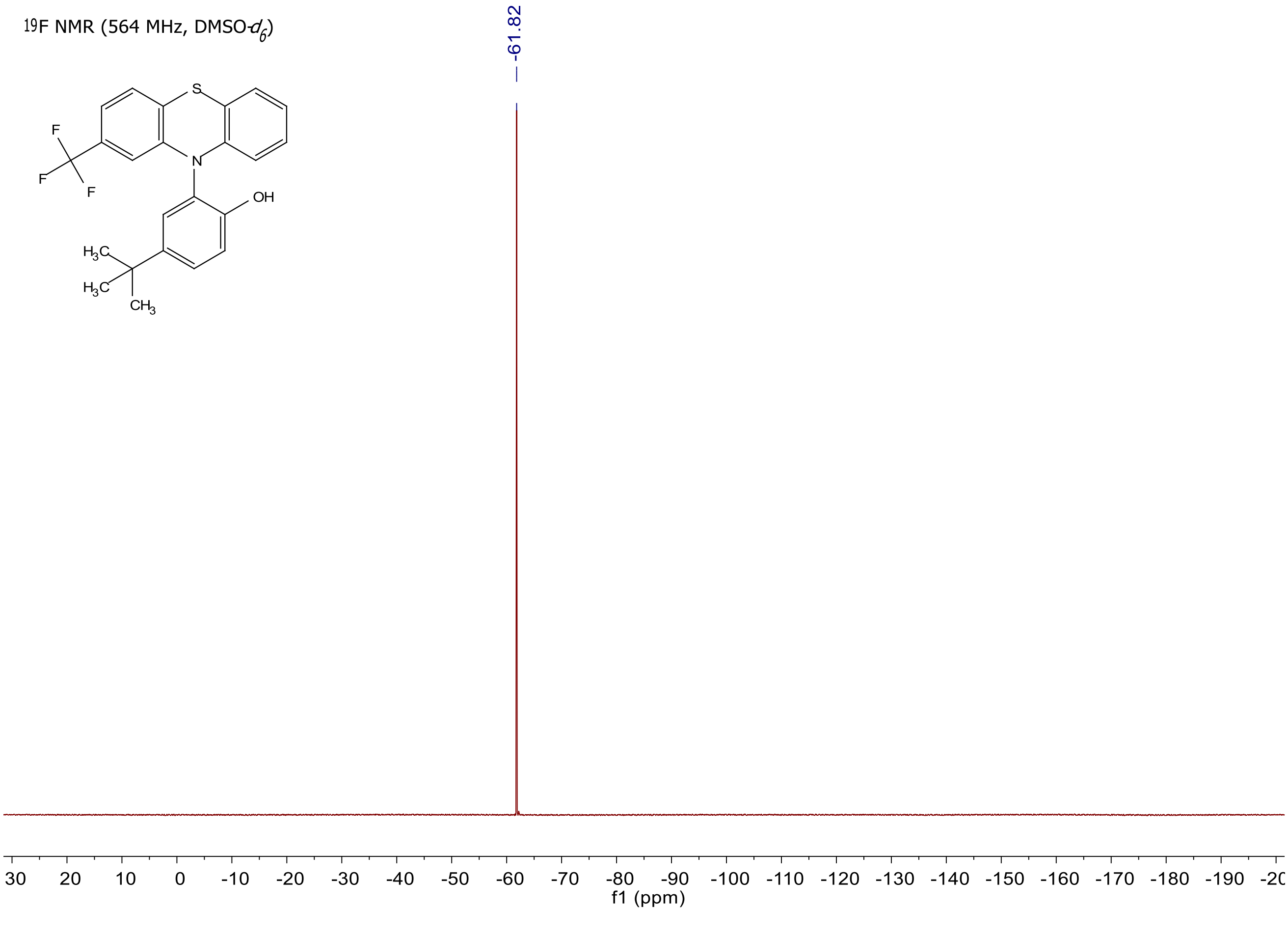

1-{10-[5-(*tert*-butyl)-2-hydroxyphenyl]-10*H*-phenothiazin-2-yl}ethan-1-one (3ca)

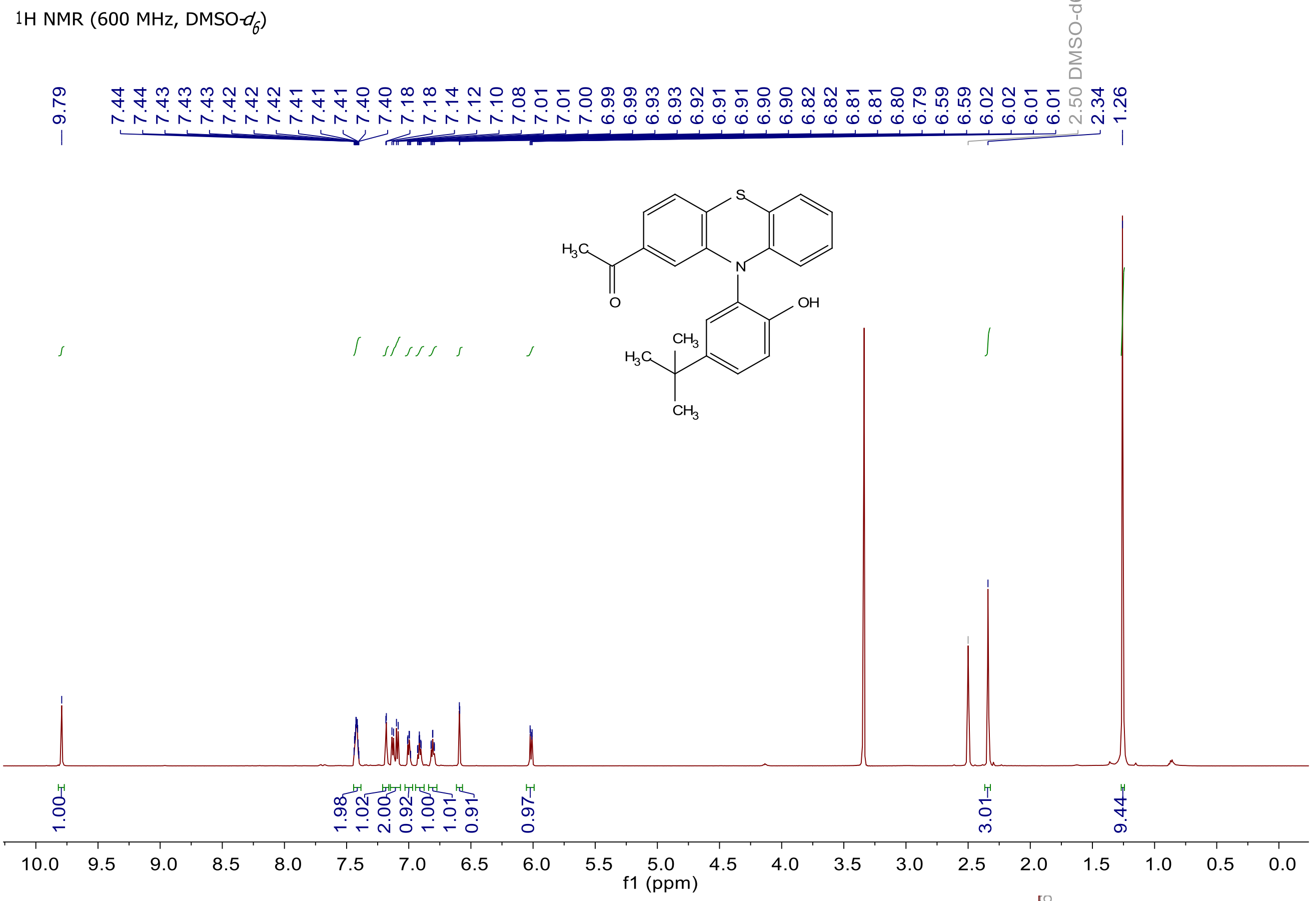

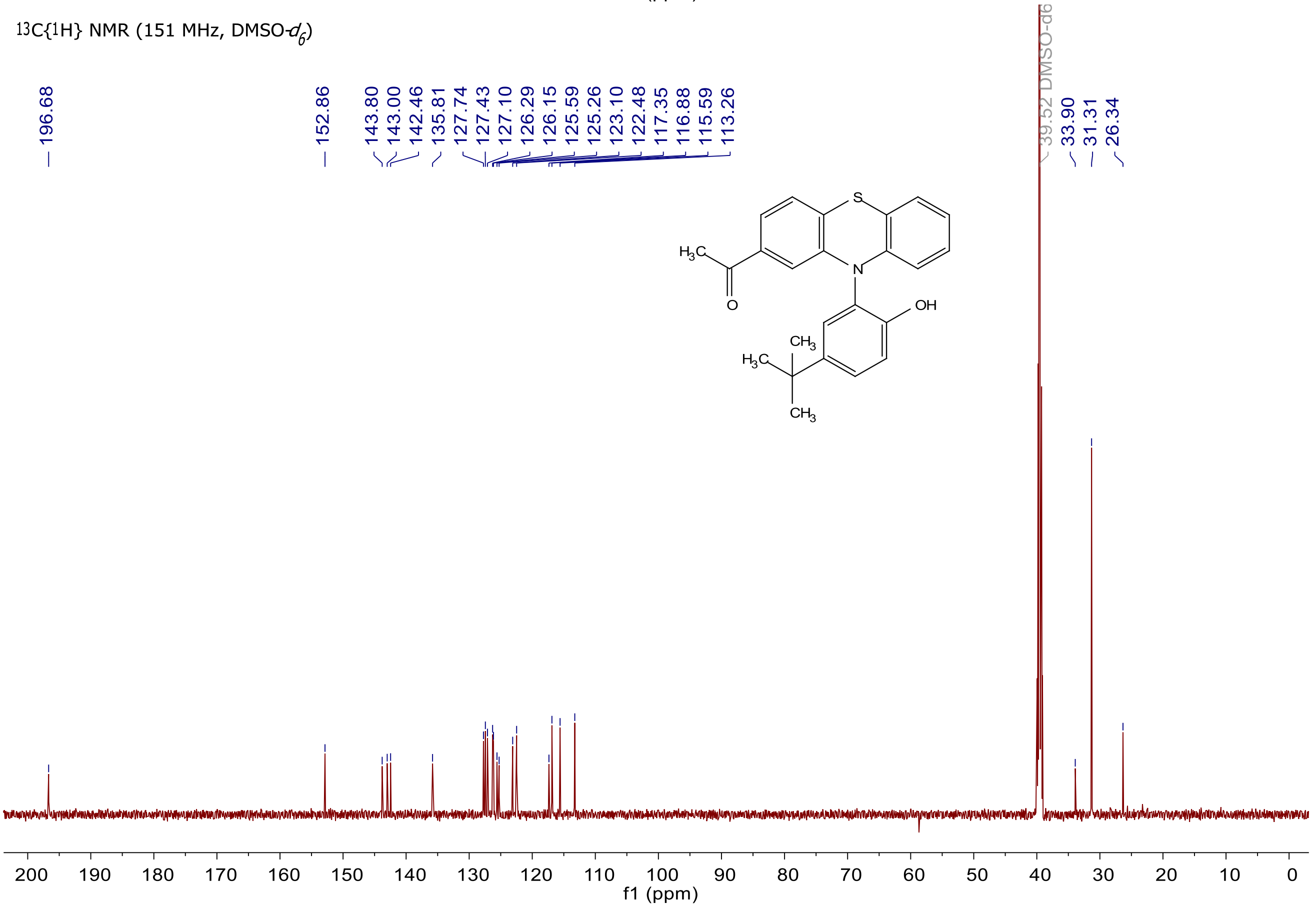

4-(*tert*-Butyl)-2-(2-chloro-10*H*-phenothiazin-10-yl)phenol (3da)

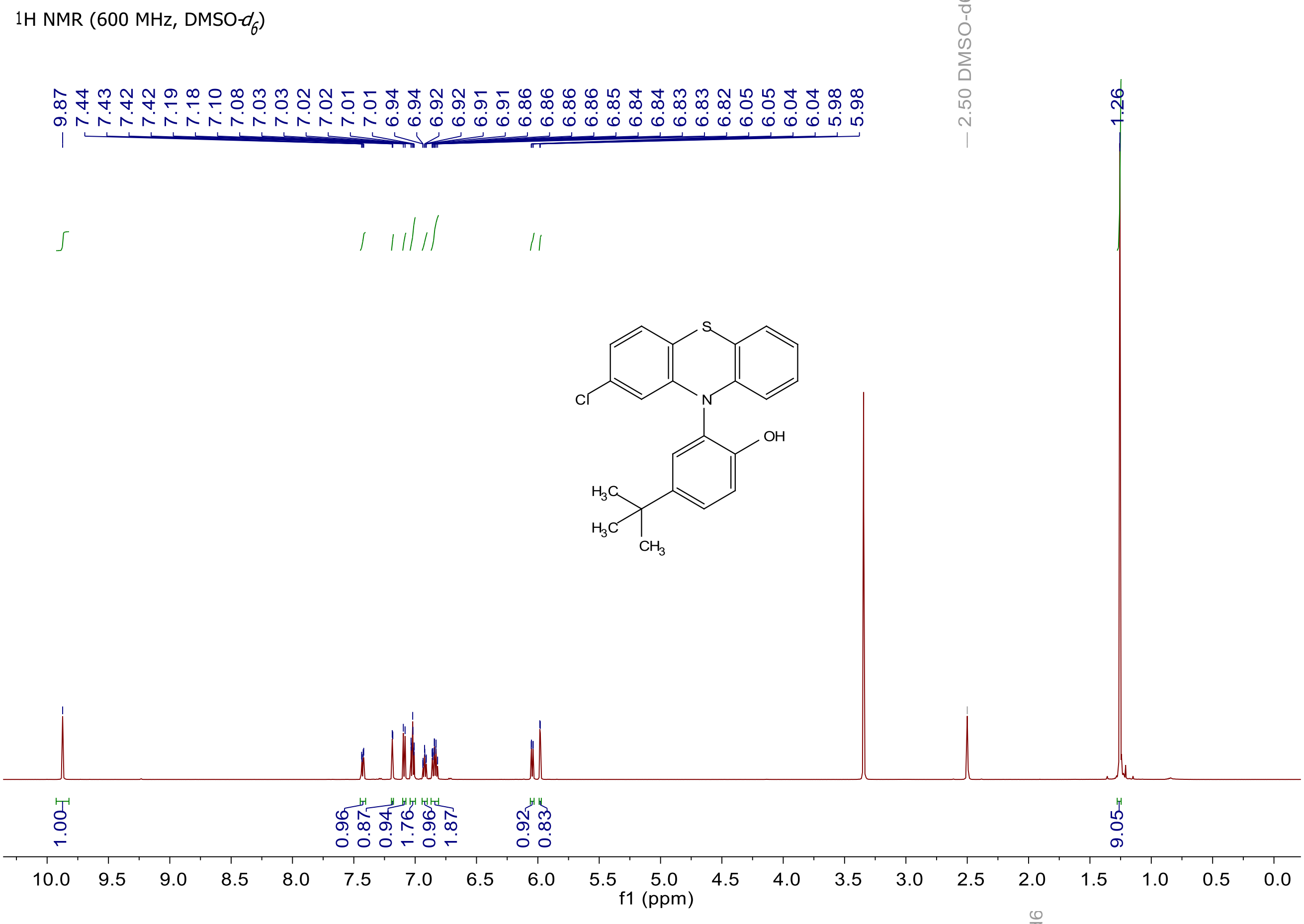

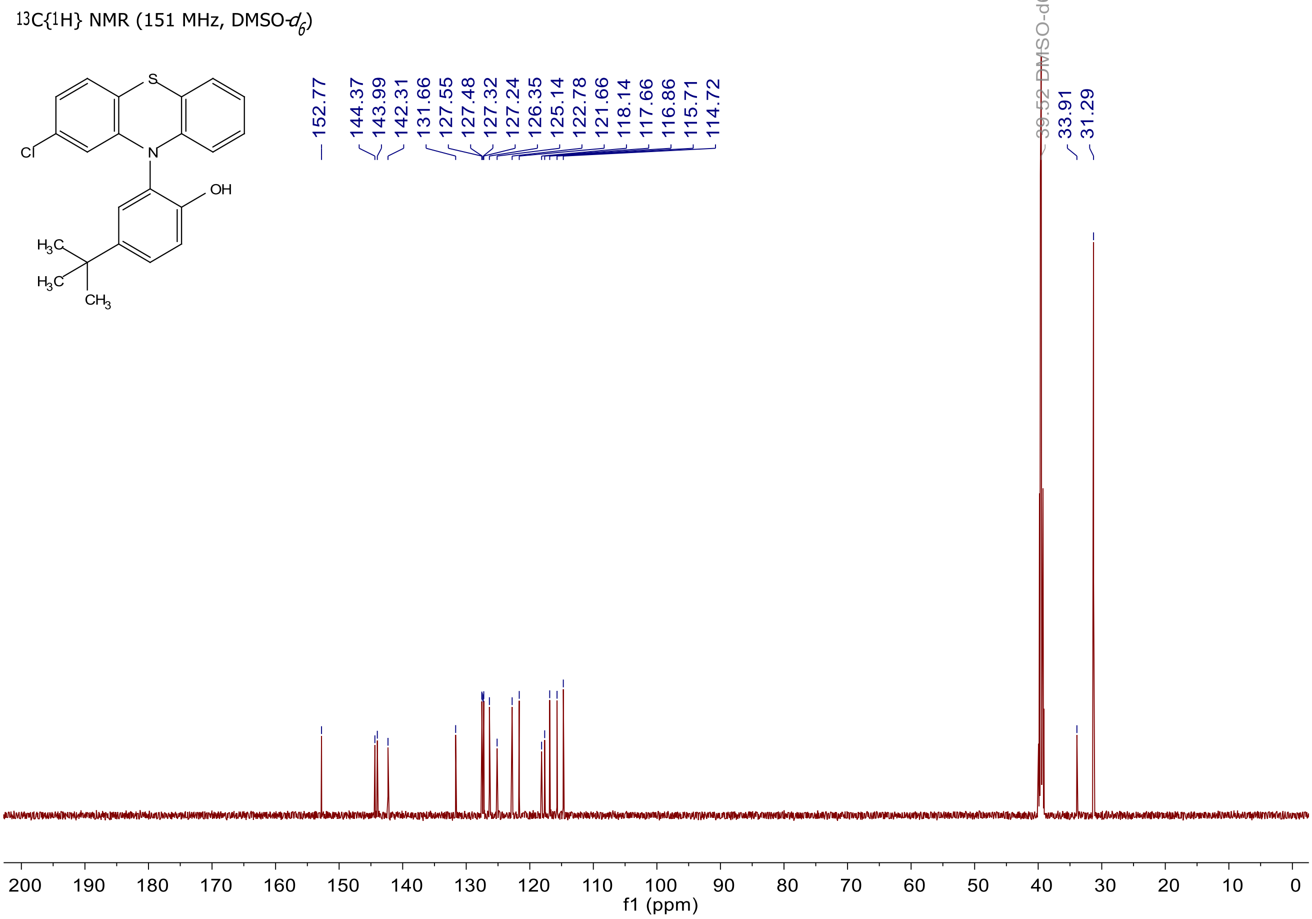

10-(6-Hydroxy-2,3,4-trimethylphenyl)-10*H*-phenothiazine-2-carbonitrile (3en)

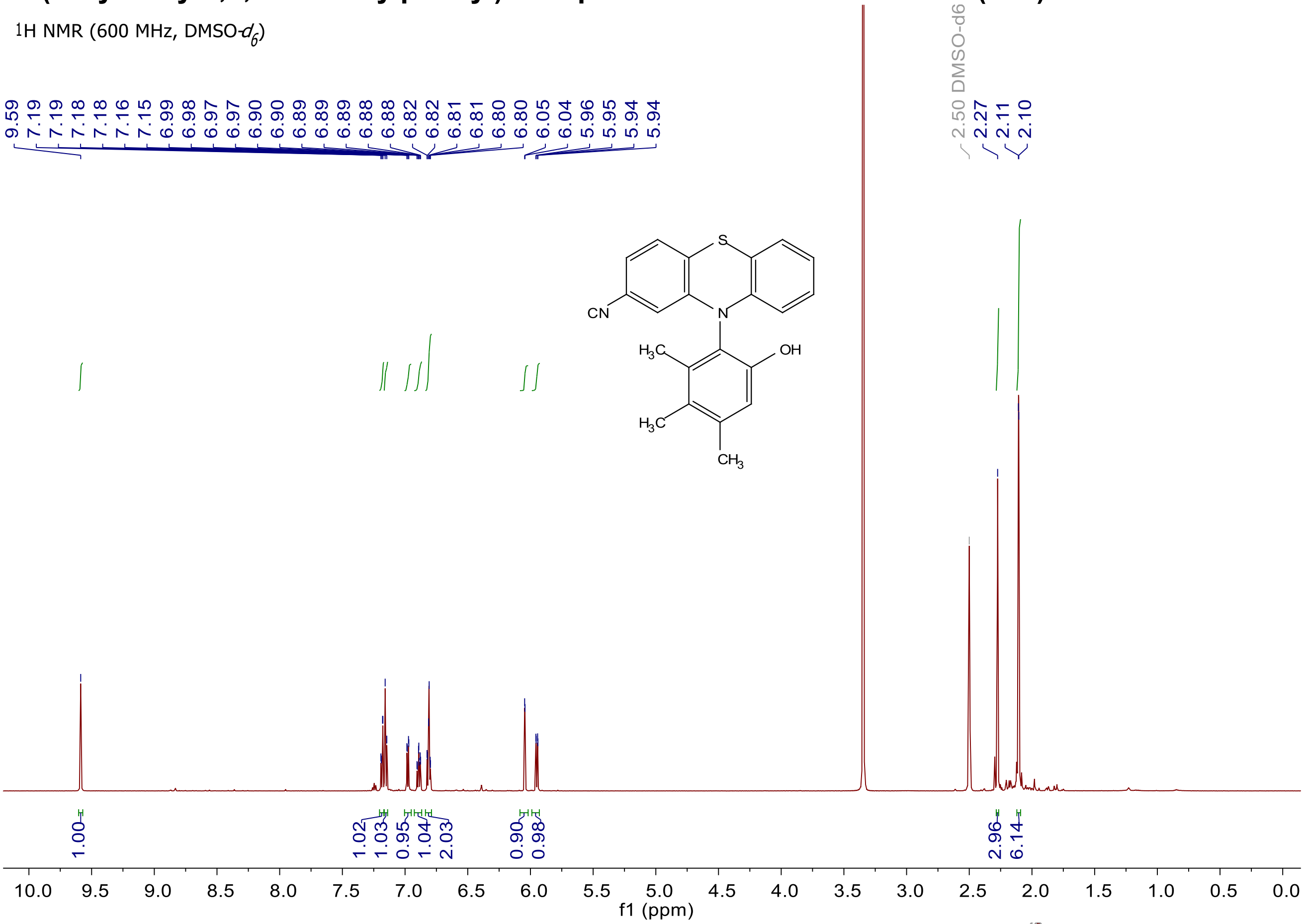

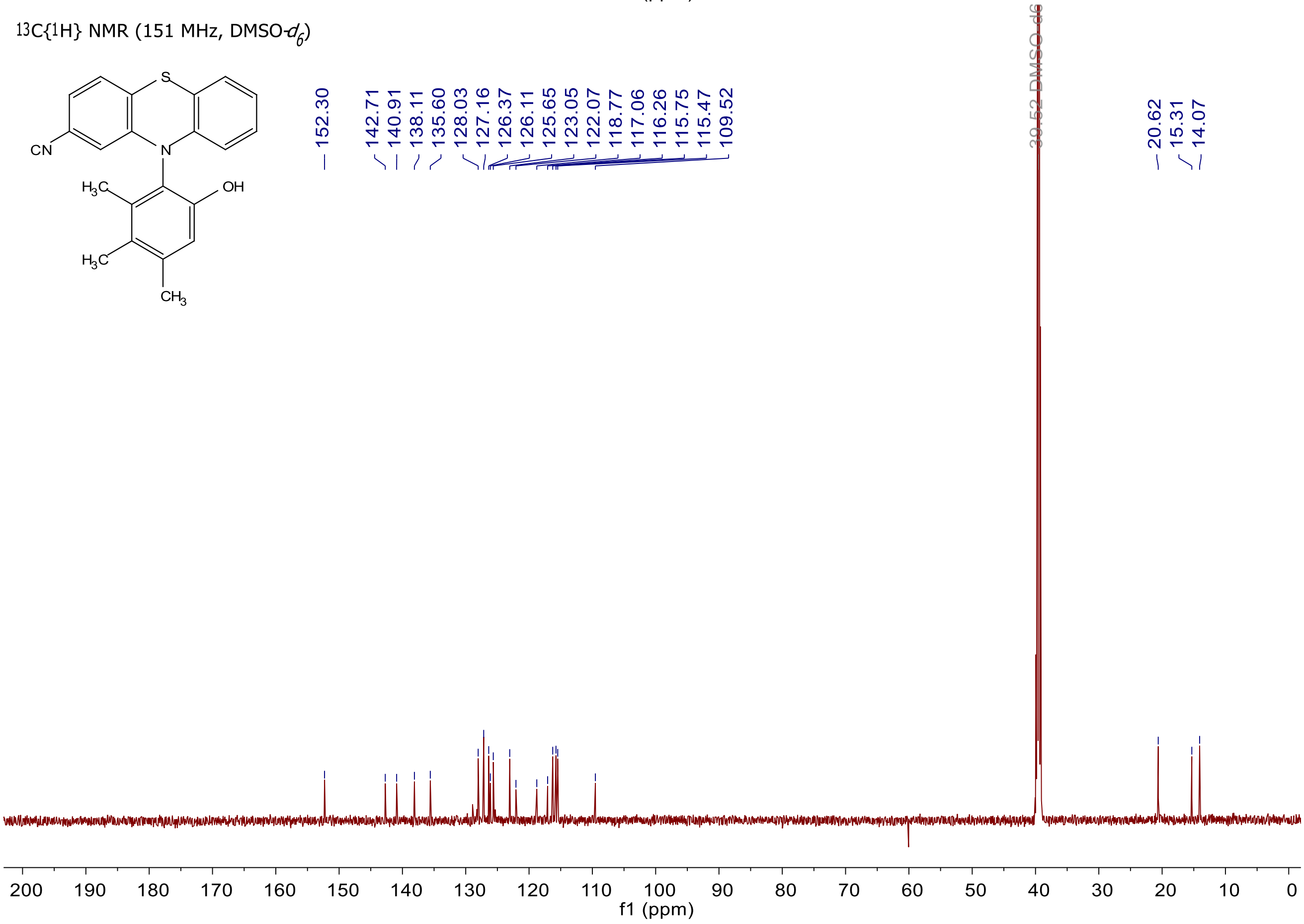

2-(2-Methoxy-10*H*-phenothiazin-10-yl)-3,4,5-trimethylphenol (3fn)

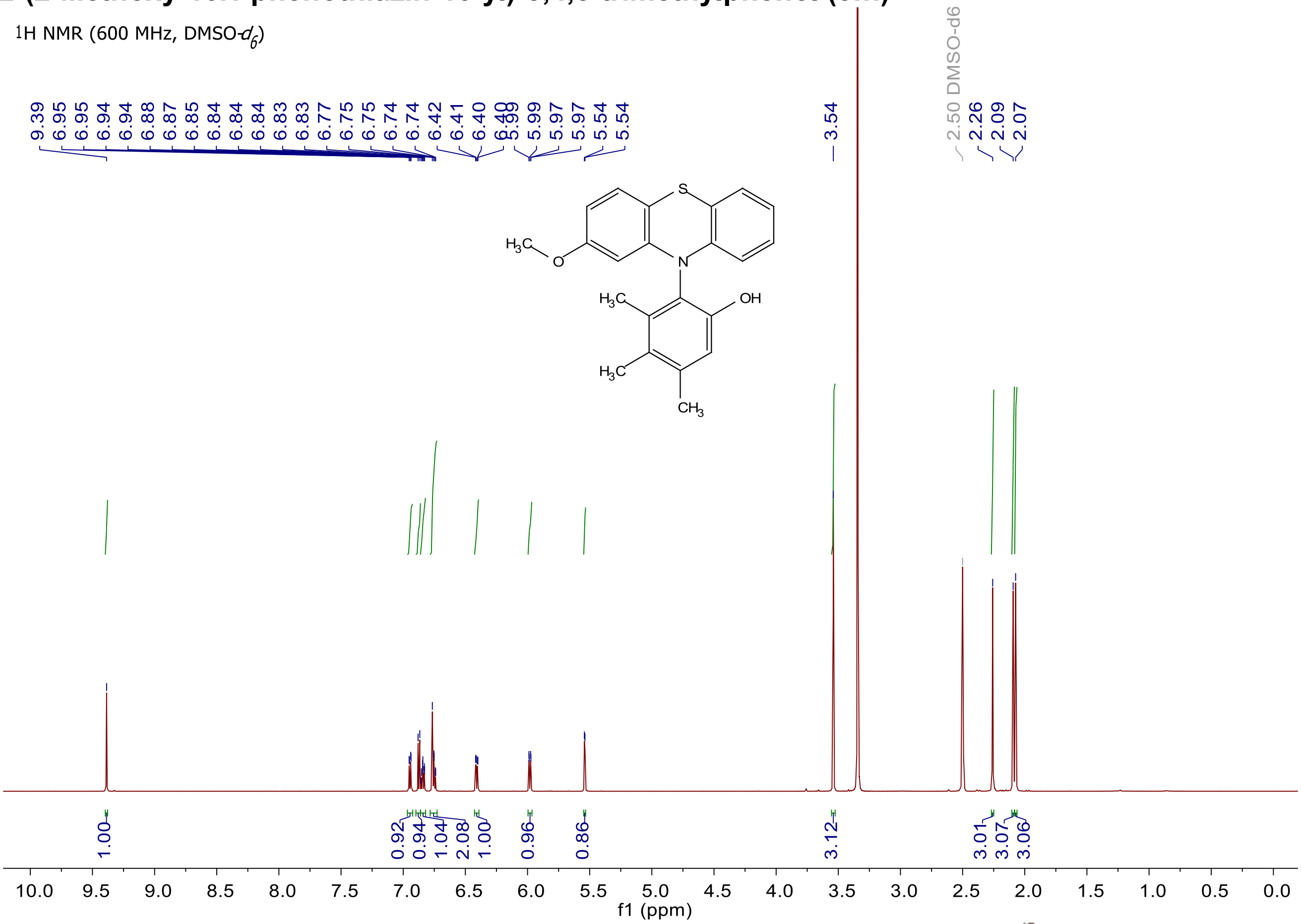

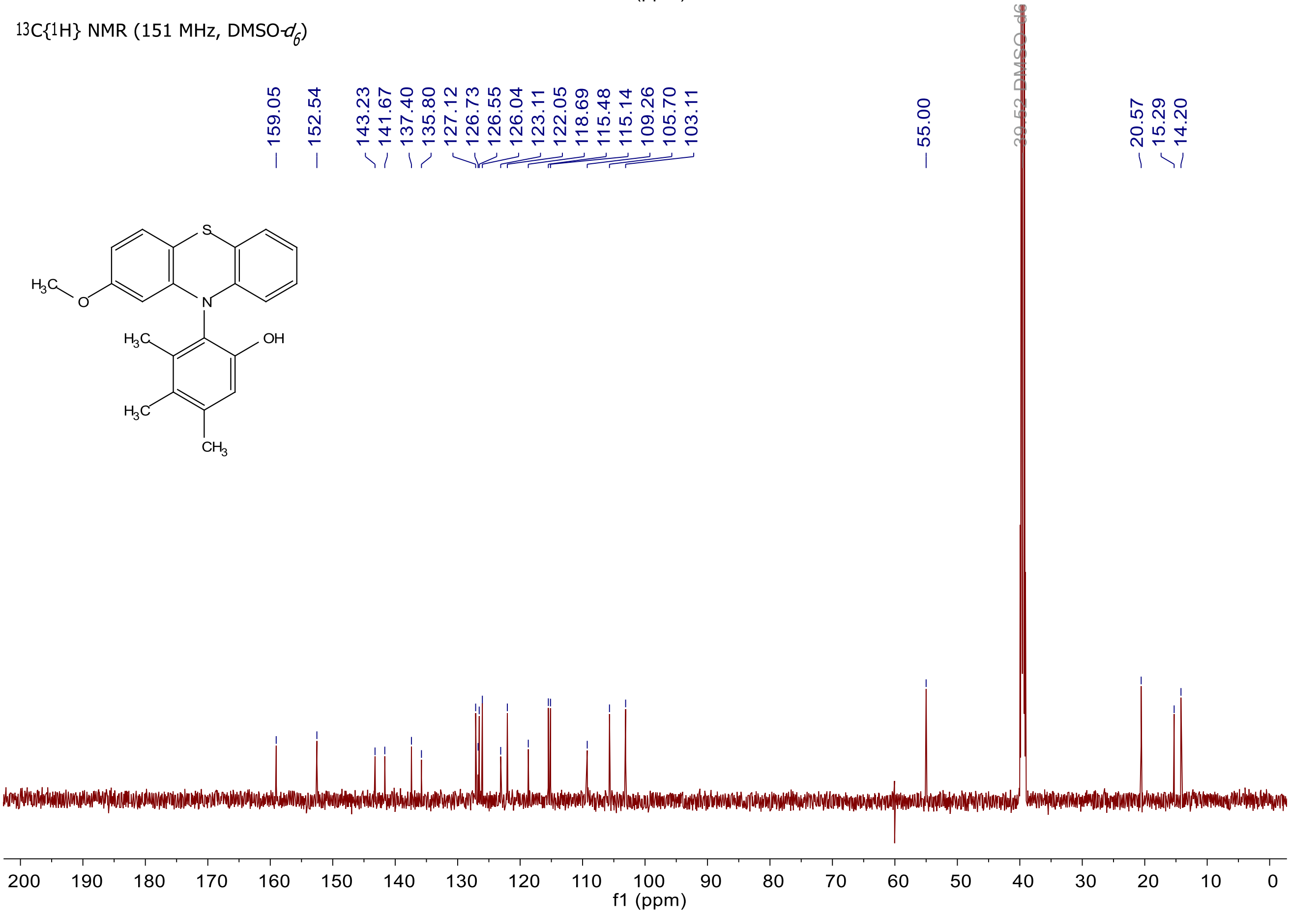

3,4,5-Trimethyl-2-[2-(methylthio)-10*H*-phenothiazin-10-yl]phenol (3gn)

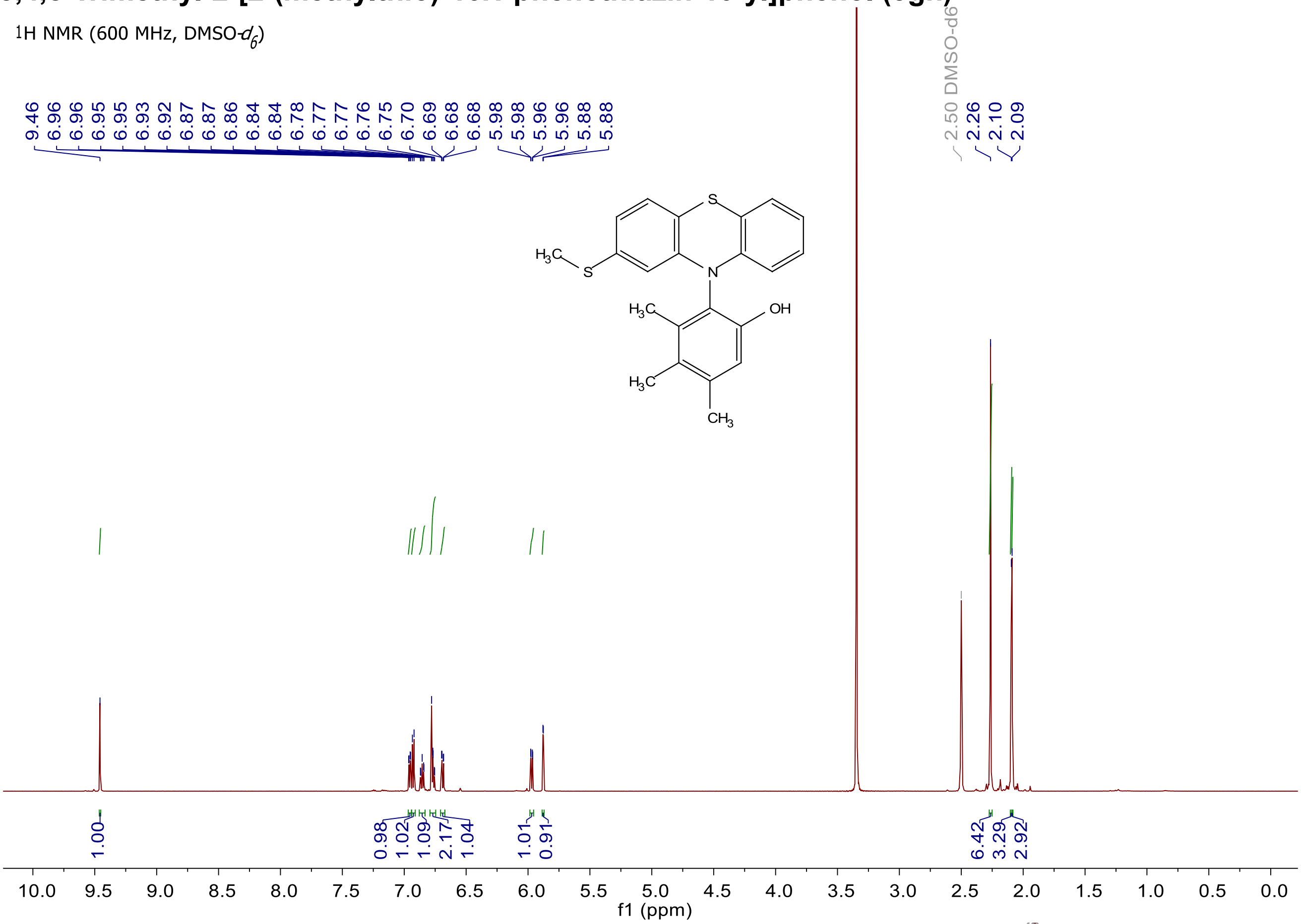

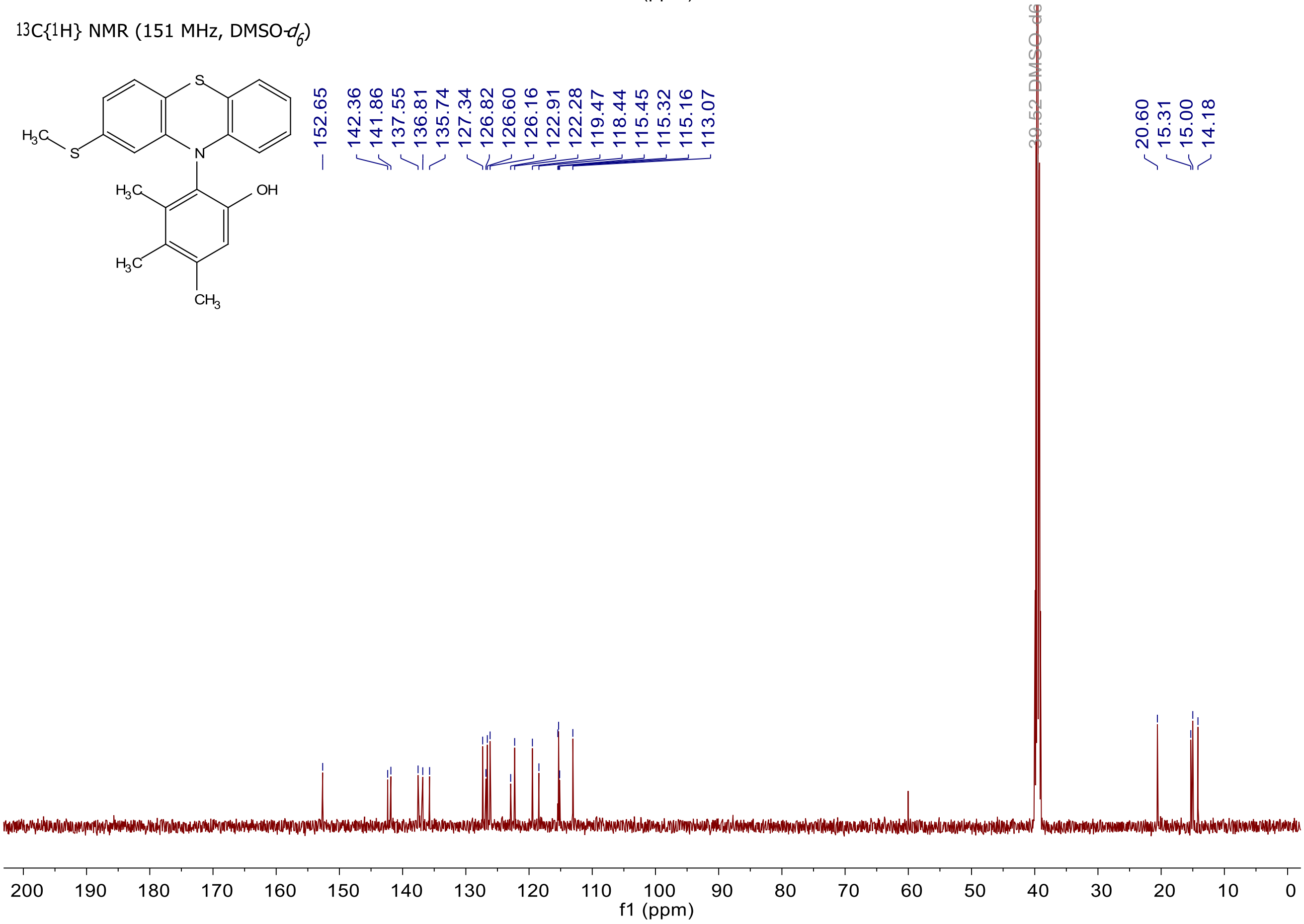

3,4,5-Trimethyl-2-(10*H*-phenoxazin-10-yl)phenol (3hn)

^{1}H NMR (600 MHz, DMSO-d_6)

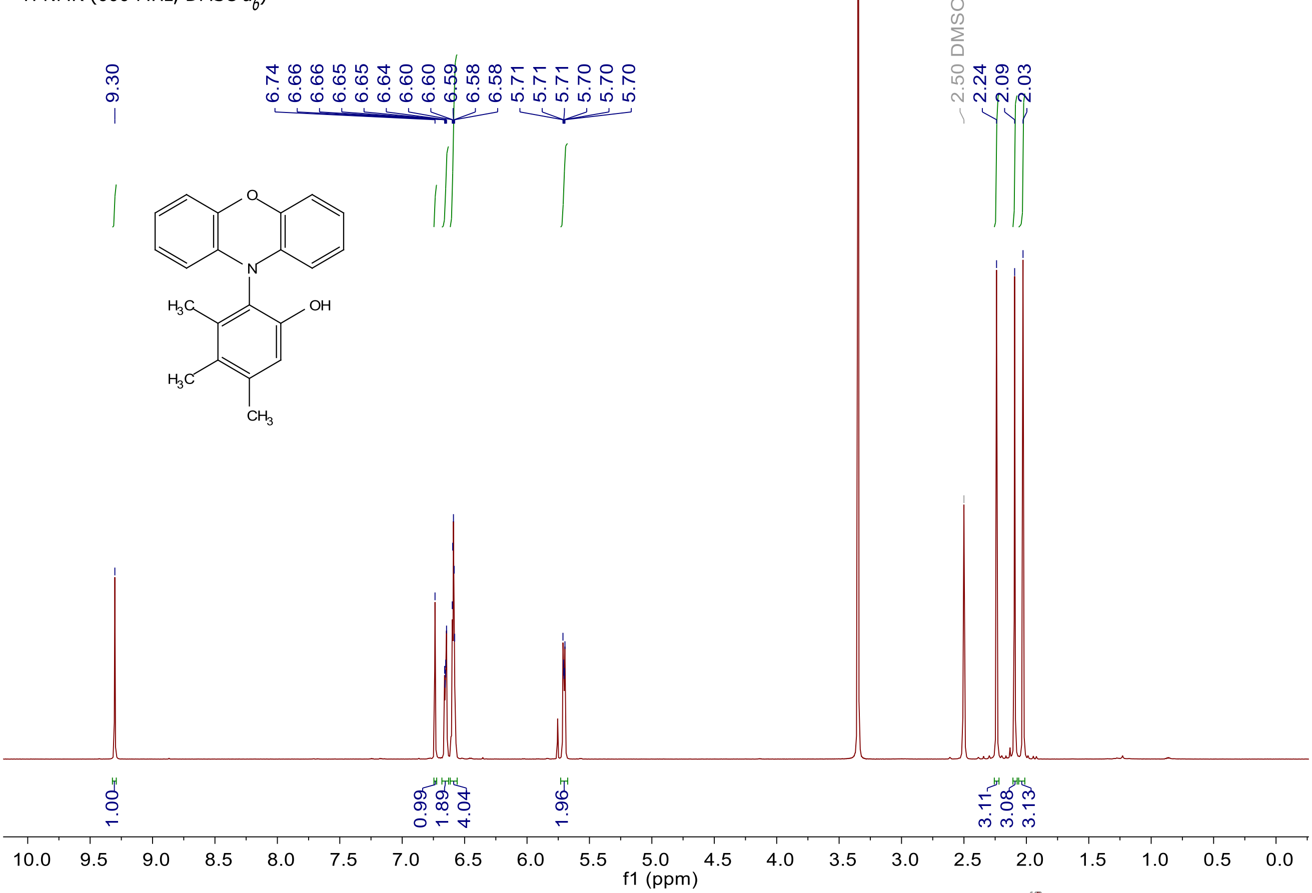

$^{13}C\{^{1}H\}$ NMR (151 MHz, DMSO-d_6)

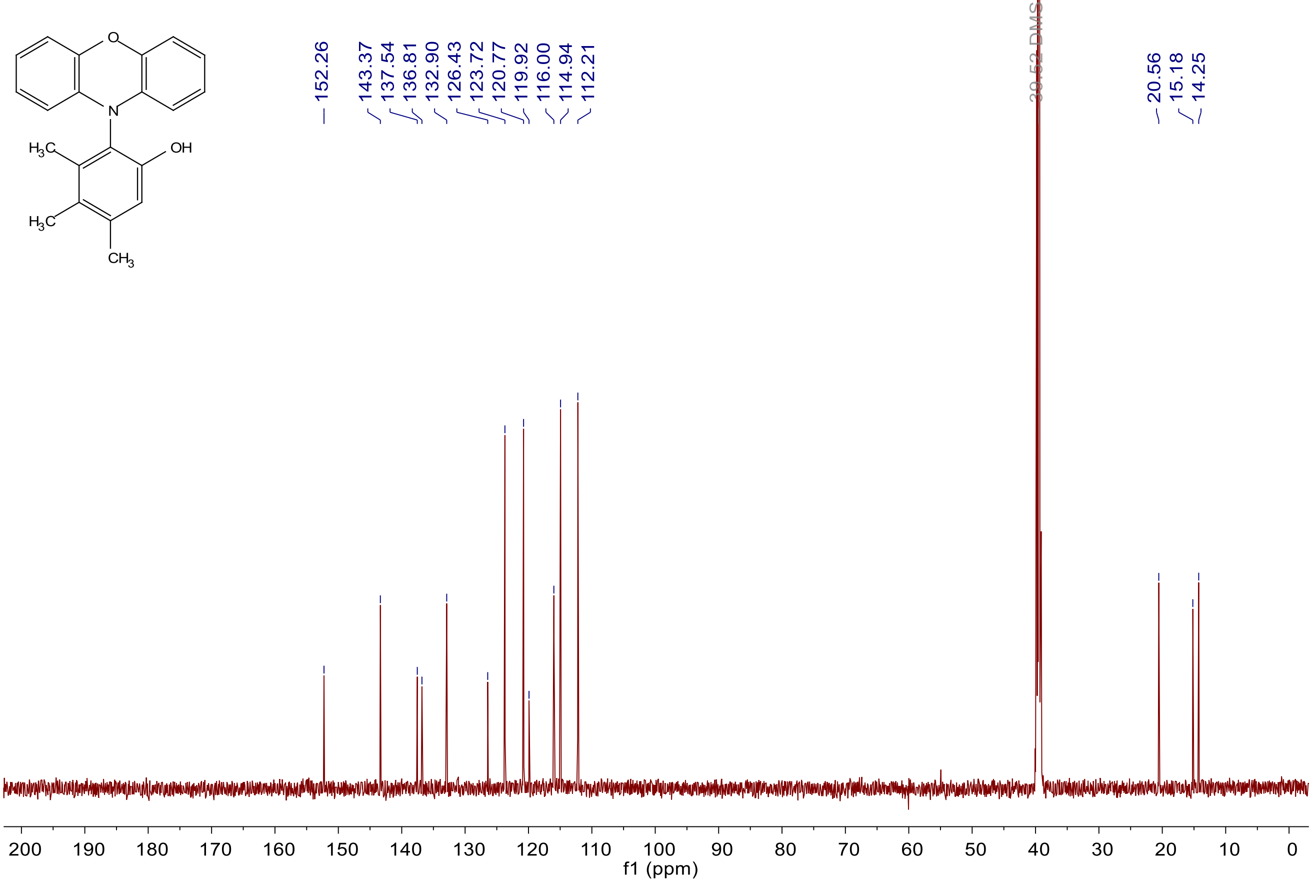

3,4,5-Trimethyl-2-(10*H*-phenothiazin-10-yl)phenol (3an)

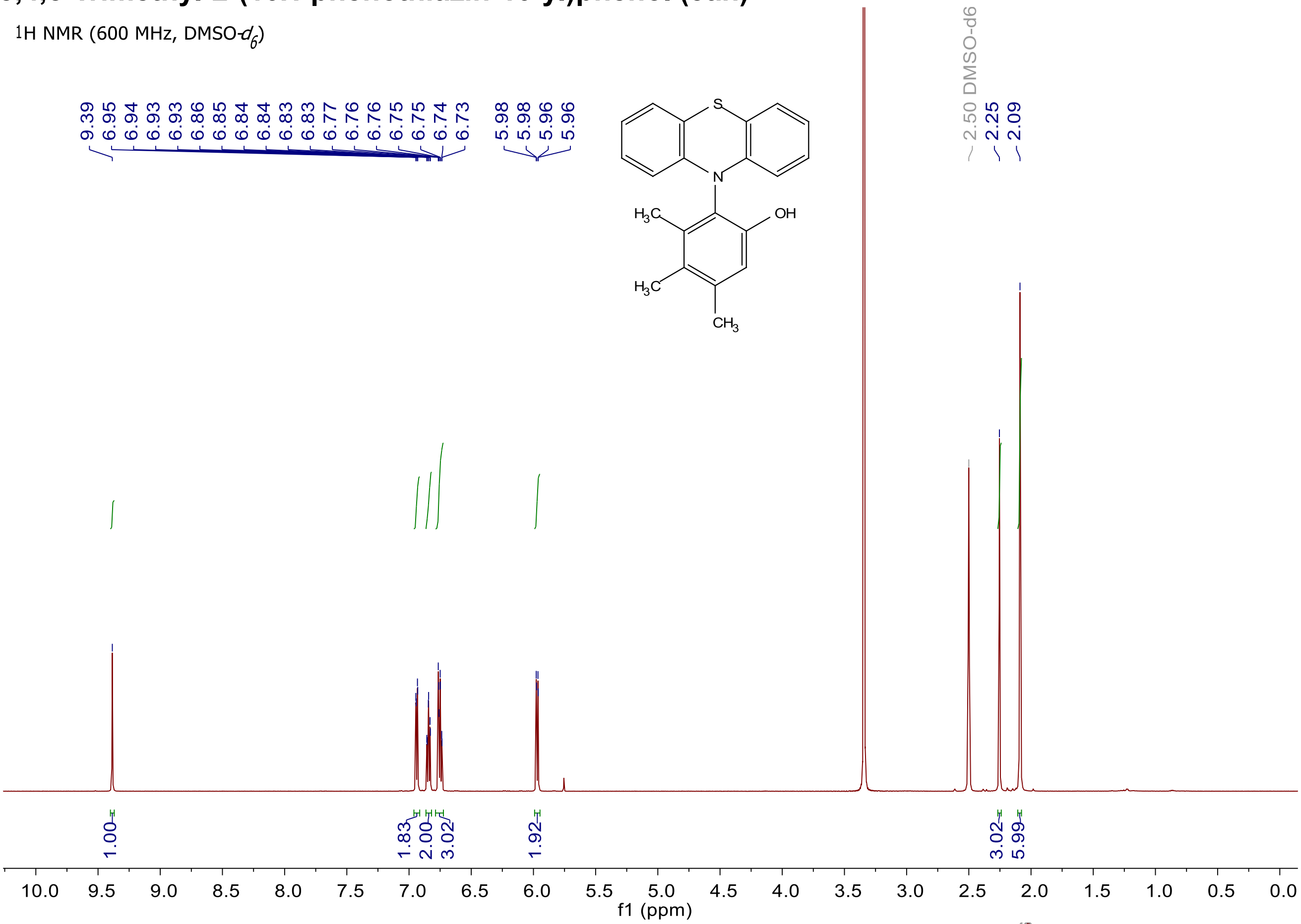

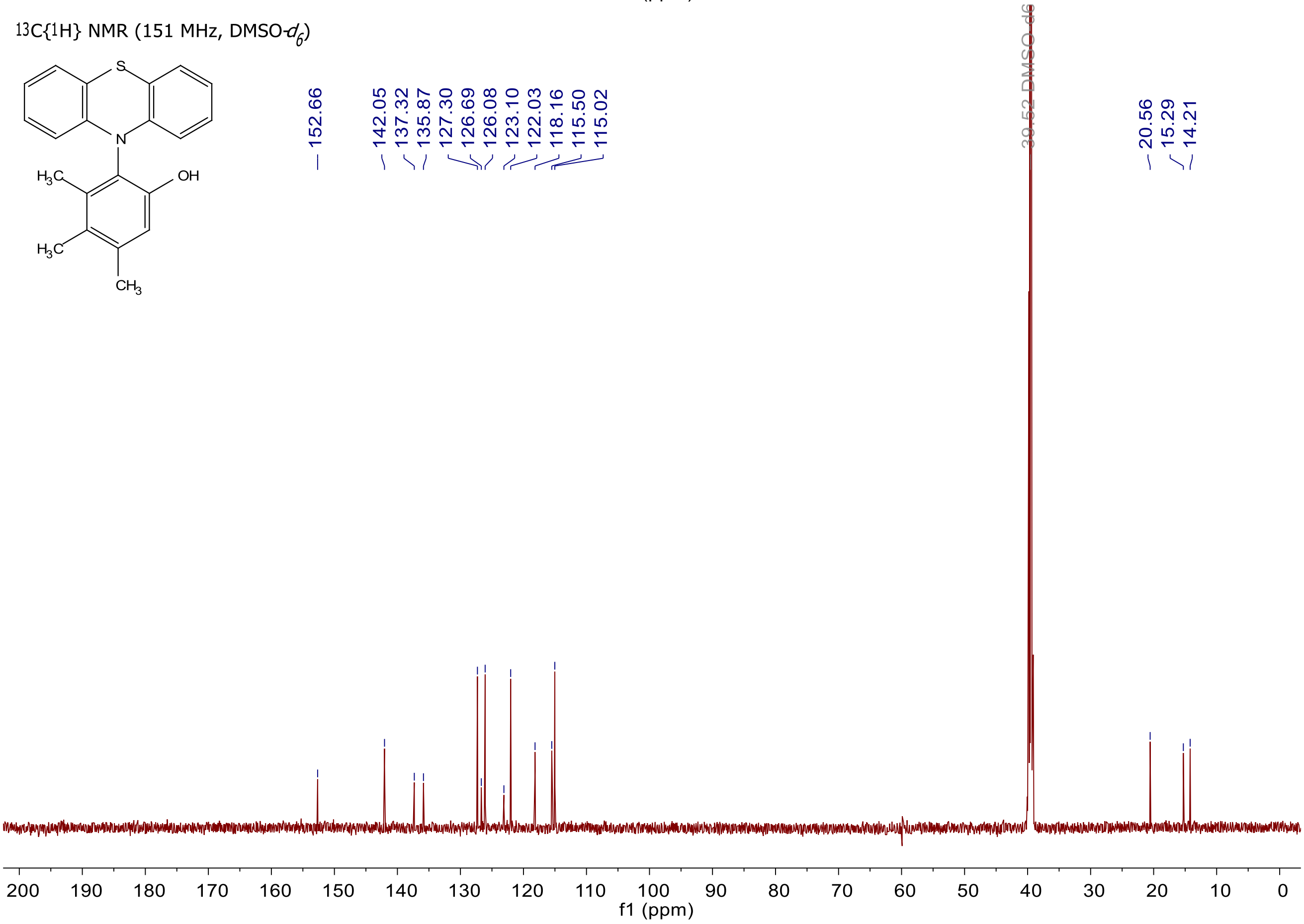

3,4,5-Trimethyl-2-(10*H*-phenoselenazin-10-yl)phenol (3in)

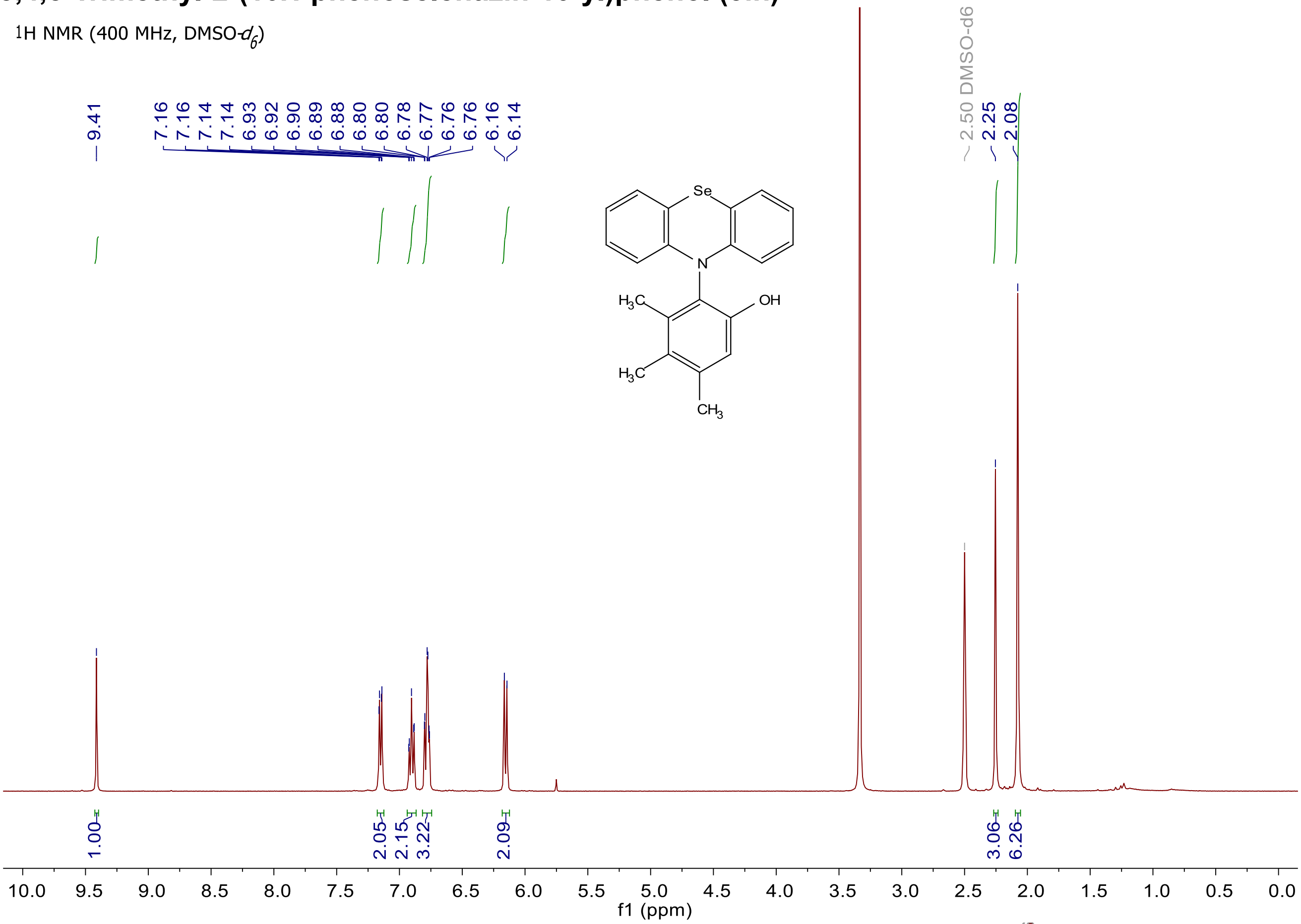

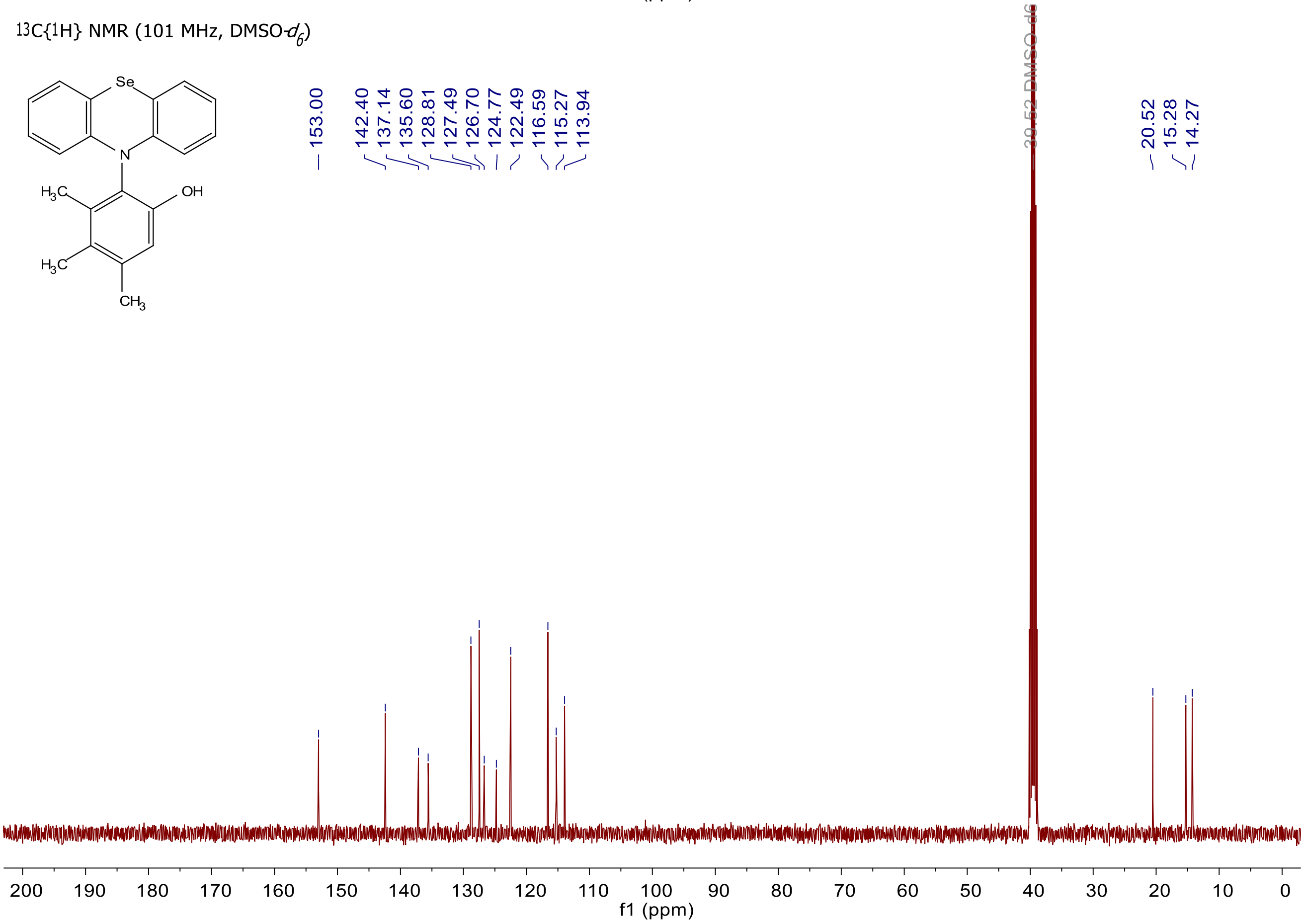

^{77}Se NMR (76 MHz, DMSO-d_6)

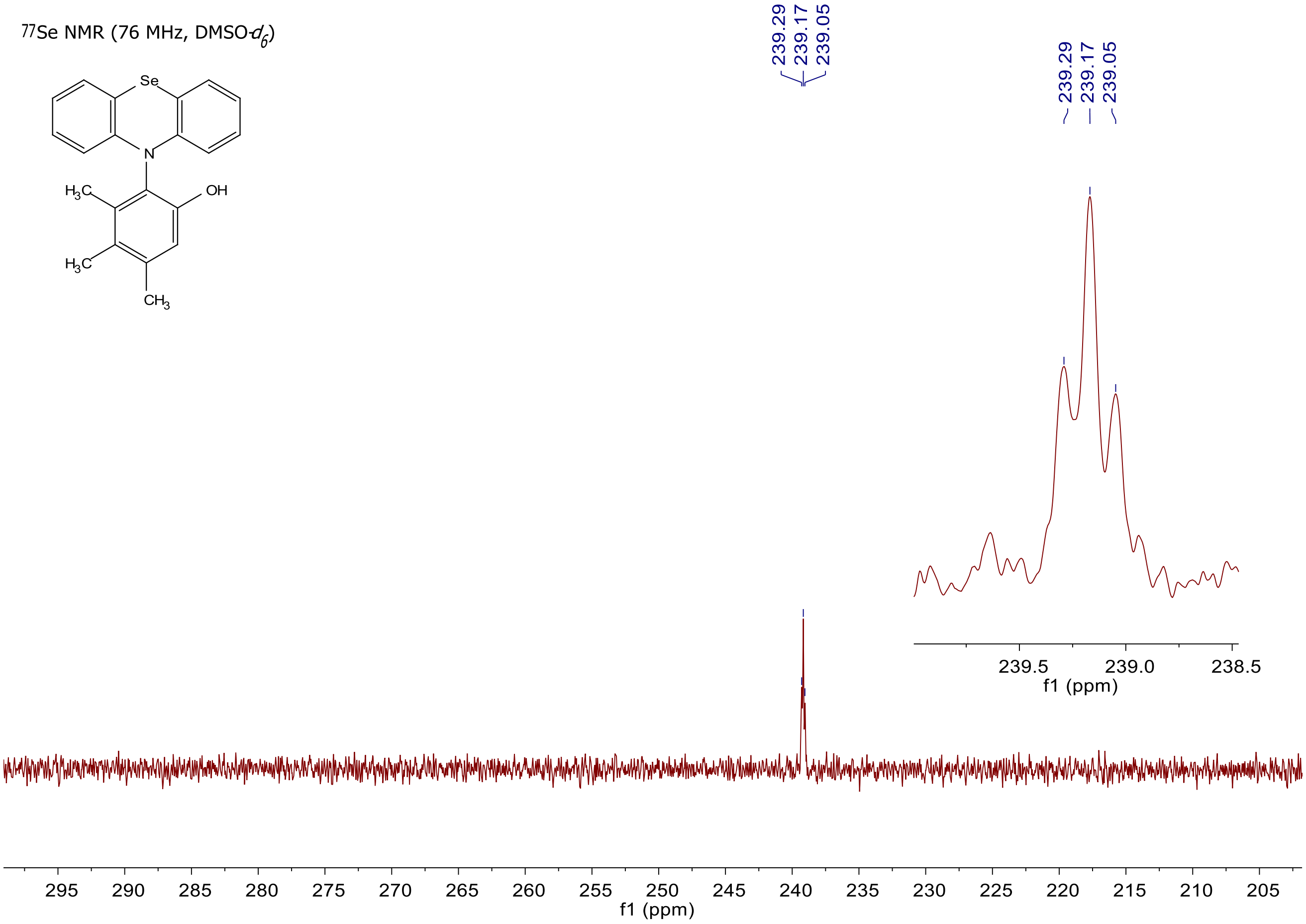

3,5-Dimethyl-2-(10*H*-phenothiazin-10-yl)phenol (*o*-3ao)

^{1}H NMR (600 MHz, DMSO-d_6)

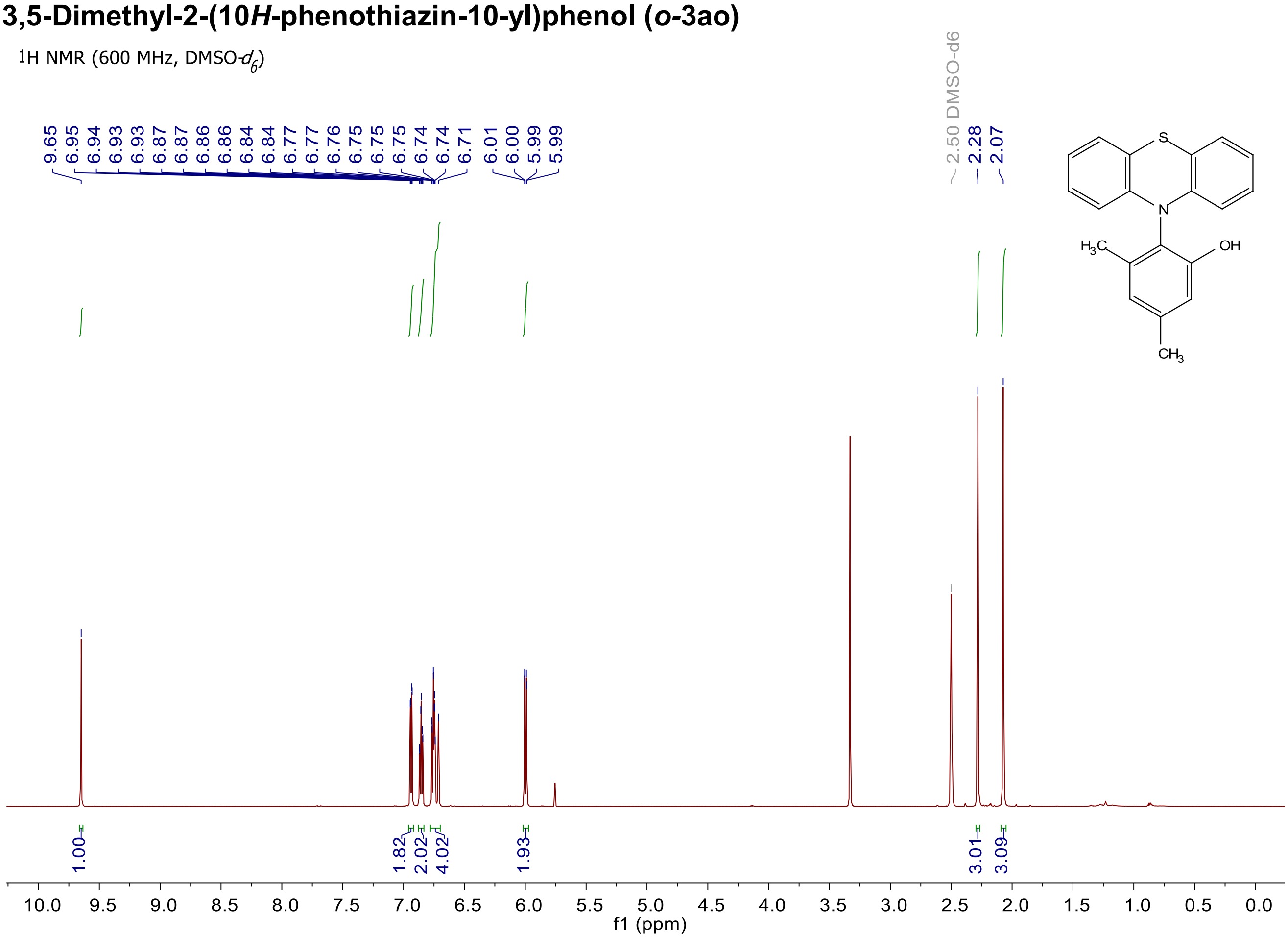

$^{13}C\{^1H\}$ NMR (151 MHz, DMSO-d_6)

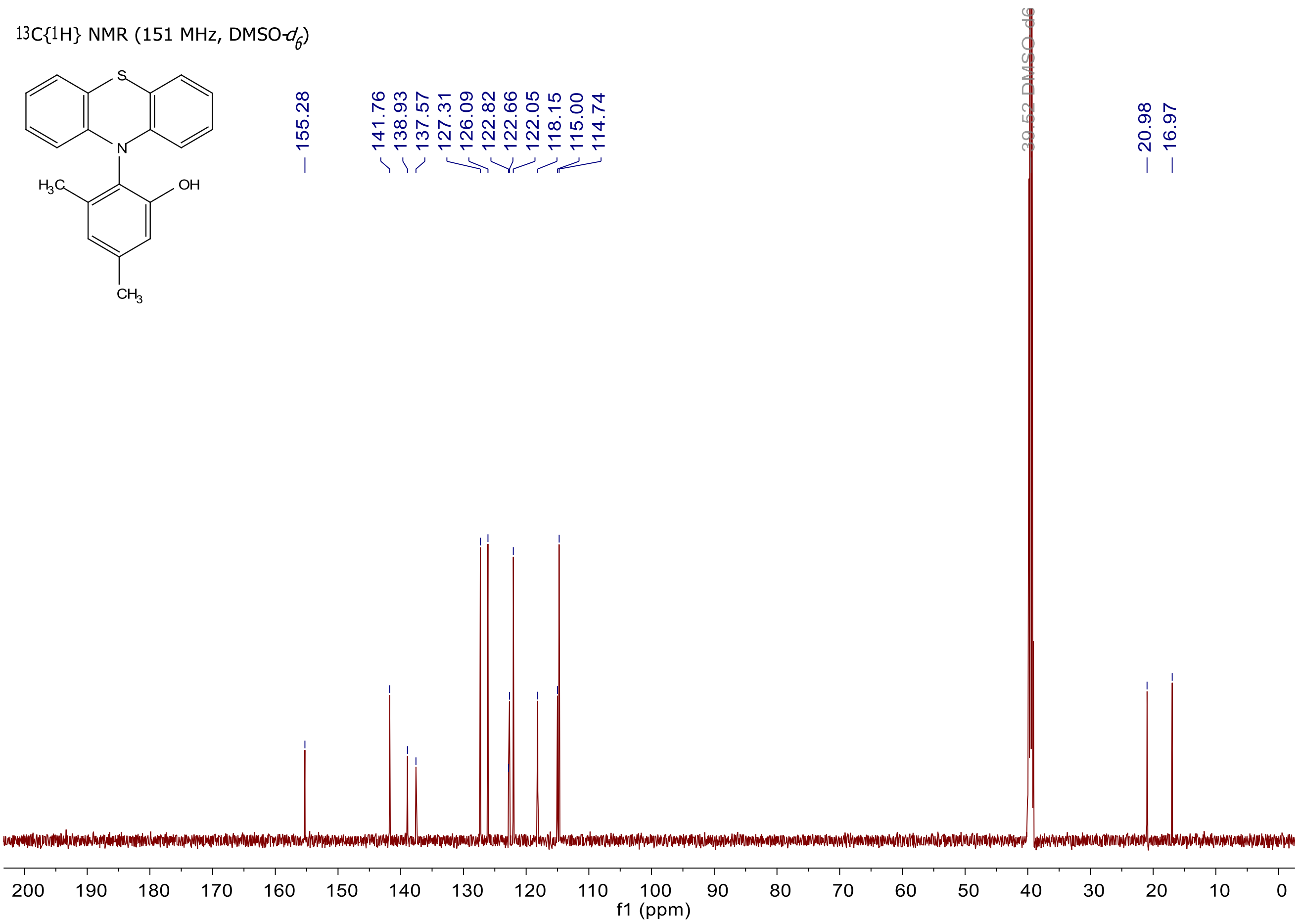

3,5-Dimethyl-4-(10*H*-phenothiazin-10-yl)phenol (*p*-3ao)

^1H NMR (600 MHz, DMSO-d_6)

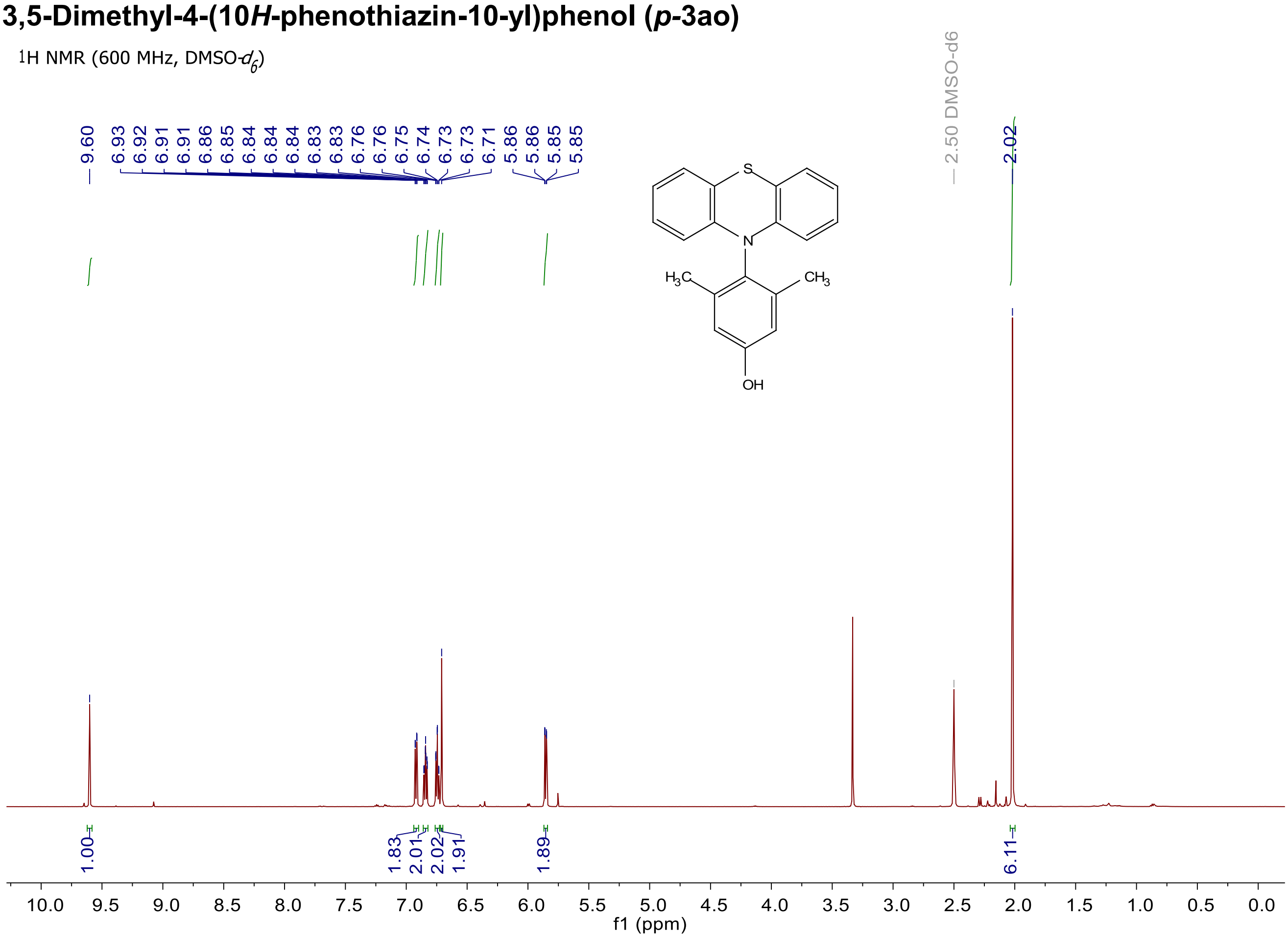

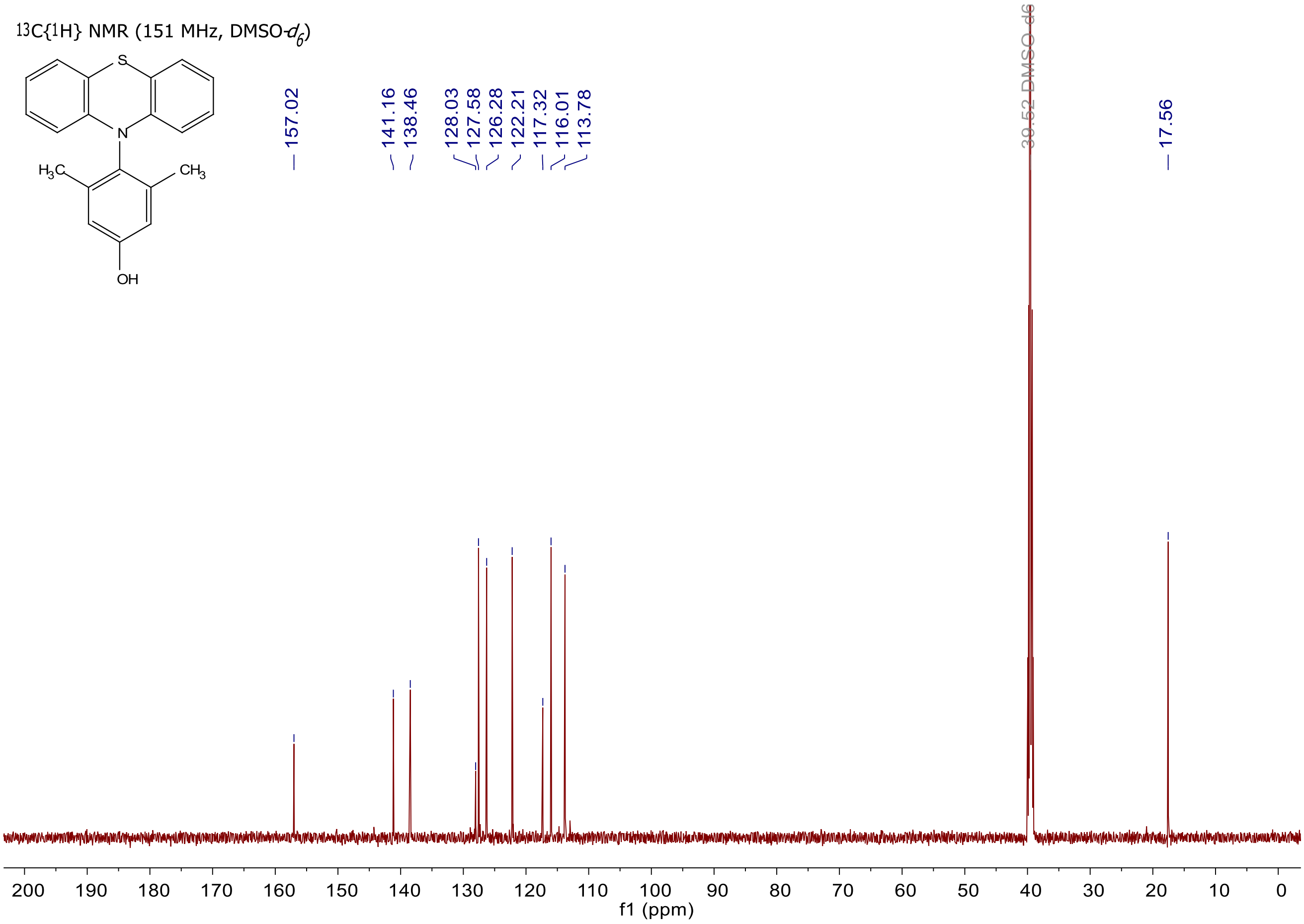

6-Isopropyl-3-methyl-2-(10*H*-phenothiazin-10-yl)phenol (*o*-3ap)

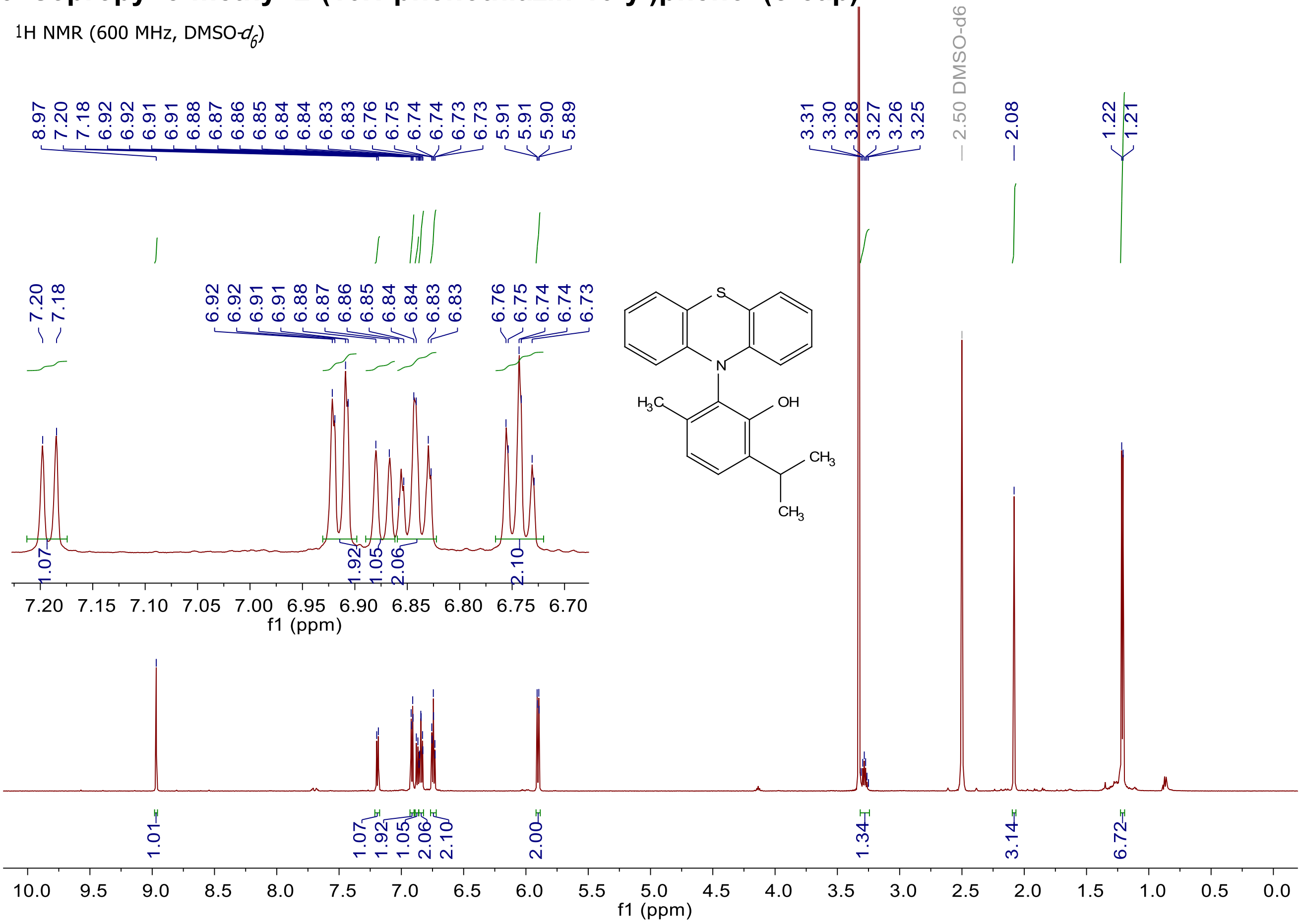

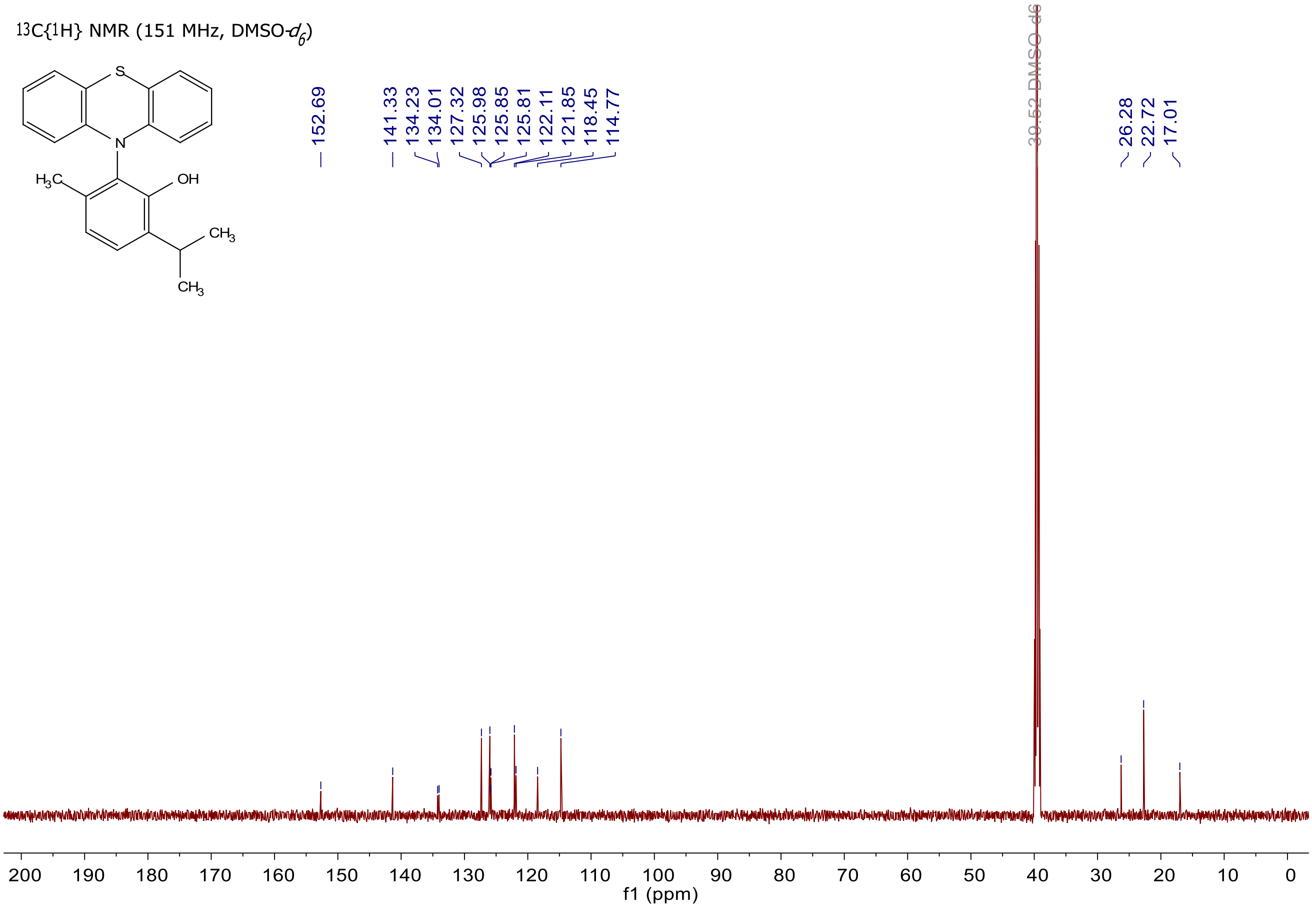

2-Isopropyl-5-methyl-4-(10*H*-phenothiazin-10-yl)phenol (*p*-3ap)

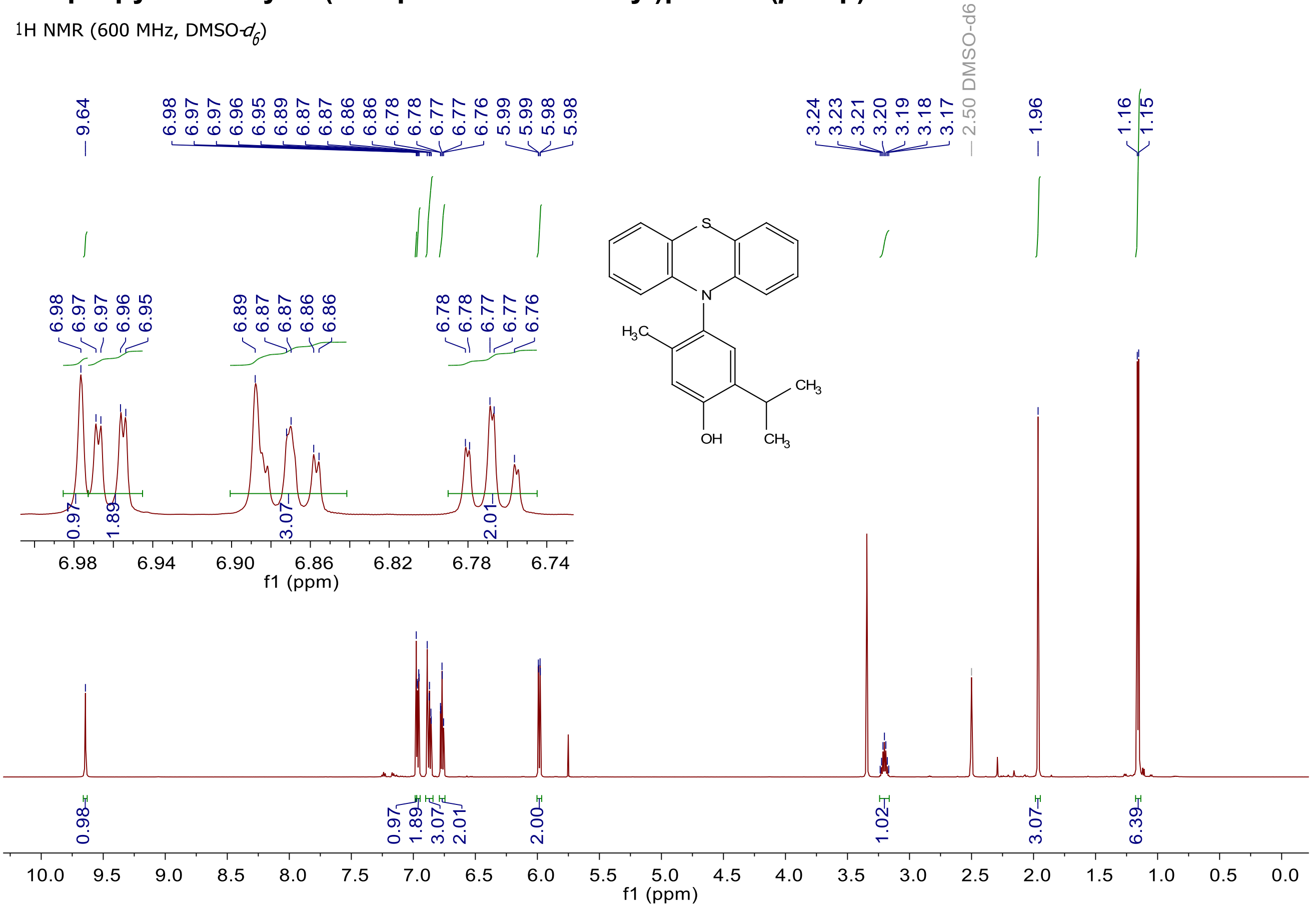

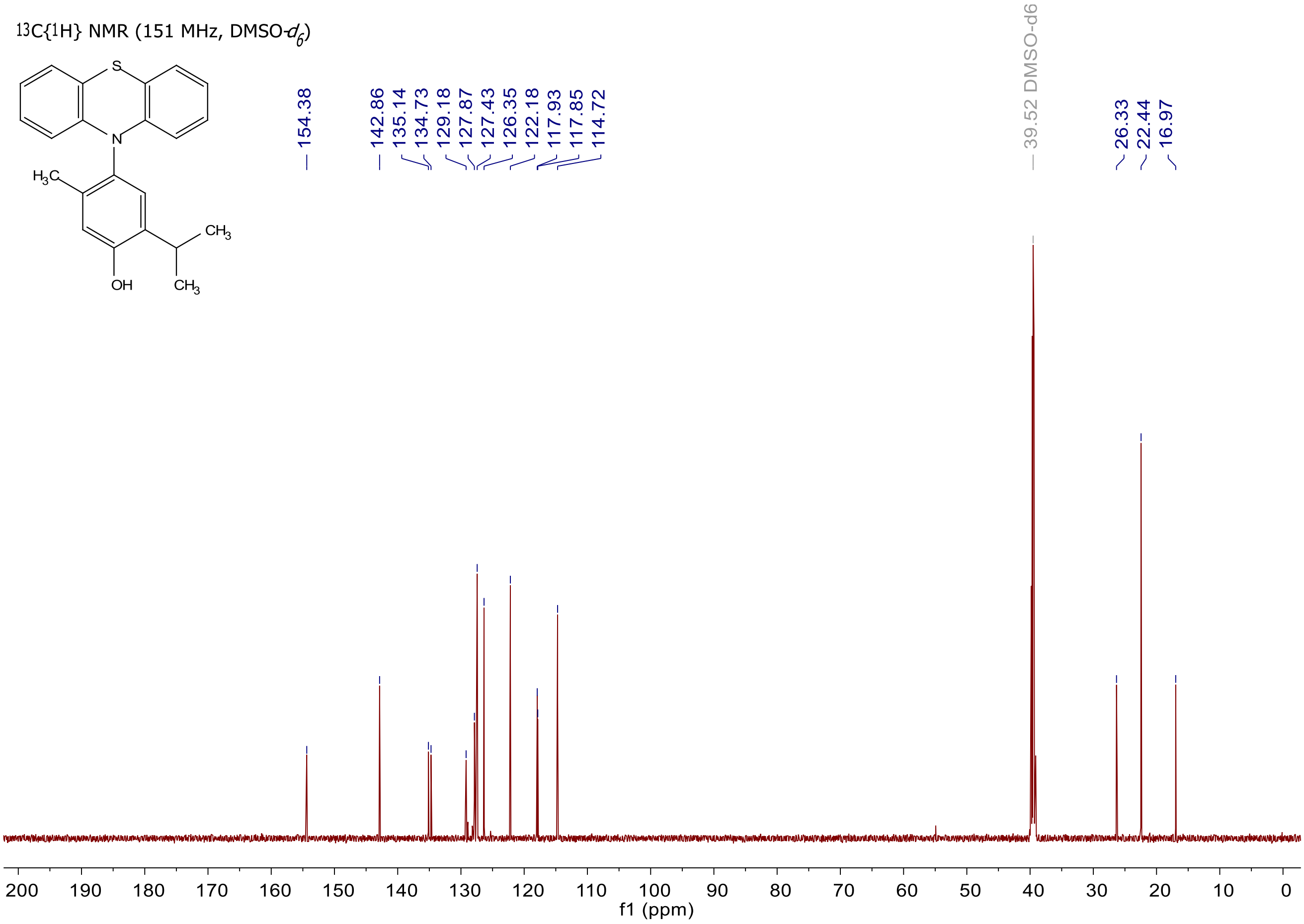

2,3-Dimethyl-6-(10*H*-phenothiazin-10-yl)phenol (*o*-3aq)

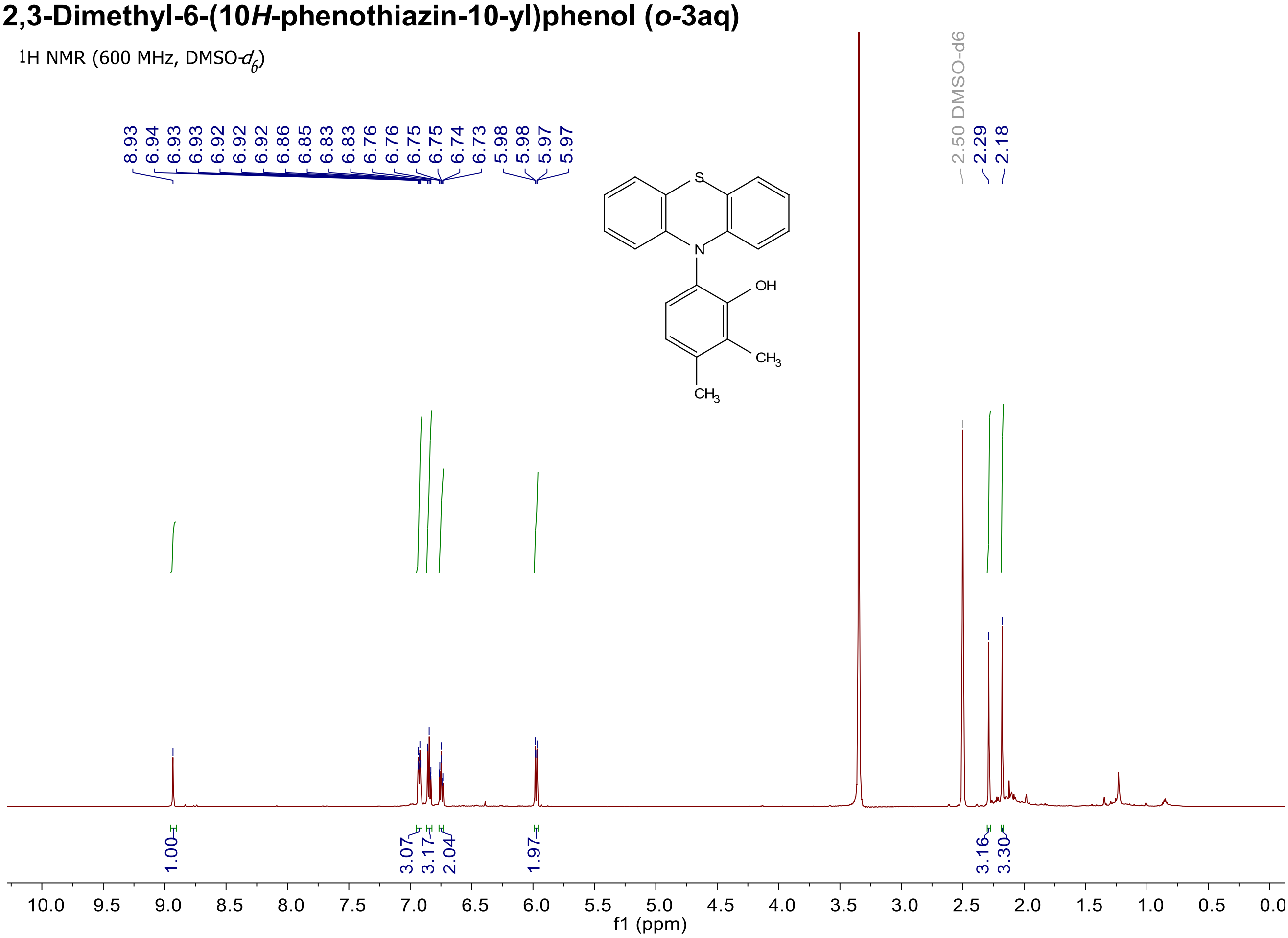

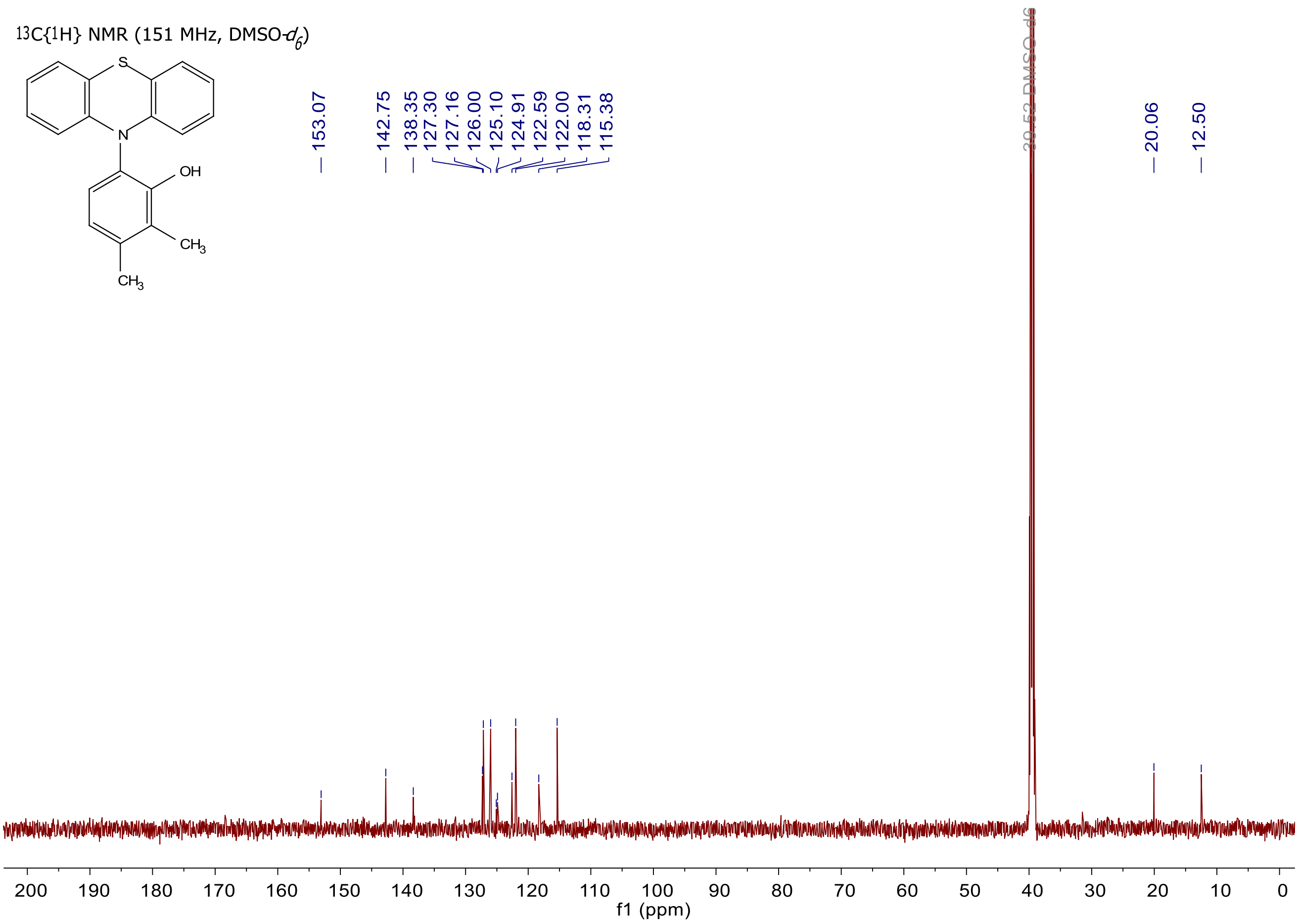

2,3-Dimethyl-4-(10*H*-phenothiazin-10-yl)phenol (*p*-3aq)

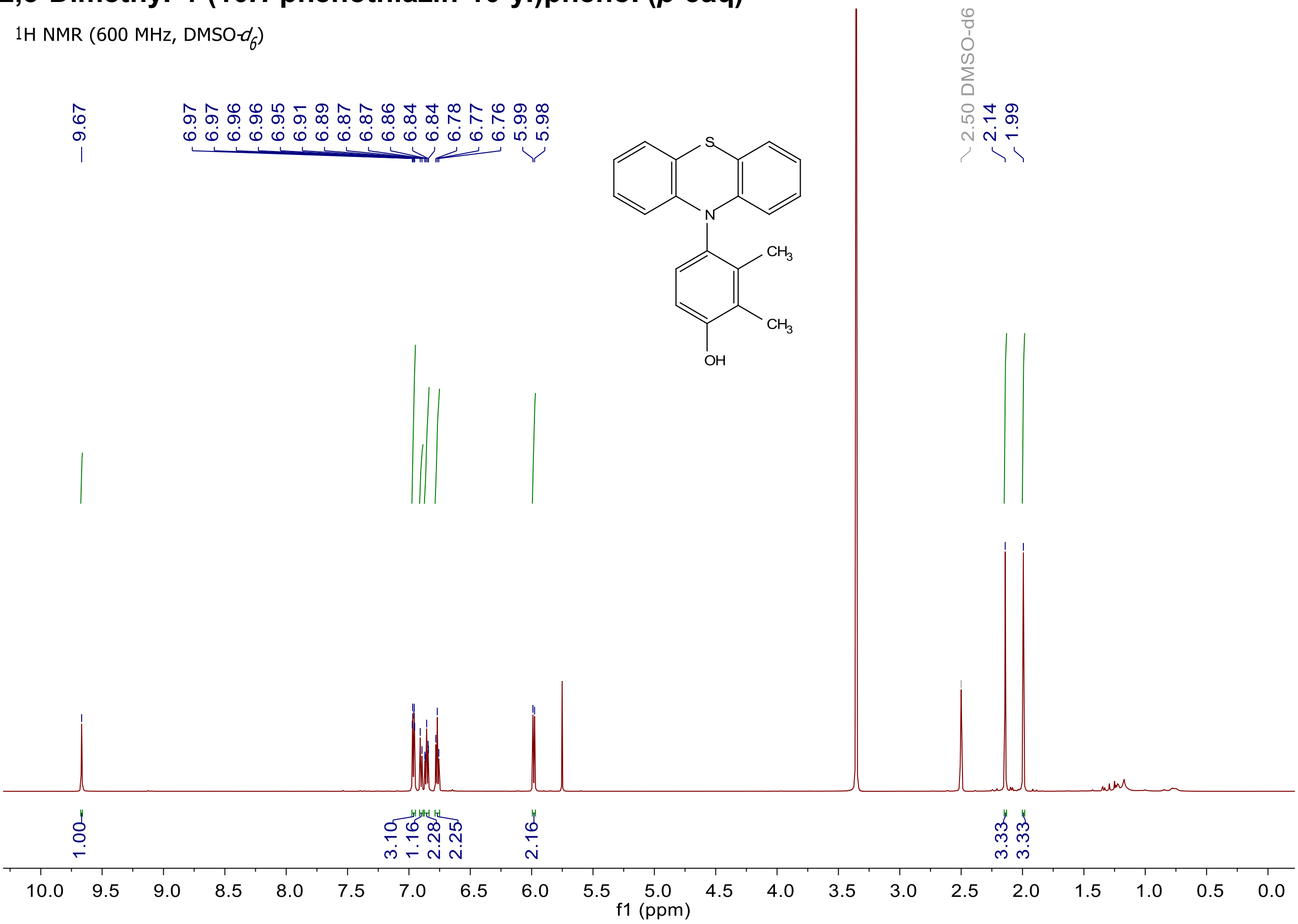

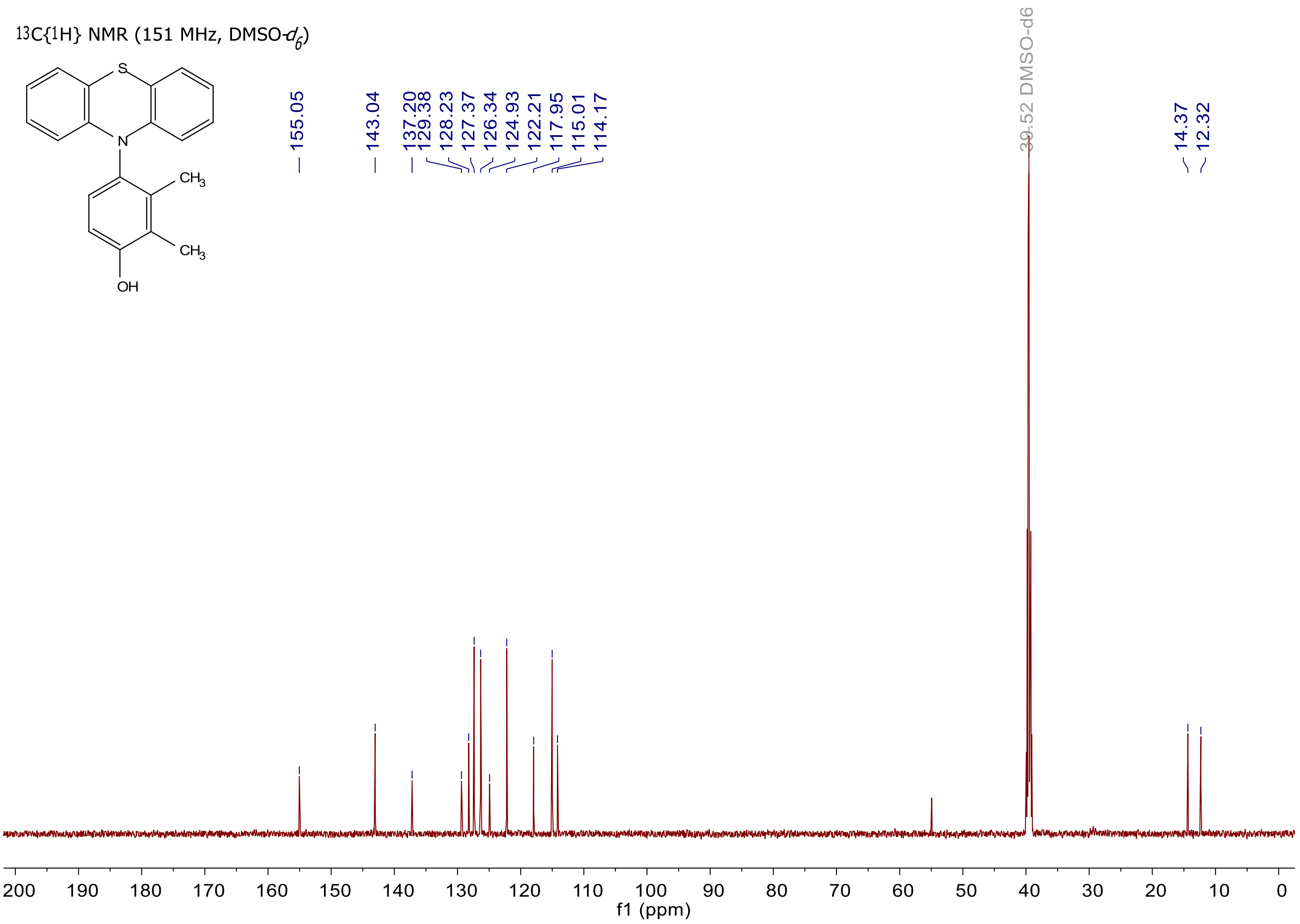

5-Isopropyl-2-methyl-4-(10*H*-phenothiazin-10-yl)phenol (3ar)

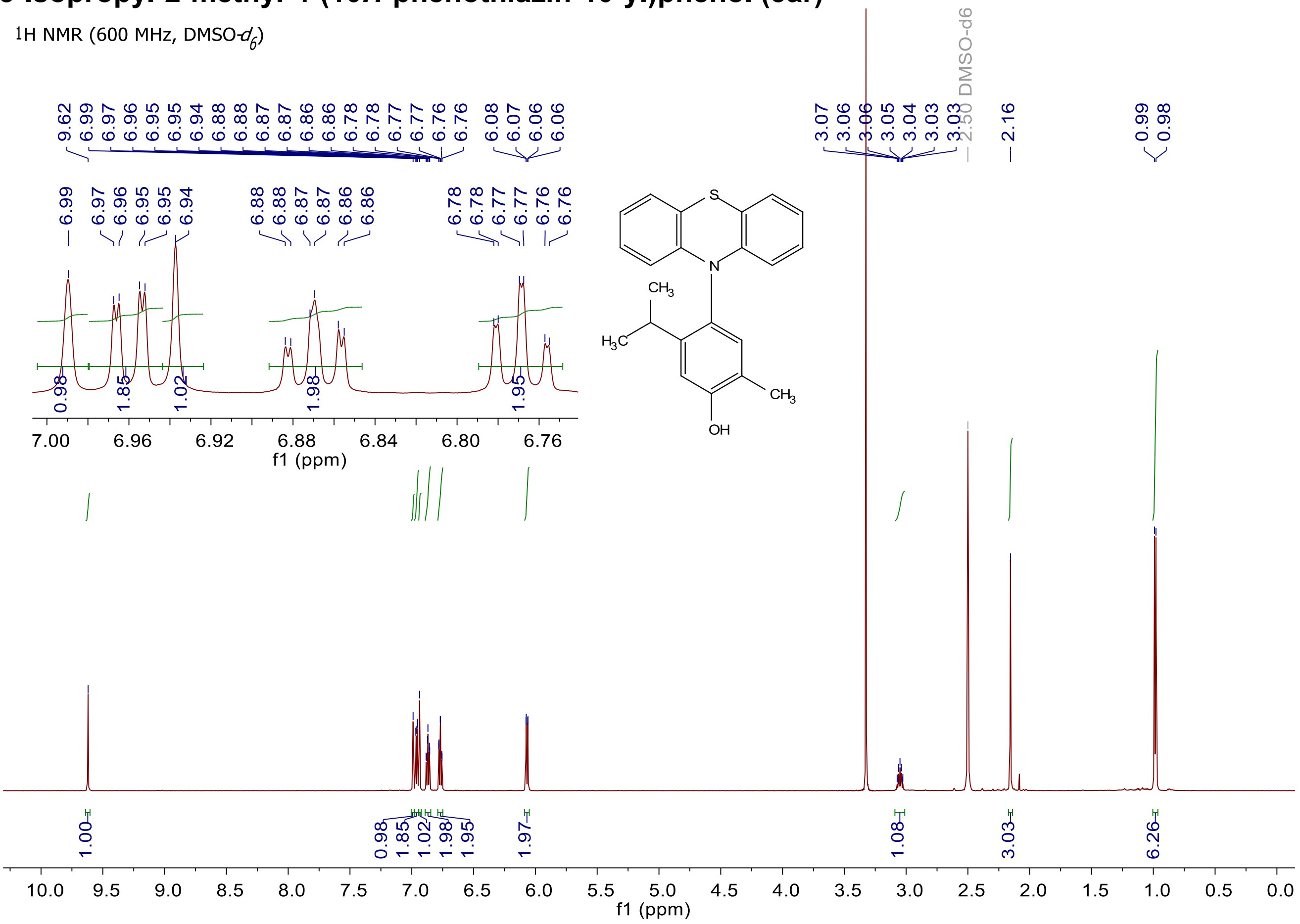

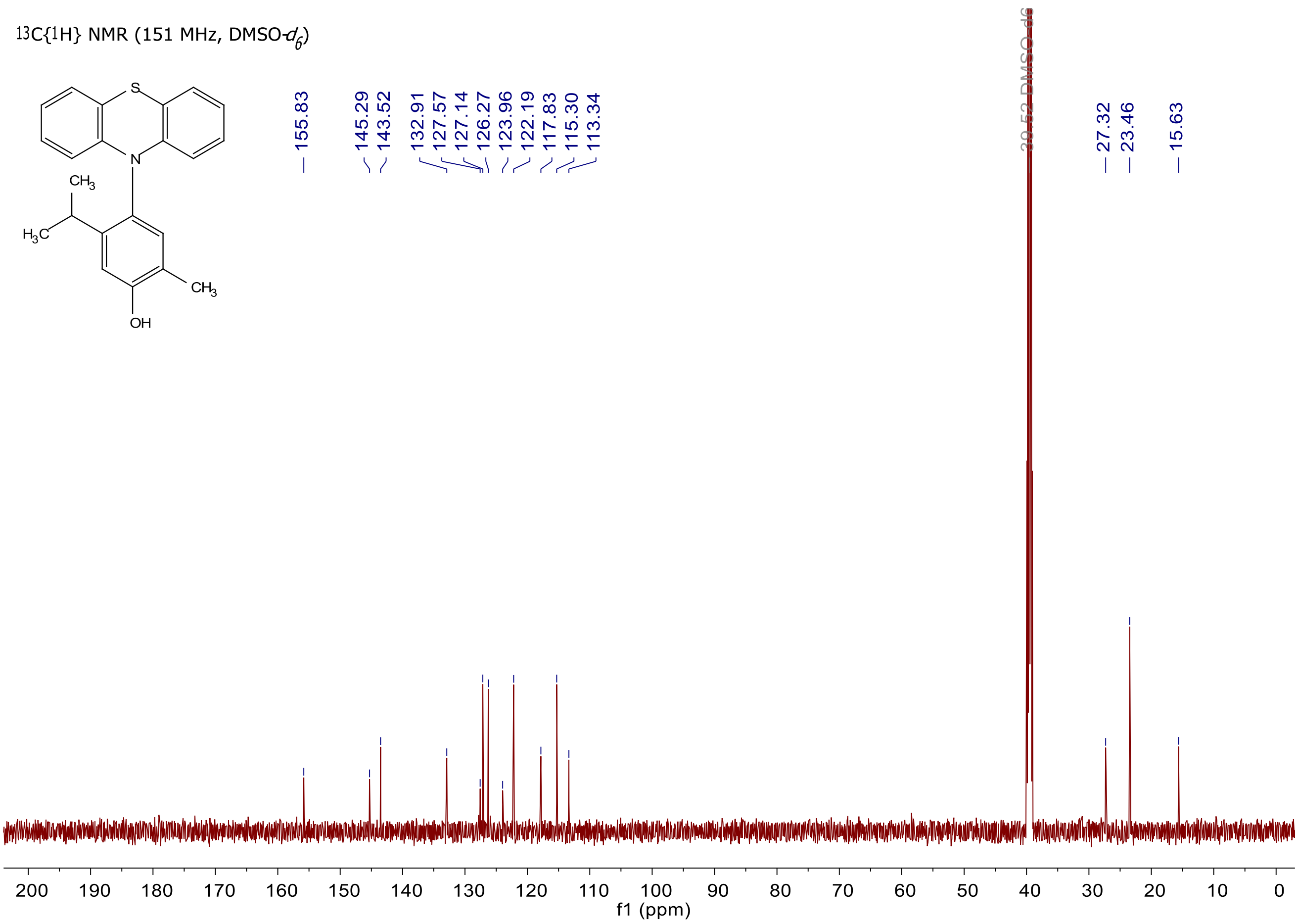

2-Methyl-4-(10*H*-phenothiazin-10-yl)phenol (3as)

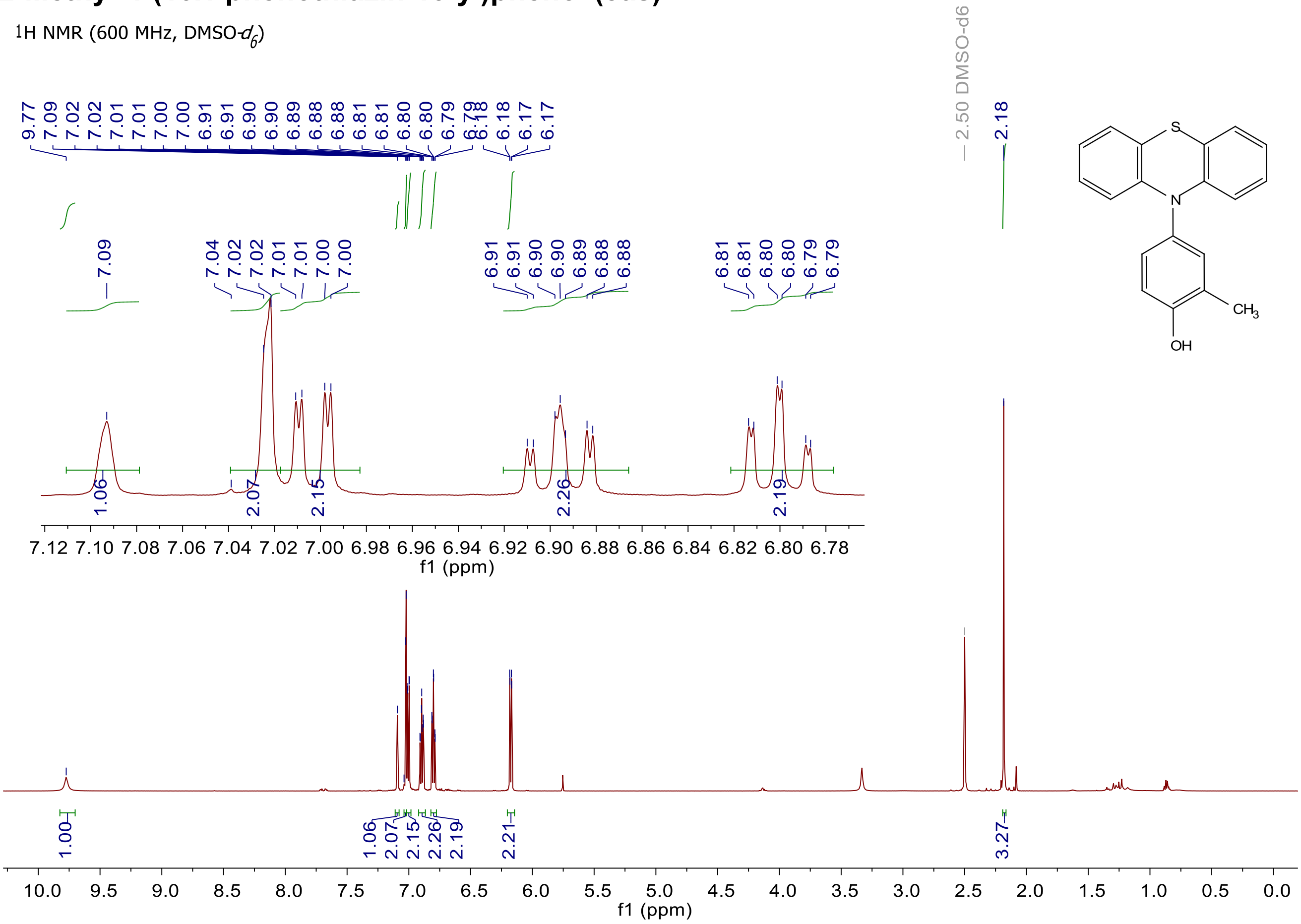

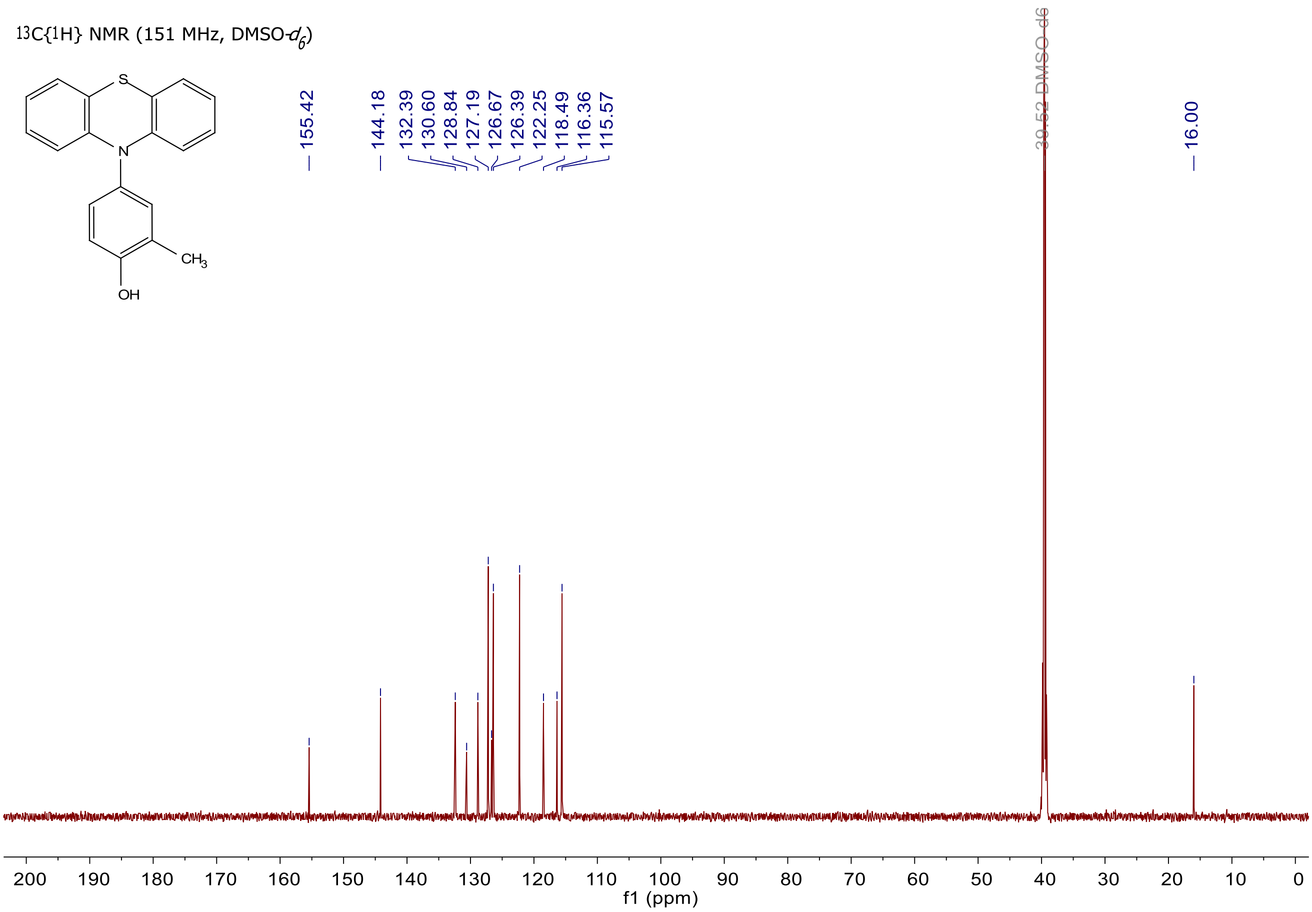

2-Methoxy-4-(10*H*-phenothiazin-10-yl)phenol (3at)

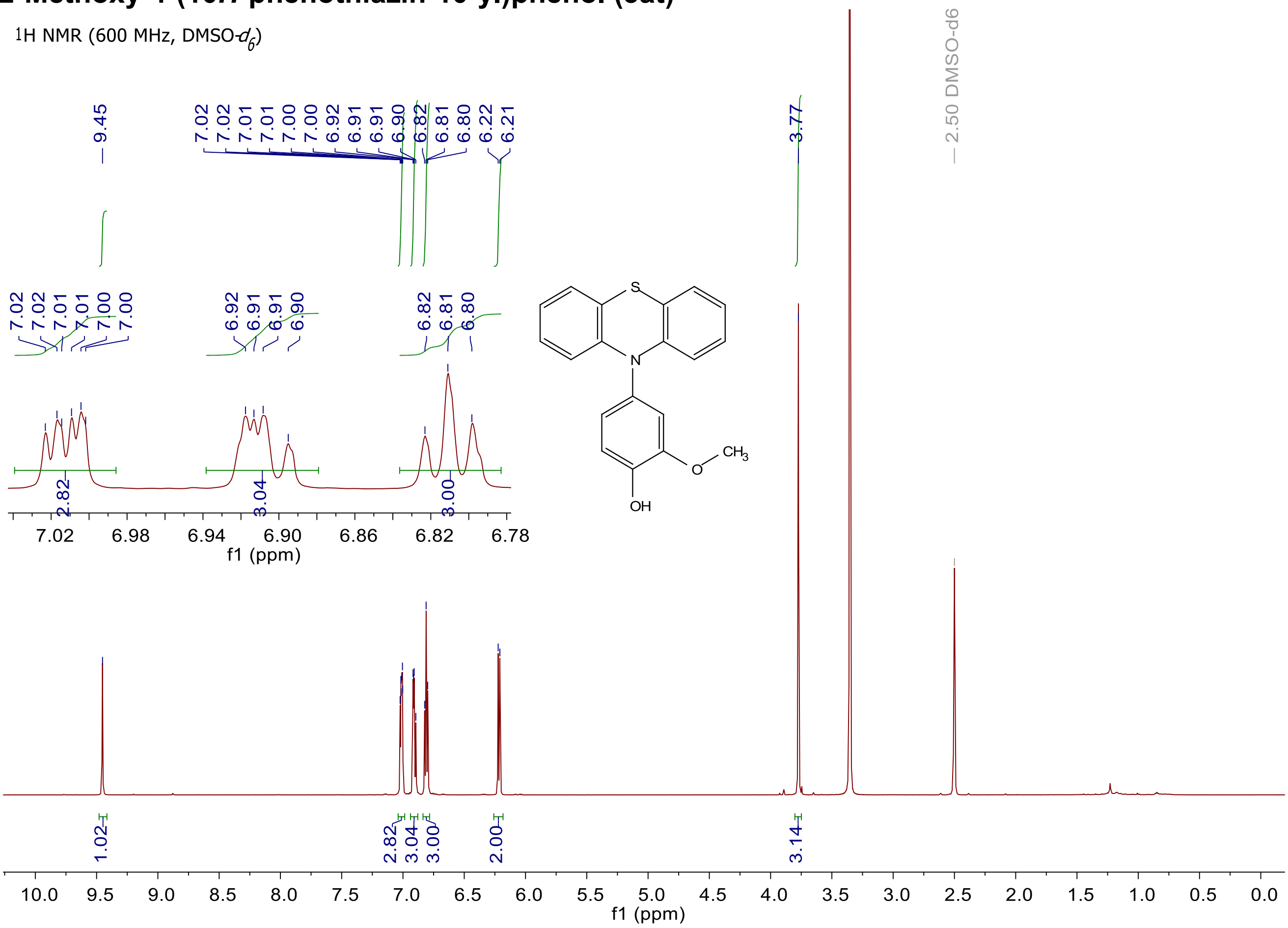

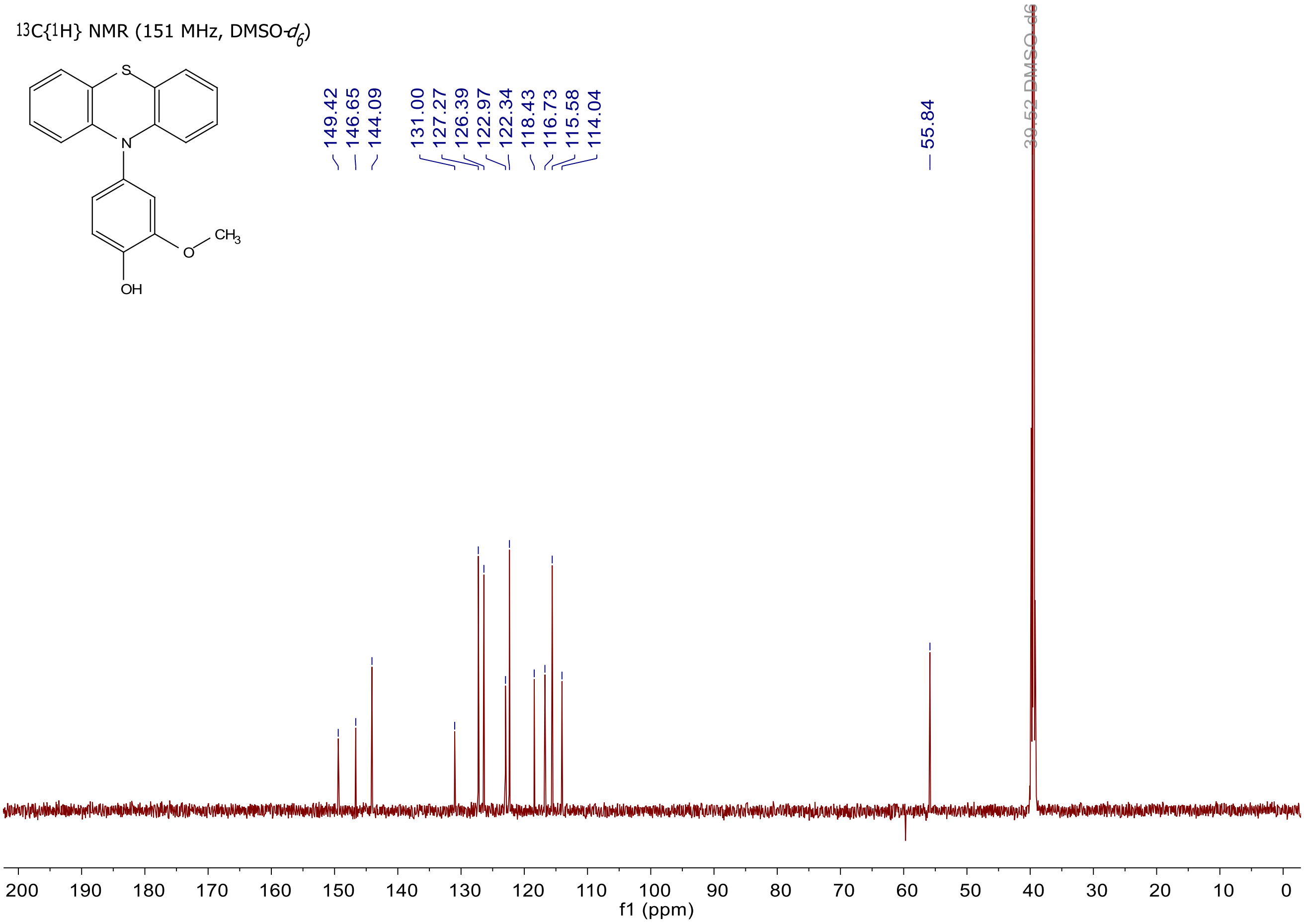

10-(2-Phenyl-1*H*-indol-3-yl)-10*H*-phenothiazine (3au)

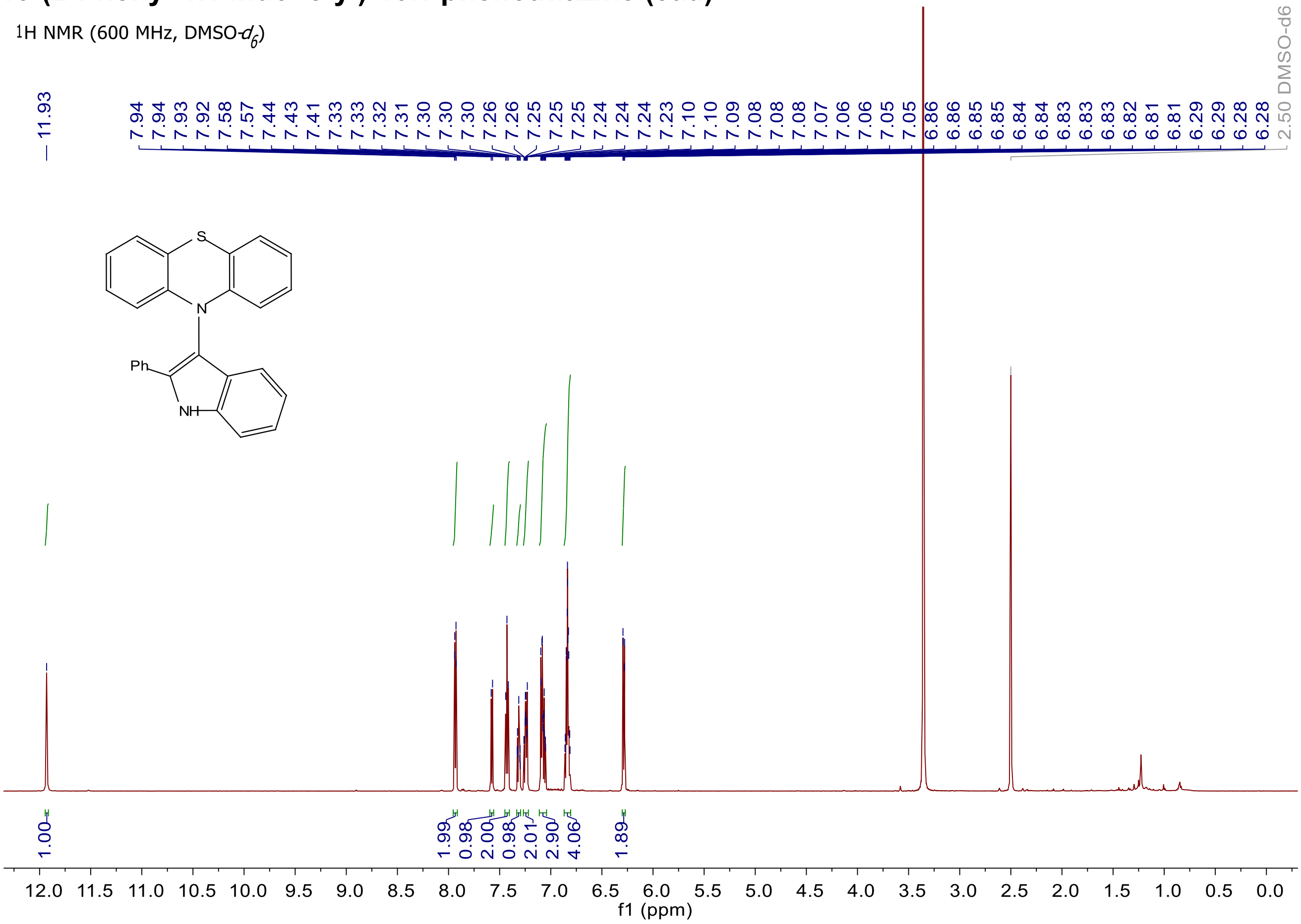

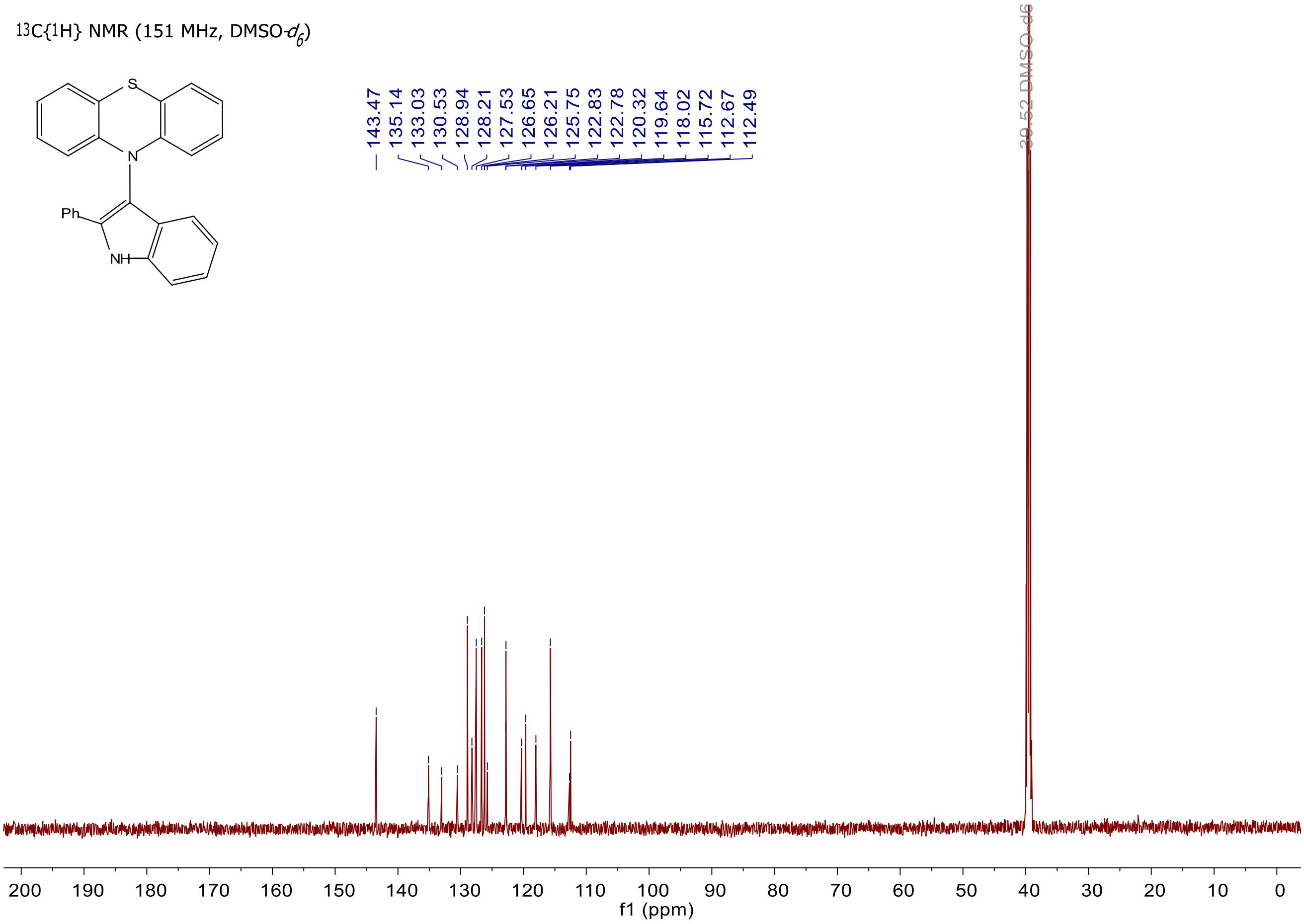

4-(10*H*-phenothiazin-10-yl)-*N*-phenylnaphthalen-1-amine (3av)

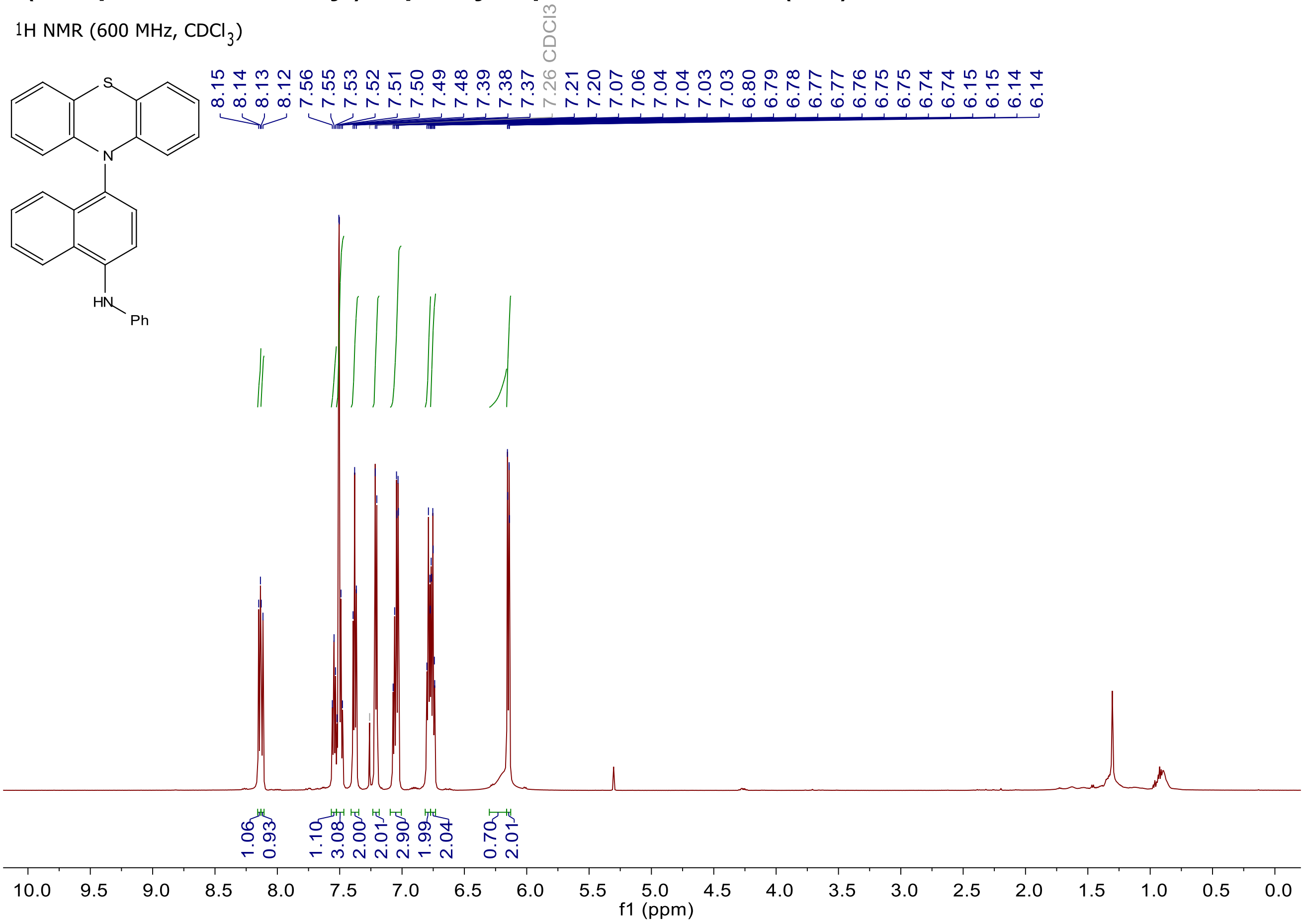

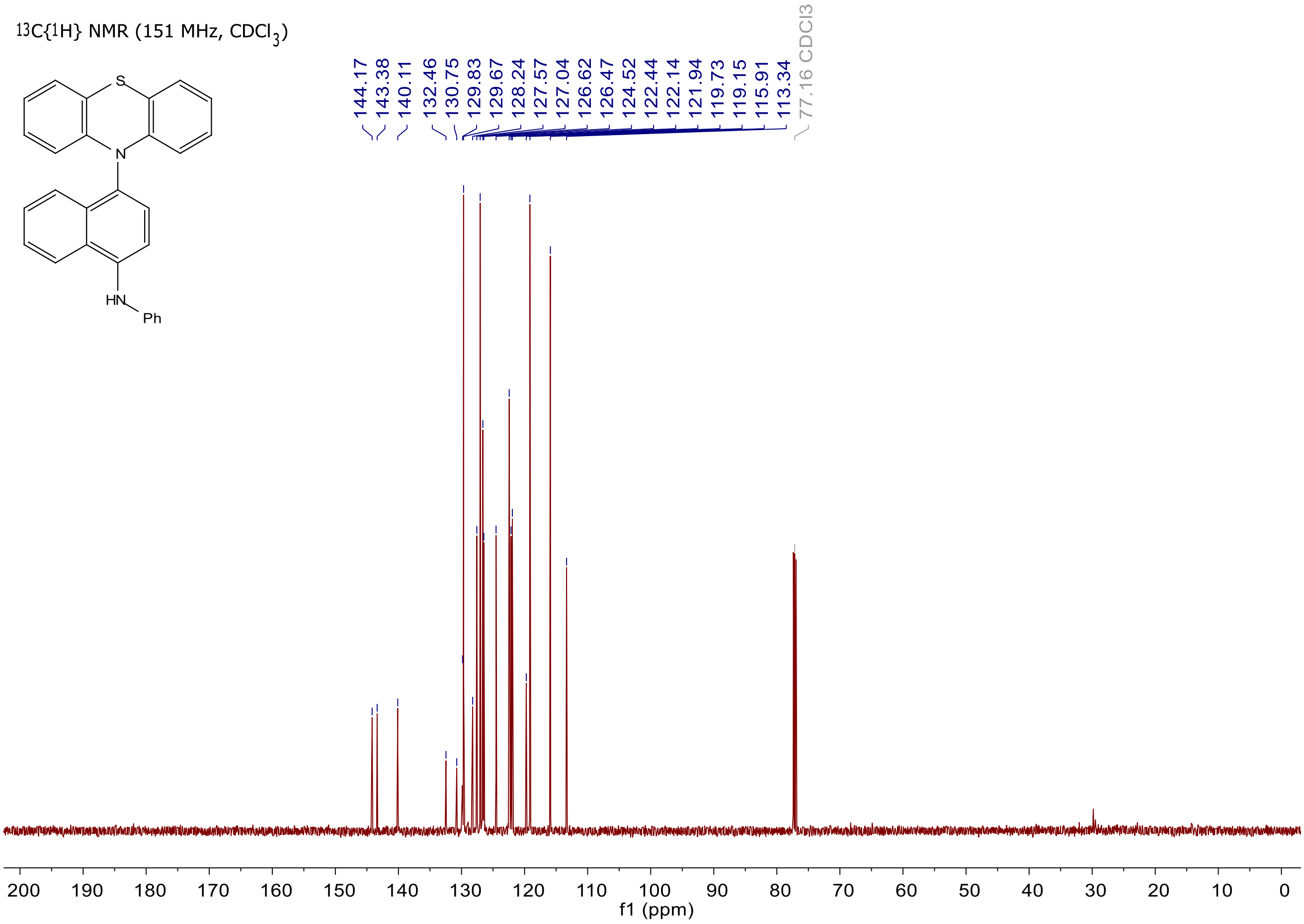

1-(10*H*-Phenothiazin-10-yl)-*N*-phenylnaphthalen-2-amine (3aw)

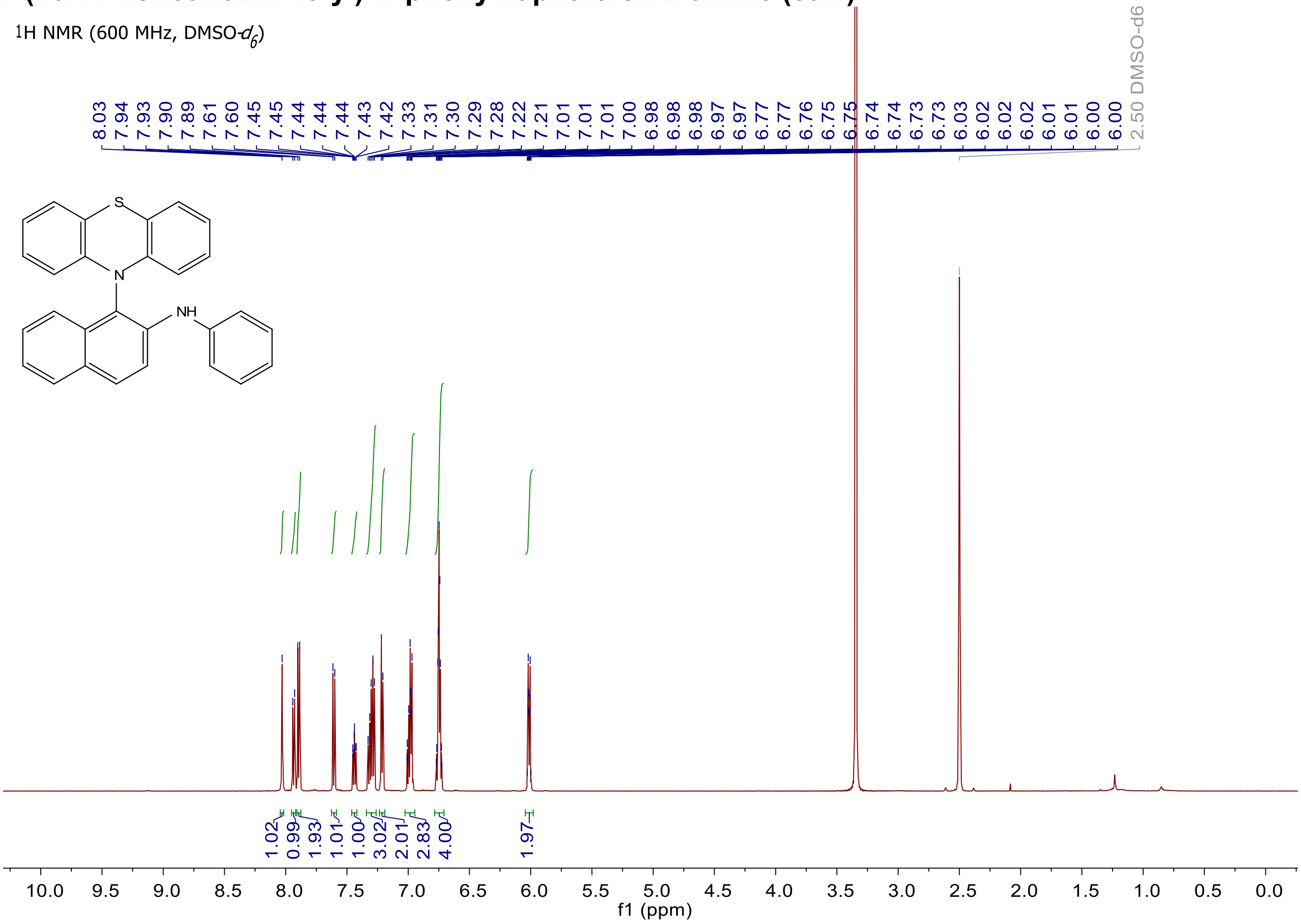

$^{13}C\{^1H\}$ NMR (151 MHz, DMSO-d_6)

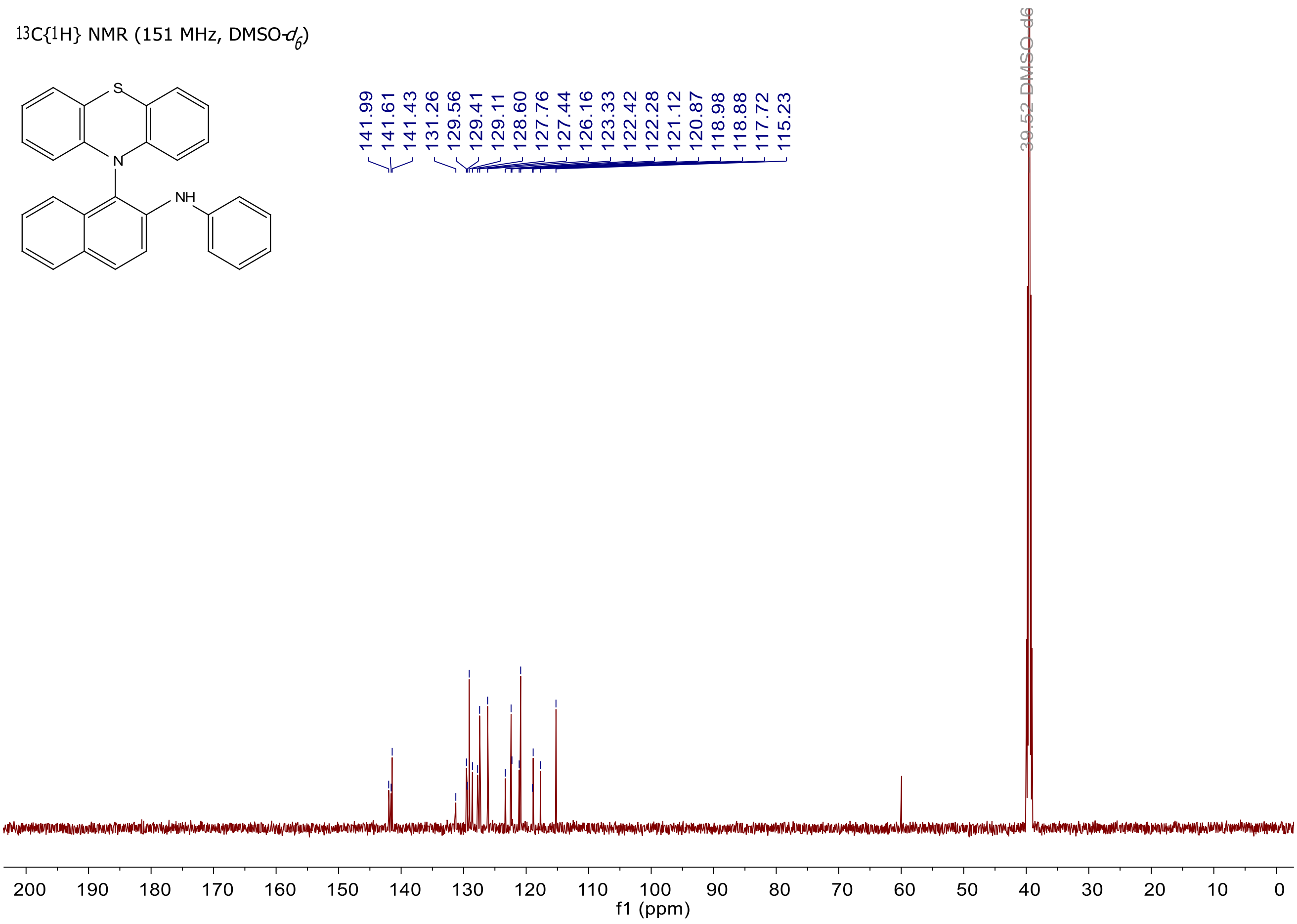

Supplement: Supplementary file 1 — The authors have cited additional references within the Supporting Information [72, 73]. The Supporting Information includes notably detailed experimental procedures, analytical data and NMR spectra. [file CSSC-19-e70801-s001.pdf]
